# Multi-Endpoint Prediction of Bioactivity and ADMET Properties Coupled with Multi-Objective Optimization in Molecular Descriptor Space for Candidate ERα Antagonists

**DOI:** 10.3390/ph19091453

**Published:** 2026-09-14

**Authors:** Yu Cui, Yuchao Qiao, Hao Ren, Lixia Qiu

**Affiliations:** 1 Department of Health Statistics, School of Public Health, Shanxi Medical University, Jinzhong 030600, China; cuiyu19951211@163.com (Y.C.); 17835152311@163.com (Y.Q.); 18435147669@163.com (H.R.); 2 Shanxi Key Laboratory of Biomedical Data and Statistics, Shanxi Medical University, Jinzhong 030600, China

**Keywords:** estrogen receptor alpha antagonists, molecular descriptors, ADMET, multi-endpoint prediction, multi-objective optimization, 2P-HAMOEA

## Abstract

**Objectives**: Candidate estrogen receptor alpha (ERα) antagonists should combine strong inhibitory activity with favorable absorption, distribution, metabolism, excretion, and toxicity (ADMET)-related properties. This study developed an integrated framework for multi-endpoint prediction, multi-objective optimization in molecular descriptor space, and prioritization of feasible solutions. **Methods**: A dataset of 1974 compounds with 729 molecular descriptors, p*IC*_50_ values, and five binary ADMET-related endpoints was analyzed. Key descriptors were identified through two-stage cross-method and cross-endpoint screening. One regression model for p*IC*_50_ and five independent classification models for the ADMET-related endpoints were developed and evaluated separately. The final selected models were incorporated into a two-phase hybrid adaptive multi-objective evolutionary algorithm (2P-HAMOEA). Feasible Pareto solutions were ranked using objective weighting, multi-criteria decision-making, reliability-based fusion, and a performance uncertainty index-based adjustment. **Results**: The final dataset comprised 1875 compounds and 24 key molecular descriptors. The three-model stacking ensemble for p*IC*_50_ achieved an *R*^2^ of 0.650 in the internal holdout set, and the AUC values of the five ADMET models ranged from 0.878 to 0.979. Under the prespecified criteria, 2P-HAMOEA generated 155 feasible Pareto solutions. Its generational distance was significantly lower than that of four comparator algorithms, whereas its inverted generational distance and hypervolume were not superior to those of most comparators. p*IC*_50_ received the highest fused weight (0.328), and pairwise rank correlations among TOPSIS, VIKOR, and WASPAS exceeded 0.90. The top 20% of RWS-ranked solutions (*n* = 31) were used for descriptor-interval analysis. **Conclusions**: The framework offers an early-stage, descriptor-level way to examine endpoint-specific predictions before alternative multi-endpoint profiles are compared. The Pareto solutions are numerical descriptor profiles rather than explicit or experimentally validated ERα antagonist structures; independent validation and molecular structure generation remain necessary.

## 1. Introduction

Breast cancer is one of the most common malignancies among women worldwide and remains a major cause of cancer-related mortality [1,2]. Estrogen receptor alpha-positive (ERα-positive) breast cancer accounts for approximately 70% of all breast cancers and is one of the most prevalent molecular subtypes. ERα-mediated estrogen signaling contributes to tumor-cell proliferation, differentiation, and migration, making ERα an important therapeutic target in breast cancer [3,4]. Endocrine therapies targeting ERα signaling have been widely used in clinical practice and have improved patient outcomes to some extent [5]. Nevertheless, acquired resistance, drug-related adverse effects, mutations in ERα-related genes, and signaling-pathway rewiring may emerge during treatment and limit the durability of therapeutic efficacy [6,7]. Early-stage identification of candidate compounds that combine potent ERα inhibitory activity with favorable pharmacokinetic properties and relatively low toxicity risk therefore remains an important research priority.

Early evaluation of candidate compounds requires consideration not only of bioactivity against the intended target but also of absorption, distribution, metabolism, excretion, and toxicity (ADMET) properties. Both bioactivity and ADMET-related properties are influenced by molecular structural features and physicochemical characteristics. Structural modifications intended to improve one property may simultaneously affect membrane permeability, metabolic behavior, bioavailability, or toxicity risk; consequently, changes across different endpoints may not occur in the same direction. Quantitative structure–activity relationship (QSAR) modeling and machine-learning methods provide computational approaches for predicting compound bioactivity and ADMET-related properties from molecular descriptors [8,9,10,11,12]. Molecular descriptor data, however, are typically high-dimensional and highly redundant and may contain complex intervariable relationships. Moreover, the structural information relevant to one prediction endpoint may differ from that relevant to another. Appropriate feature-selection and modeling strategies are therefore needed to reduce the influence of redundant information and improve the stability and predictive performance of endpoint-specific models [10,13].

When bioactivity, pharmacokinetic properties, and safety are considered simultaneously, single-endpoint prediction or single-objective optimization cannot fully characterize the overall performance of candidate compounds. Multi-objective optimization can identify Pareto-nondominated solutions across competing endpoints, allowing trade-offs among properties to be examined while multiple feasible solutions are retained [14,15,16,17]. Under complex nonlinear and constrained conditions, however, the optimization process must balance global exploration, local convergence, and solution-set diversity. Because a Pareto set generally contains several nondominated solutions with different endpoint profiles, Pareto dominance alone cannot rank them overall. Previous studies have separately examined bioactivity prediction, ADMET property modeling, and multi-objective optimization for candidate compounds with potential anti-breast-cancer activity [8,13,18,19]. However, integrated workflows that link high-dimensional molecular descriptor screening, multi-endpoint prediction, Pareto solution search, composite ranking, and descriptor-value analysis are still underdeveloped.

We therefore developed an integrated analytical workflow for candidate ERα antagonist compounds that combined key molecular descriptor selection, multi-endpoint property prediction, multi-objective optimization in molecular descriptor space, and evaluation of the resulting solutions. Data preprocessing and a two-stage cross-method and cross-endpoint screening procedure were first used to identify key molecular descriptors repeatedly selected across different methods and multiple endpoints. Regression models for p*IC*_50_ and classification models for five ADMET-related endpoints were then developed, and the final selected models were incorporated into the multi-objective optimization procedure. Each endpoint was modeled independently; the term multi-endpoint refers to the overall workflow and the subsequent integration of six endpoint-specific predictions, rather than to fitting bioactivity and ADMET outcomes in a single predictive model. A two-phase hybrid adaptive multi-objective evolutionary algorithm (2P-HAMOEA) was then used to generate feasible Pareto solutions, which were ranked using objective weighting, multi-criteria decision-making, reliability-based fusion, and performance-uncertainty adjustment. The top 20% of the feasible Pareto solutions according to the robust weighted score (RWS) defined the high-scoring subset, from which statistical value intervals for the key molecular descriptors were derived. The workflow was designed to provide an early-stage, descriptor-level reference for examining activity-ADMET trade-offs and comparing alternative computational profiles. It was not intended to generate directly synthesizable molecules or substituent-level structure–activity rules.

## 2. Results

### 2.1. Data Preprocessing, Selection of Key Molecular Descriptors, and Dataset Partitioning

The original dataset contained 1974 compounds and 729 molecular descriptors. Low-variance filtering removed 228 descriptors, leaving 501 for further analysis; no missing values were identified among the retained variables. Correlation-based redundancy reduction, using an absolute Spearman rank correlation coefficient threshold of 0.95, removed a further 242 highly correlated descriptors and left 259 descriptors. Isolation forest identified 99 outlier samples, which were excluded, yielding a final analytical dataset of 1875 compounds. The remaining 259 molecular descriptors were min–max normalized and subsequently used for key descriptor selection and prediction model development. We then examined all 276 descriptor pairs in the final 24-descriptor set. None exceeded the prespecified absolute Spearman correlation threshold of 0.95; the largest absolute correlation was 0.909 between LipoaffinityIndex and MLogP. Although this pair was strongly correlated, the two variables are calculated using different descriptor formulations and represent related but non-identical aspects of lipophilicity. Each also satisfied the cross-endpoint consensus rule; retaining both therefore reflects repeated relevance across endpoints, not an assumption of statistical independence.

**Figure 1:**
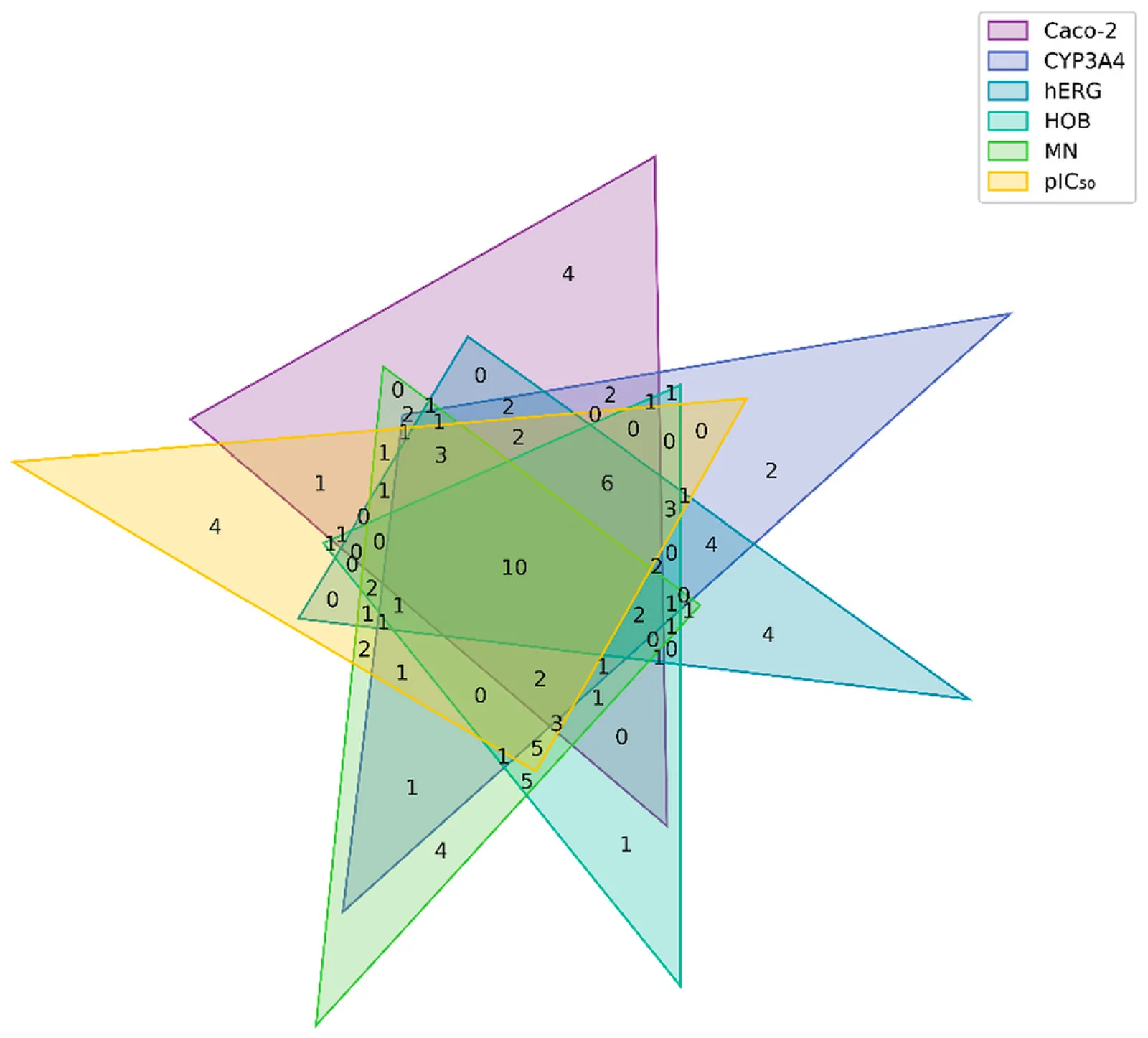
Cross-endpoint consistency of the key molecular descriptors across the six prediction endpoints. Different colors represent the candidate descriptor sets identified for the individual endpoints. Overlapping regions indicate descriptors selected for multiple endpoints, and the numbers indicate the counts of descriptors in the corresponding regions.

Descriptor rankings differed across RF, AE, SC, SVM-RFE, and MIC. The candidate feature sets showed both overlap and method-specific variation. Comparison of the five method-specific feature sets within each endpoint, followed by comparison across p*IC*_50_, Caco-2, CYP3A4, hERG, HOB, and MN, identified 24 key molecular descriptors that were retained for at least five endpoints (Figure 1). These descriptors captured structural or physicochemical information related to molecular autocorrelation, topology, electronic properties, polarity, hydrogen bonding, functional groups, chemical bonds, and lipophilicity. They were used as a common input set for the p*IC*_50_ regression model, the five ADMET classification models, and the subsequent multi-objective optimization in molecular descriptor space. The 24 descriptors and the structural or physicochemical information encoded by their computational definitions are listed in Table 1.

The same fixed assignment of 1500 training compounds and 375 internal-holdout compounds was used for p*IC*_50_ and all five ADMET endpoints. In the joint t-SNE visualization, most internal-holdout compounds occupied regions also populated by training compounds, while internal-holdout compounds were more prominent in several peripheral regions, as expected from maximum-dissimilarity selection (Figure S3). In the formal descriptor-space AD check, 323 of 375 internal-holdout compounds (86.1%) had leverage at or below h* = 0.050, whereas 52 compounds (13.9%) exceeded the warning leverage (Table S9). All performance estimates used the full 375-compound internal holdout rather than an AD-restricted subset. These results characterize coverage in the selected descriptor space; neither the joint embedding nor leverage analysis demonstrates generalization to new scaffolds or externally sourced chemotypes.

Across the five ADMET classification tasks, class imbalance ratios ranged from 1.23:1 to 3.59:1 in the training sets and from 1.45:1 to 3.63:1 in the internal holdout sets. Caco-2 and hERG were comparatively balanced, whereas CYP3A4, HOB, and MN showed varying degrees of class imbalance. In the training sets, the imbalance ratios for HOB and MN were 3.59:1 and 3.57:1, respectively. In the internal holdout sets, the corresponding ratios for CYP3A4 and MN were 3.31:1 and 3.63:1. Cost-sensitive learning was therefore introduced during the training of selected candidate models, while the original class distributions were preserved in the internal holdout sets.

### 2.2. Predictive Performance for Bioactivity and ADMET-Related Endpoints

#### 2.2.1. Performance of the p*IC*_50_ Regression Models

Tenfold cross-validation within the training set revealed differences in predictive performance among the candidate regression models. The TPE-optimized Oblivious Tree model showed comparatively strong overall performance, with an MAE of 0.515 ± 0.056, a MedAE of 0.369 ± 0.040, an MSE of 0.496 ± 0.112, and an *R*^2^ of 0.741 ± 0.055. This model was carried forward for comparison in the internal holdout set. Internal-holdout results for the seven candidate regression families and the principal three-model ensemble alternatives are reported in Supplementary Table S5.

In the internal holdout set, the three-model stacking ensemble achieved an MAE of 0.573, a MedAE of 0.421, an MSE of 0.627, and an *R*^2^ of 0.650 (95% bootstrap CI, 0.549–0.728). Its first-layer base learners were Oblivious Tree + TPE, SVR + TPE, and XGBoost + TPE, and linear regression served as the second-layer meta-learner. In the original model-selection workflow, the three-model stacking ensemble had the largest radar area among the training-set ensemble configurations (8.081) and the internal-holdout candidates (8.820), and it achieved the lowest MAE, MedAE, and MSE together with the highest *R*^2^ in the internal holdout. It was selected as the final model for p*IC*_50_.

In the matched descriptor-set comparison, the same three-model stacking architecture achieved an *R*^2^ of 0.6467 with the current 24 descriptors and 0.6465 with the Dong et al. 20-descriptor set. The paired *R*^2^ difference was 0.00015 (95% bootstrap CI, −0.0478 to 0.0466), and the corresponding MAE values were 0.575 and 0.585. The matched analysis found no material difference between descriptor sets and did not show that either set was superior; the formal final-model result remains the value reported above (Supplementary Table S4).

#### 2.2.2. Performance of the ADMET Classification Models

The final selected model varied across the five ADMET-related endpoints. For Caco-2, LightGBM + TPE achieved an accuracy of 0.867, precision of 0.851, recall of 0.805, AUC of 0.933, and F_1_ score of 0.828 in the internal holdout set.

For CYP3A4, MLP + TPE + CS was selected, with an accuracy of 0.904, precision of 0.921, recall of 0.959, AUC of 0.936, and F_1_ score of 0.939.

For hERG, the final model was a five-model simple averaging ensemble comprising GB + TPE, XGBoost + TPE, LightGBM + TPE, BPNN + TPE, and SVM. This ensemble achieved an accuracy of 0.829, precision of 0.805, recall of 0.936, AUC of 0.878, and F_1_ score of 0.866.

For HOB, GB + TPE yielded an accuracy of 0.797, precision of 0.786, recall of 0.682, AUC of 0.880, and F_1_ score of 0.730. For MN, LightGBM + CS achieved corresponding values of 0.933, 0.938, 0.980, 0.979, and 0.958.

**Figure 2:**
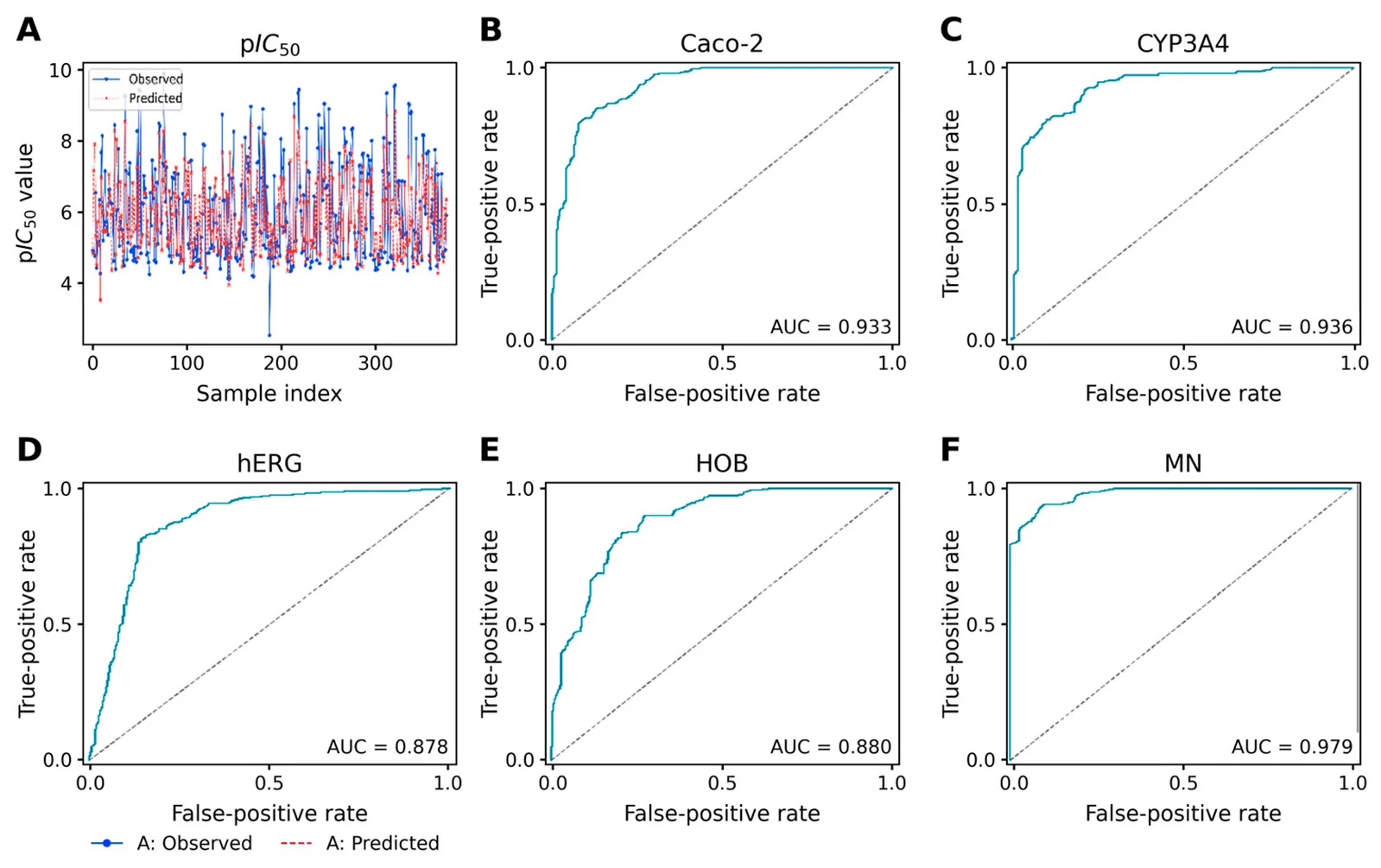
Predictive performance of the final selected models in the internal holdout sets. (**A**) Observed versus predicted p*IC*_50_ values obtained using the final selected three-model stacking ensemble; (**B**–**F**) receiver operating characteristic curves for the final selected models for Caco-2, CYP3A4, hERG, HOB, and MN, respectively. For hERG, the curve is based on the simple average of the five binary constituent-model predictions used for classification evaluation. Bootstrap 95% confidence intervals for the corresponding metrics are reported in Supplementary Table S8. In panel (**A**), the red dashed line represents predicted values and the blue solid line represents observed values. In panels (**B**–**F**), the diagonal dashed line represents the no-discrimination reference.

Across the five ADMET-related endpoints, the final selected models achieved accuracies ranging from 0.797 to 0.933, AUC values from 0.878 to 0.979, and F_1_ scores from 0.730 to 0.958 in the internal holdout sets. An ensemble model was selected only for hERG; the remaining four endpoints were modeled using individual algorithms with hyperparameter optimization and/or cost-sensitive learning. The shortlisted candidate results are reported in Supplementary Table S6, and tenfold cross-validation mean ± standard deviation values for the selected individual models and the five hERG constituent learners are reported in Supplementary Table S7. Bootstrap 95% confidence intervals for all final internal-holdout metrics are provided in Supplementary Table S8. They should not be interpreted as external-validation evidence. The corresponding precision–recall curves and confusion matrices are provided in Supplementary Figures S1 and S2, respectively. The final internal-holdout performance of the six selected endpoint-specific models is summarized in Table 2 and Figure 2.

### 2.3. Algorithm Validation and Ablation Results for 2P-HAMOEA

#### 2.3.1. Benchmark-Function Evaluation

The relative performance of 2P-HAMOEA varied across the benchmark functions. For the ZDT1, ZDT2, and ZDT3 two-objective functions, its mean runtimes were 1.563 ± 0.101, 1.606 ± 0.054, and 1.623 ± 0.093 s, respectively, all of which were lower than those of the corresponding comparator algorithms. This runtime advantage, however, was not accompanied by consistently better HV or IGD values. Its coverage of the objective space and convergence toward the reference Pareto front therefore depended on the specific benchmark function.

For ZDT2, the distribution-dispersion metric $\Delta_{p}$ was 0.505 ± 0.156, numerically close to the value of 0.501 ± 0.001 obtained using NSGA-III. The corresponding HV and IGD values for 2P-HAMOEA were 0.271 ± 0.145 and 3.619 ± 0.177, respectively.

Performance also varied across the three-objective DTLZ functions. For DTLZ2, 2P-HAMOEA achieved a GD of 0.013 ± 0.001 and a $\Delta_{p}$ of 0.276 ± 0.001, both of which were the lowest among the six algorithms. Its HV was 0.742 ± 0.001, which was higher than those of NSGA-II and SPEA2 but lower than those of NSGA-III and CTAEA. Its IGD was 0.053 ± 0.001 and was better only than that of NSGA-II according to this metric. For DTLZ1 and DTLZ7, 2P-HAMOEA showed comparatively favorable performance for selected measures of convergence, solution-set distribution, or runtime but did not outperform the comparator algorithms across all metrics.

Taken together, the benchmark-function results indicated that the relative performance of 2P-HAMOEA depended on the number of objectives, the geometry of the Pareto front, and the evaluation metric considered. Its advantages were confined to runtime, convergence, or solution-set distribution for particular problems. The results did not demonstrate consistent superiority over the comparator algorithms across all benchmark functions and evaluation metrics.

#### 2.3.2. Algorithm Comparison in the Application-Specific Multi-Endpoint Optimization Task

In the application-specific multi-endpoint optimization task, the six algorithms differed significantly in IGD, GD, and HV. The Friedman test yielded $\chi^{2}$ values of 141.791, 135.829, and 112.648, respectively, with all p<0.001.

The IGD of 2P-HAMOEA was 0.753 ± 0.039. Wilcoxon signed-rank tests showed that this value was lower than that of MOEA/D but higher than those of NSGA-II, SPEA2, NSGA-III, and CTAEA; all pairwise differences were statistically significant. Thus, on the basis of IGD, 2P-HAMOEA outperformed only MOEA/D.

The GD of 2P-HAMOEA was 0.092 ± 0.012, significantly lower than those of NSGA-II, SPEA2, NSGA-III, and CTAEA. Its GD did not differ significantly from that of MOEA/D, which was 0.087 ± 0.038 (p=0.119). Accordingly, 2P-HAMOEA outperformed four comparator algorithms in terms of GD but did not demonstrate superiority over MOEA/D.

The HV of 2P-HAMOEA was 163.821 ± 2.507, significantly lower than those of NSGA-II, NSGA-III, and CTAEA. Its HV did not differ significantly from those of SPEA2 or MOEA/D.

Across the 30 independent runs, 2P-HAMOEA generated an average of 200.100 ± 0.403 nondominated solutions and required 255.573 ± 30.081 s. The number of nondominated solutions was numerically similar to those obtained using NSGA-II and SPEA2. Its runtime was numerically lower than that of MOEA/D but higher than those of the other four algorithms. The number of nondominated solutions and runtime were compared descriptively only. The core performance metrics are summarized in Table 3.

Considering the three core solution-quality metrics together, 2P-HAMOEA achieved a relatively low GD in the application-specific task, but its IGD and HV were not superior to those of most comparator algorithms. Its runtime was also numerically higher than those of all algorithms except MOEA/D. These findings do not support characterizing 2P-HAMOEA as comprehensively superior to conventional multi-objective optimization algorithms.

#### 2.3.3. Ablation Study Results

For the full algorithm (FULL), the IGD, GD, HV, and SP were 0.13 ± 0.01, 0.05 ± 0.01, 36.75 ± 9.00, and 0.09 ± 0.01, respectively. Among the five algorithm settings, FULL had the numerically lowest IGD and highest HV. FULL, NO_LOCAL, and FIXED_LOCAL all round to a GD of 0.05 ± 0.01 at two-decimal precision; their underlying GD means (standard deviations) were 0.046726 (0.009237), 0.046777 (0.009272), and 0.046758 (0.009287), respectively. Based on the underlying unrounded values, NO_MSI had a slightly lower SP than FULL; both are displayed as 0.09 ± 0.01 at the reporting precision used in Table 4.

Removing the SOS-derived cooperative-search operators increased the IGD from 0.13 ± 0.01 for FULL to 0.15 ± 0.01 for NO_CSO and reduced the HV from 36.75 ± 9.00 to 35.40 ± 4.71. The underlying unrounded SP mean also increased; both SP results are displayed as 0.09 ± 0.01 after rounding to two decimal places. Differences between FULL and NO_CSO in all three metrics were statistically significant (all *p* < 0.05). The GD also increased from 0.05 ± 0.01 to 0.06 ± 0.01, although GD was not subjected to between-group statistical testing.

When local refinement was removed, the HV decreased to 36.43 ± 9.02 for NO_LOCAL and was significantly lower than that of FULL (p=0.018). Differences in IGD and SP between FULL and NO_LOCAL were not statistically significant. NO_LOCAL required 79.34 ± 3.73 s, numerically less than the 193.18 ± 11.53 s required by FULL; runtime was not tested statistically.

No statistically significant differences in IGD, HV, or SP were observed between NO_MSI and FULL. FIXED_LOCAL likewise did not differ significantly from FULL in any of these three metrics. Thus, under the current experimental settings, removing the SOS-derived cooperative-search operators was associated with statistically significant unfavorable changes in IGD, HV, and SP. Removal of local refinement significantly reduced HV. In contrast, no statistically significant changes in these metrics were detected after removal of multi-source initialization or replacement of adaptive local-operator scheduling with fixed selection probabilities.

**Figure 3:**
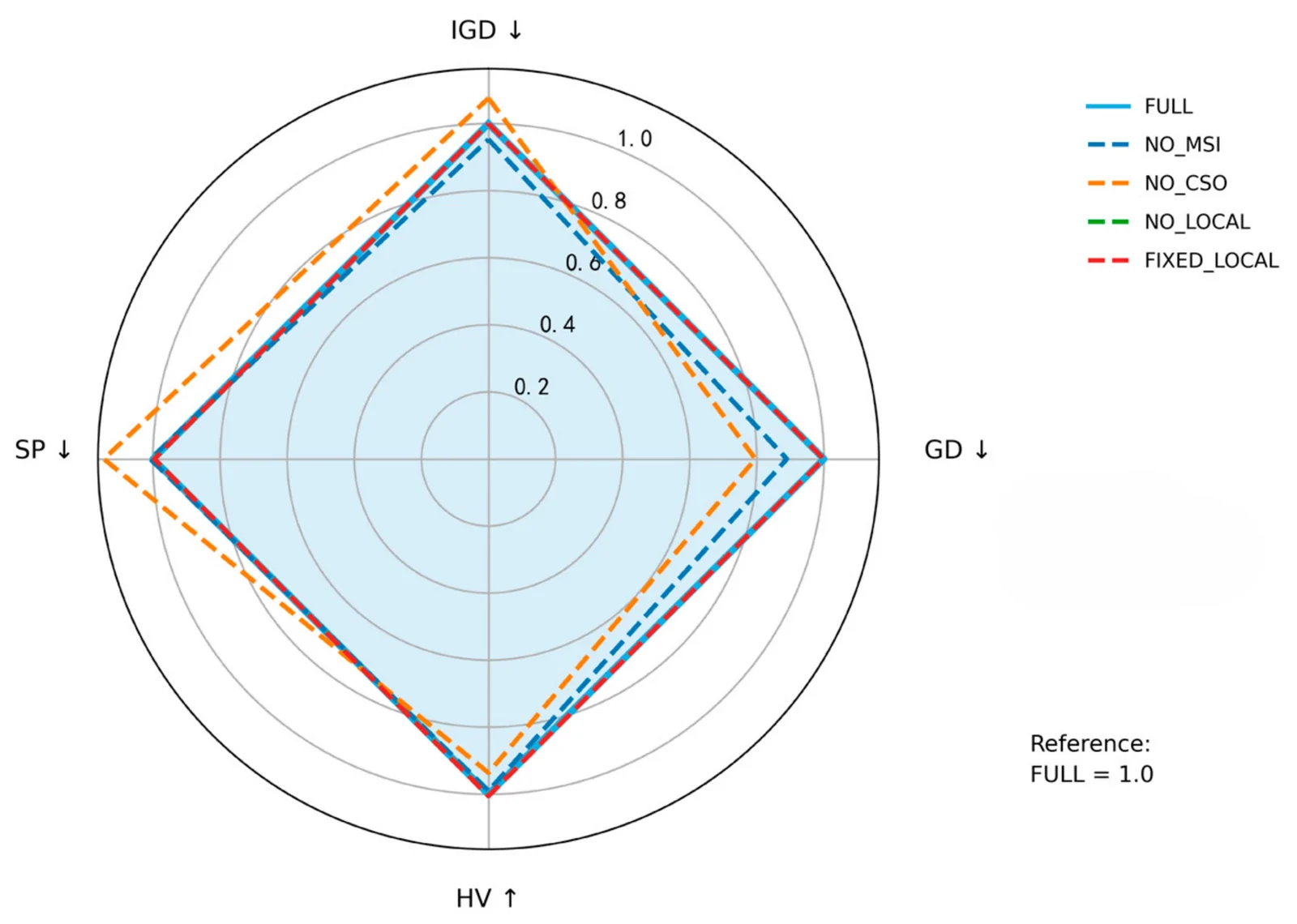
Relative solution-quality metric profiles of the full 2P-HAMOEA and its ablation variants. FULL denotes the complete algorithm, and the remaining abbreviations are defined in Table 4. The radar chart is intended to compare relative numerical metric profiles and does not indicate the statistical significance of differences between algorithm settings. The arrows indicate the preferred direction of the original metrics: lower IGD, GD, and SP values and higher HV values are preferable. The green dashed NO_LOCAL profile overlaps with other profiles and is therefore difficult to distinguish separately. Numerical comparisons are provided in Table 4.

The complete ablation results are presented in Table 4, and the relative metric profiles are shown in Figure 3.

### 2.4. Relationships Between Bioactivity and ADMET-Related Endpoints and Multi-Objective Optimization Results

#### 2.4.1. Relationships Between Bioactivity and ADMET-Related Endpoints

**Figure 4:**
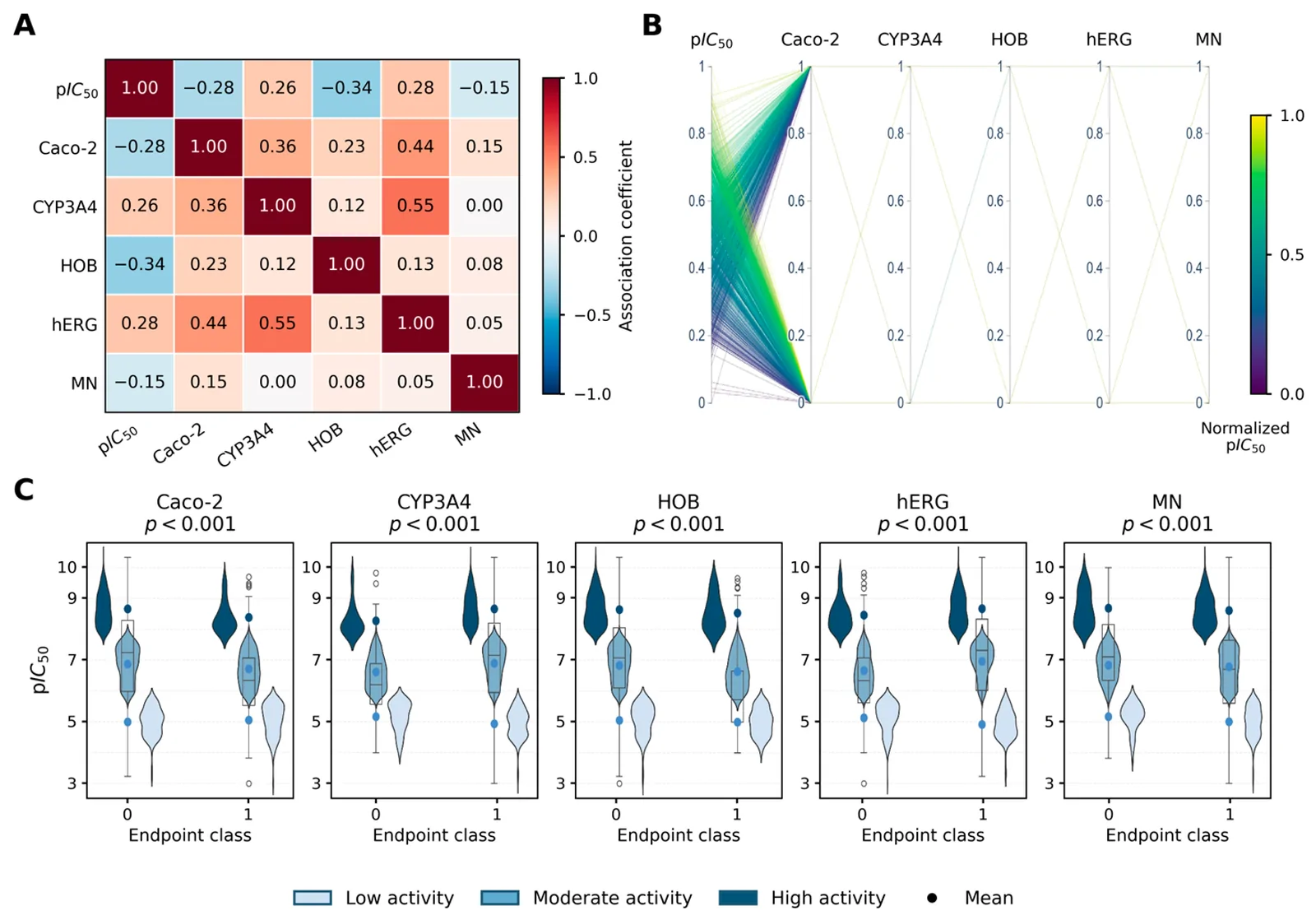
Statistical relationships and distributional patterns between p*IC*_50_ and the ADMET-related endpoints. (**A**) Statistical relationship matrix for the six endpoints in the training set. Point-biserial correlation coefficients were used to assess associations between p*IC*_50_ and the binary endpoints, whereas Cramér’s V was used to assess associations between pairs of binary endpoints. (**B**) Multi-endpoint parallel-coordinates plot based on all 1875 retained samples. Endpoint values were normalized for visualization, and the bioactivity-group cutoffs were derived from the training set. (**C**) Violin plots showing the distributions of p*IC*_50_ across the categories of the five ADMET-related endpoints. Between-group comparisons were performed using the Mann–Whitney U test. Coding throughout the figure is as follows: Caco-2 and HOB, 1 = relatively favorable property; CYP3A4, 1 = metabolized by CYP3A4; hERG and MN, 1 = presence of the corresponding toxicity risk. These analyses describe statistical associations and distributional differences and do not support causal inference.

In the training set, the absolute point-biserial correlation coefficients between p*IC*_50_ and the five ADMET-related endpoints were all below 0.35, indicating generally weak linear associations. The largest negative coefficient was observed between p*IC*_50_ and HOB (r=−0.34), whereas the negative association between p*IC*_50_ and MN was weaker (r=−0.15). Among the five binary endpoints, CYP3A4 and hERG showed a moderate association, with a Cramér’s V of 0.55. Cramér’s V was below 0.45 for all other pairs of binary endpoints (Figure 4A).

The 25th and 75th percentiles of p*IC*_50_ in the training set were 5.77 and 7.83, respectively, and were used to define the low-, intermediate-, and high-bioactivity groups. When these cutoffs were applied to all 1875 retained samples, the parallel-coordinates plot showed different multi-endpoint distribution patterns across the three groups. Samples in the high-bioactivity group were more frequently classified as CYP3A4 = 1 and hERG = 1, whereas samples in the low-bioactivity group were more frequently classified as Caco-2 = 1, HOB = 1, and MN = 1 (Figure 4B).

Because hERG = 1 and MN = 1 indicated predicted cardiotoxicity and genotoxicity, respectively, these distributional patterns did not indicate that any one bioactivity group had a uniformly more favorable ADMET profile. In addition, CYP3A4 = 1 indicated only that a compound was predicted to be metabolized by CYP3A4 and should not be interpreted as universally favorable. Rather, the findings showed that the statistical relationships between bioactivity and the individual ADMET-related endpoints differed in direction.

Mann–Whitney U tests showed that the distributions of p*IC*_50_ differed significantly between samples labeled 0 and 1 for each of the five ADMET-related endpoints (all p<0.001). p*IC*_50_ values tended to be lower in the Caco-2 = 1, HOB = 1, and MN = 1 groups and higher in the CYP3A4 = 1 and hERG = 1 groups (Figure 4C), consistent with the distributional patterns observed in the parallel-coordinates plot. These results describe statistical associations and distributional differences only and do not support causal inference. 

#### 2.4.2. Multi-Objective Optimization Results

Applying the p*IC*_50_ ≥ 7.83 requirement together with the prespecified target states for at least three of the five ADMET-related endpoints, Pareto-nondominance and solution-diversity filtering yielded 155 feasible Pareto solutions. The prespecified target states were Caco-2 = 1, CYP3A4 = 1, HOB = 1, hERG = 0, and MN = 0. The predicted p*IC*_50_ values of the feasible solutions were concentrated primarily between approximately 7.8 and 8.6.

Each feasible Pareto solution comprised values for the 24 key molecular descriptors, a predicted p*IC*_50_ value, and predicted classes for the five ADMET-related endpoints. The molecular descriptor values were inverse-transformed from the normalized values to their original scales. Because the solutions differed in their combined performance across the six endpoints, all 155 feasible Pareto solutions were then evaluated and ranked using objective weighting, multi-criteria decision-making, reliability-based fusion, and performance-uncertainty adjustment.

These feasible Pareto solutions were computational optimization results generated jointly from combinations of molecular descriptor values and endpoint-specific prediction models. They represented feasible solutions in molecular descriptor space under the current data, prediction models, optimization constraints, and filtering rules. They did not correspond to real compounds for which explicit molecular structures had been reconstructed, chemical synthesis had been performed, or experimental validation had been completed.

### 2.5. Comprehensive Evaluation of Feasible Pareto Solutions and Value Intervals for Key Molecular Descriptors

#### 2.5.1. Endpoint Weights and Composite Ranking Results

**Figure 5:**
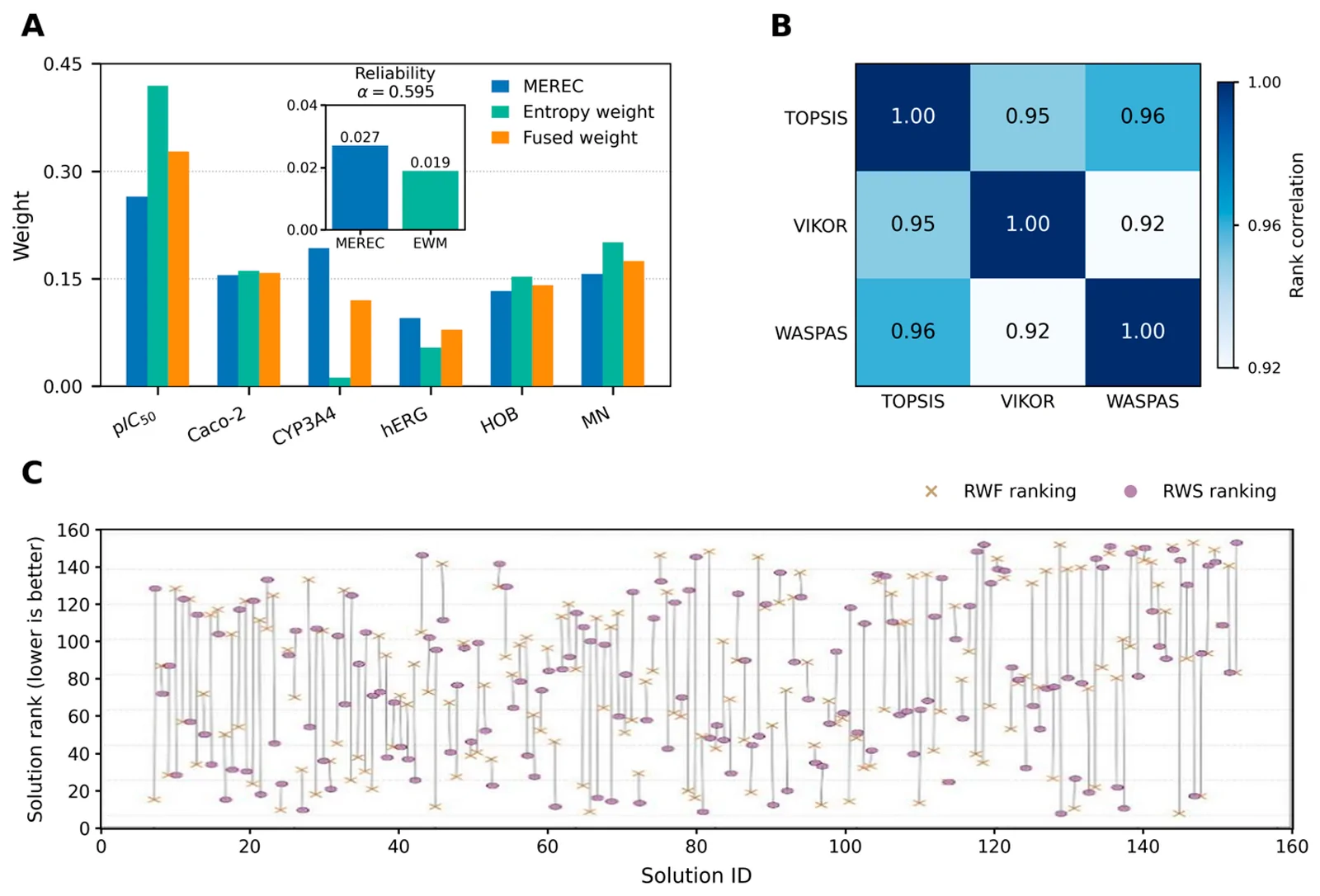
Multi-endpoint weights and composite rankings of the feasible Pareto solutions. (**A**) MEREC weights, EWM weights, and the corresponding fused weights calculated from the 155 feasible Pareto solutions. (**B**) Agreement among the rankings generated by TOPSIS, VIKOR, and WASPAS. (**C**) Comparison of the reliability-based weighted fusion (RWF) and robust weighted score (RWS) rankings after application of the performance uncertainty index–based stability adjustment. Lower numerical rank values indicate higher positions in the composite ranking. The endpoint weights apply only to the current feasible solution set and should not be interpreted as measures of absolute pharmacological importance.

MEREC and EWM produced different weight distributions across the six endpoints, with the most pronounced differences observed for p*IC*_50_ and CYP3A4. The MEREC and EWM weights for p*IC*_50_ were 0.265 and 0.419, respectively, whereas the corresponding weights for CYP3A4 were 0.193 and 0.012. After adaptive reliability-based fusion, p*IC*_50_ received the highest fused weight, at 0.328. The remaining endpoints, in descending order of fused weight, were MN, Caco-2, HOB, CYP3A4, and hERG, with weights of 0.175, 0.158, 0.141, 0.120, and 0.079, respectively (Table 5; Figure 5A).

The estimated reliabilities of MEREC and EWM were 0.027 and 0.019, respectively, and the adaptive fusion coefficient was 0.595. The resulting relative contributions of the two weighting methods to the fused weights were therefore 59.5% and 40.5%, respectively. These weights reflected the relative contributions of the endpoints to the comprehensive evaluation within the current set of 155 feasible Pareto solutions and under the specified weighting rules. They should not be interpreted as measures of the absolute importance of the endpoints in pharmacology or drug development.

The estimated reliabilities of the TOPSIS, VIKOR, and WASPAS rankings were approximately 0.002, 0.022, and 0.007, respectively, with VIKOR showing the highest relative reliability among the three methods. The overall ranking patterns generated by the three methods were broadly consistent, and all pairwise rank correlations exceeded 0.90. The rank correlations were 0.96 between TOPSIS and WASPAS, 0.95 between TOPSIS and VIKOR, and 0.92 between WASPAS and VIKOR (Figure 5B). Thus, the three methods produced similar overall assessments of the relative performance of the feasible solutions, although the rank assigned to an individual solution was not necessarily identical across methods.

The ranking obtained using reliability-based weighted fusion was also broadly consistent with those generated by the three individual multi-criteria decision-making methods, although the relative positions of some solutions changed. After the performance uncertainty index was incorporated, the overall structures of the RWS and RWF rankings remained similar. Most solutions showed only small changes in rank, whereas a smaller number underwent more noticeable changes in relative position (Figure 5C). This pattern was consistent with the design of the PUI adjustment, which imposed a greater score reduction on solutions with higher cross-endpoint dispersion. Nevertheless, the adjustment did not materially alter the overall ranking structure of the complete set of 155 feasible Pareto solutions.

#### 2.5.2. Value Intervals for Key Molecular Descriptors in the High-Scoring Subset of Feasible Pareto Solutions

After all 155 feasible Pareto solutions had been ranked in descending order of RWS, the top 20% were selected as the high-scoring subset. This subset comprised the 31 highest-ranked feasible Pareto solutions. Core, robust, and extended intervals were calculated for the 24 key molecular descriptors within this subset. All descriptor values were reported on their original scales after inverse transformation.

**Figure 6:**
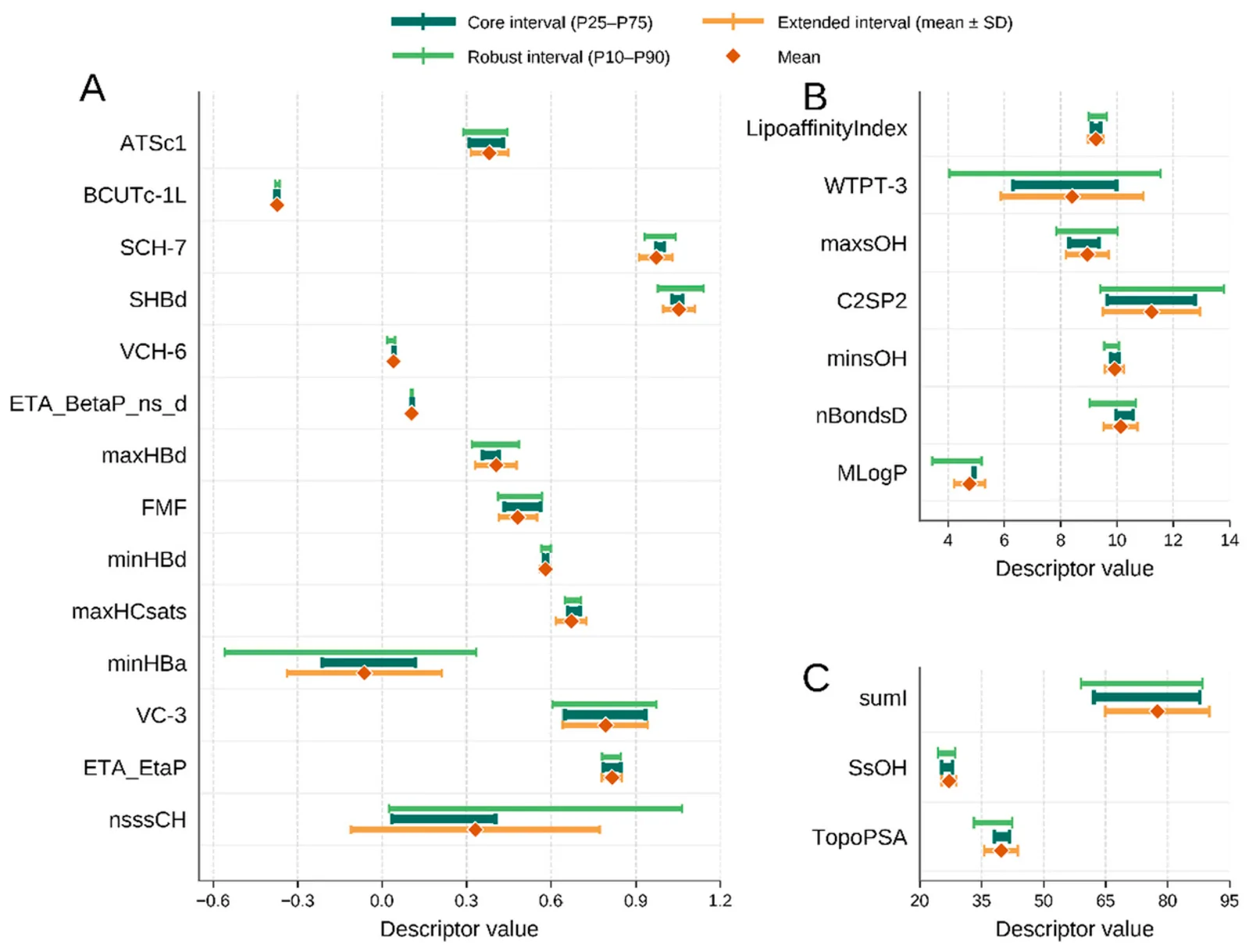
Value intervals for the key molecular descriptors in the high-scoring subset of feasible Pareto solutions. The figure presents the core intervals (P25–P75), robust intervals (P10–P90), and extended intervals (mean ± standard deviation) of the 24 key molecular descriptors among the top 20% of the 155 feasible Pareto solutions ranked by RWS (*n* = 31). The descriptors are divided among panels (**A**–**C**) according to their numerical scales to improve visualization. All values are presented on the original descriptor scales after inverse transformation. Because the three interval types are based on different statistical measures, the extended intervals need not be fully nested within the percentile-based intervals. Absolute interval widths should not be compared directly across descriptors, and the intervals should not be interpreted as experimentally validated structural thresholds.

The locations and relative extents of the intervals varied among descriptors on their respective scales (Figure 6). Because the descriptors differed in their computational definitions, units, and numerical scales, their absolute interval widths were not compared directly across descriptors. Instead, the core, robust, and extended intervals were compared within each descriptor.

For several descriptors, the three interval types were relatively similar. For ATSc1, the core, robust, and extended intervals were 0.309–0.430, 0.288–0.444, and 0.315–0.448, respectively. The corresponding intervals were −0.379 to −0.367, −0.380 to −0.363, and −0.381 to −0.364 for BCUTc-1L; 0.784–0.846, 0.780–0.847, and 0.779–0.850 for ETA_EtaP; and 0.574–0.588, 0.566–0.600, and 0.561–0.598 for minHBd. These descriptors therefore showed relatively concentrated values within the high-scoring subset.

For other descriptors, the robust intervals were noticeably wider than the corresponding core intervals. For MLogP, the core, robust, and extended intervals were 4.889–4.968, 3.446–5.197, and 4.218–5.315, respectively. The corresponding intervals were 6.315–9.977, 4.050–11.553, and 5.877–10.940 for WTPT-3; 9.652–12.774, 9.398–13.787, and 9.505–12.944 for C2SP2; and 0.648–0.934, 0.606–0.974, and 0.642–0.943 for VC-3. These patterns indicated that the degree of concentration and the spread of descriptor values varied within the high-scoring subset.

The three interval types characterized different aspects of the descriptor distributions among the 31 highest-ranked feasible Pareto solutions. The core interval represented the range containing the middle 50% of the high-scoring subset, whereas the robust interval covered approximately 80% of the subset. The extended interval was defined using the mean and standard deviation and reflected the central tendency and dispersion of each descriptor within the subset. Because the three interval types were based on different statistical measures, the extended interval was not required to be fully nested within either percentile-based interval.

The intervals were derived from the statistical distributions of the 31 highest-ranked feasible Pareto solutions obtained under the current data, prediction models, optimization constraints, weighting methods, and ranking rules. They describe only the numerical characteristics of the key molecular descriptors within the high-scoring subset. The intervals should not be interpreted as experimentally validated recommended ranges, deterministic structural thresholds, or direct rules for adding, removing, or substituting specific molecular functional groups. No model-specific feature-importance or SHAP analysis was used to derive these intervals; they are distributional summaries rather than model-mechanism explanations.

In the subset-threshold sensitivity analysis, the median top 10% versus top 30% interval-overlap indices across the 24 descriptors were 0.839 for the core intervals, 0.889 for the robust intervals, and 0.843 for the extended intervals (Table S3). Sensitivity was not uniform across descriptors: the robust interval for MLogP and the core and robust intervals for VCH-6 changed appreciably, while the core intervals for SCH-7 and SsOH were also more threshold dependent. Thus, the top 20% intervals described broadly similar central distributions for many descriptors, but they should not be regarded as invariant to the high-scoring-subset threshold.

## 3. Discussion

This study presents an integrated analytical workflow for evaluating the bioactivity and ADMET-related properties of candidate ERα antagonist compounds. The workflow combined key molecular descriptor selection, multi-endpoint property prediction, multi-objective optimization in molecular descriptor space, and comprehensive evaluation of feasible Pareto solutions. A two-stage cross-method and cross-endpoint screening procedure identified 24 key molecular descriptors, which were then used to develop separate final models for p*IC*_50_ and the five ADMET-related endpoints. The selected models were then incorporated into 2P-HAMOEA, which generated 155 feasible Pareto solutions that satisfied the prespecified criteria. All 155 solutions were evaluated and ranked through objective weighting, multi-criteria decision-making, reliability-based fusion, and performance-uncertainty adjustment. The top 20% according to RWS, comprising the 31 highest-ranked feasible Pareto solutions, were subsequently used to derive statistical value intervals for the key molecular descriptors. Together, these steps provide a quantitative basis for comparing alternative solutions in molecular descriptor space.

High-dimensional molecular descriptor data often contain low-information features and strongly correlated variables, which can increase model complexity and compromise prediction stability [20]. Feature-selection methods also differ in their evaluation criteria, and the structural information relevant to one endpoint may not be equally informative for another [21,22]. RF, AE, SC, SVM-RFE, and MIC were therefore used to assess candidate descriptors from complementary perspectives, including predictive contribution, data reconstruction, clustering structure, recursive feature elimination, and variable association. Cross-method and cross-endpoint comparison identified 24 descriptors that were selected for at least five endpoints. The aim was not to obtain the smallest possible feature subset for each endpoint but to define a common set of decision variables for all six prediction models and the subsequent optimization. Selection across methods and endpoints indicates statistical consistency under the current data and screening rules, but it does not establish causal effects or specific molecular mechanisms. The common set made all six endpoint-specific prediction functions compatible with one optimization space; it was not intended to be the most parsimonious or biologically optimal subset for every endpoint. In the controlled p*IC*_50_ comparison, the current 24 descriptors and the Dong et al. 20-descriptor set [23] produced nearly identical *R*^2^ values under the same stacking architecture, and the paired confidence interval included zero. The result does not support attributing the present *R*^2^ primarily to the larger proportion of topological or fragment descriptors, nor does it show that either descriptor set is generally superior. No direct comparison was conducted between the shared descriptor set and separately optimized endpoint-specific subsets; therefore, some endpoint-specific noise or loss of interpretability cannot be excluded.

The final selected model differed across endpoints, indicating that the relationships between molecular descriptors and individual properties followed different predictive patterns. Candidate and ensemble models were compared using the original equal-importance, multi-metric radar-area framework. For p*IC*_50_, the three-model stacking ensemble had the largest training-set ensemble radar area (8.081), the largest internal-holdout radar area (8.820), and the best values of all four reported holdout metrics among the compared candidates. For HOB, GB + TPE had the largest internal-holdout radar area (5.540) and the highest AUC (0.880), whereas XGBoost + TPE + CS showed higher recall and F_1_. The final selections were based on the multi-metric comparisons summarized in Supplementary Tables S5 and S6.

The two-phase structure of 2P-HAMOEA combined cooperative global search with adaptive local refinement. Its relative performance in the benchmark experiments depended on the number of objectives, Pareto-front geometry, and the evaluation metric considered. Runtime was comparatively short for the ZDT functions, although no consistent advantage was observed in HV or IGD. For selected DTLZ functions, GD or the solution-distribution metric was numerically favorable. A similar trade-off was observed in the application-specific task: GD was significantly lower than that of four comparator algorithms, but IGD and HV were not superior to those of most comparators, and runtime was numerically higher than that of every algorithm except MOEA/D. Because GD, IGD, and HV evaluate different aspects of optimization performance—the distance from the obtained solutions to the reference front, coverage of the reference front, and objective-space coverage, respectively [15]—no single metric provides a complete assessment. Overall, the findings indicate favorable performance for selected aspects of Pareto-front approximation but do not support characterizing 2P-HAMOEA as comprehensively superior to conventional multi-objective optimization algorithms. The hybrid structure was retained for a task-specific reason: the 24-dimensional decision space was evaluated through one continuous bioactivity prediction and five classification-based outputs under multiple constraints, creating a heterogeneous search landscape in which objective-space coverage, complementary decision-space exploration, and local refinement could play different roles. This rationale does not establish universal empirical superiority; standard algorithms remain reasonable alternatives when simplicity or runtime is the primary consideration.

The ablation findings further clarified the contributions of individual algorithm components. Removing the SOS-derived cooperative-search operators increased IGD and SP and reduced HV, with all three changes reaching statistical significance under the current experimental settings. At the operator level, NO_CSO removed the partner-based mutualism and commensalism moves and the stochastic coordinate replacement used by parasitism, so global exploration before local refinement depended on the NSGA-III reference-direction component alone. The end-of-run metric changes are consistent with a loss of complementary decision-space exploration. However, the ablation experiment recorded end-of-run metrics rather than generation-wise population histories and therefore does not establish a specific iteration-by-iteration change in the search trajectory. Removing local refinement significantly reduced HV but did not produce statistically significant differences in IGD or SP and substantially reduced runtime numerically. No statistically significant differences in IGD, HV, or SP were detected after removal of multi-source initialization or replacement of adaptive local-operator scheduling with fixed selection probabilities. These results are limited to the current data, parameter settings, and evaluation metrics.

The multi-endpoint relationship analysis showed generally weak point-biserial correlations between p*IC*_50_ and the five ADMET-related endpoints, although the distributions of p*IC*_50_ differed significantly between samples labeled 0 and 1 for every ADMET-related endpoint. The two findings are compatible: point-biserial correlation coefficients quantify the overall linear association between a continuous and a binary variable, whereas the Mann–Whitney U test compares the distributions of two groups. Higher p*IC*_50_ values occurred more frequently in the CYP3A4 = 1 and hERG = 1 groups, whereas p*IC*_50_ values tended to be lower in the Caco-2 = 1, HOB = 1, and MN = 1 groups. Because hERG = 1 and MN = 1 indicated predicted cardiotoxicity and genotoxicity, respectively, higher ERα inhibitory activity was not associated with consistent changes toward more favorable states across all ADMET-related properties. Improving bioactivity alone may therefore not ensure simultaneous improvement in the other properties, supporting the use of multi-objective optimization to retain nondominated solutions with different endpoint profiles. These analyses describe statistical associations and distributional differences only and do not support causal inference.

During multi-objective optimization, Caco-2 = 1, CYP3A4 = 1, HOB = 1, hERG = 0, and MN = 0 were specified as the target states. A total of 155 feasible Pareto solutions were obtained under the requirements of p*IC*_50_ ≥ 7.83 and achievement of the target states for at least three ADMET-related endpoints. CYP3A4 = 1 indicated only that a compound was predicted to be metabolized by this enzyme and was selected as a target state according to the original class coding. This coding should not be interpreted as universally favorable across all drug-development contexts. Pareto nondominance retained multiple solutions with different endpoint profiles but could not directly provide a unique priority order. MEREC and EWM were therefore used to calculate and fuse the endpoint weights, after which TOPSIS, VIKOR, and WASPAS were applied to evaluate the solutions [24,25,26,27]. The rank correlations among the three methods were all above 0.90, indicating broadly similar assessments of the overall ranking structure, although the exact ranks of individual solutions were not identical.

p*IC*_50_ received the highest fused endpoint weight. However, this result was derived from the endpoint distributions and criterion-removal effects within the current set of 155 feasible Pareto solutions. It reflects only the relative information contribution under the specified solution set and evaluation rules and does not indicate that p*IC*_50_ has a fixed or universally applicable highest priority in drug development. RWF integrated the evaluation results from the three multi-criteria decision-making methods, whereas RWS incorporated a PUI-based adjustment into RWF so that solutions with greater cross-endpoint dispersion received larger score reductions. In this context, PUI describes dispersion among endpoint values within an individual solution and does not represent prediction error, a confidence interval, or variability across repeated experiments. The RWS ranking was intended primarily for relative comparison within the current feasible solution set and should not be equated directly with the actual development priority of candidate compounds.

The core, robust, and extended intervals calculated from the 31 highest-ranked feasible Pareto solutions characterized the statistical distributions of the 24 key molecular descriptors within the high-scoring region of the current feasible solution space. The three intervals were relatively similar for ATSc1, BCUTc-1L, ETA_EtaP, and minHBd, indicating comparatively concentrated values within the high-scoring subset. In contrast, the robust intervals for MLogP, WTPT-3, C2SP2, and VC-3 were appreciably wider than their core intervals, showing that these descriptors retained greater variation among the highest-ranked solutions. Because the descriptors differed in their computational definitions, units, and numerical scales, interval widths should be compared within, rather than across, descriptors. They should not be used to infer directly how strongly different descriptors were constrained. Moreover, these numerical intervals cannot be translated directly into rules for adding, removing, or substituting specific functional groups.

The 24 descriptors capture complementary dimensions relevant to compound comparison: ATSc1, BCUTc-1L, sumI, and the ETA indices represent charge-distribution or electrotopological information; TopoPSA, the hydrogen-bond E-state descriptors, and the hydroxyl-group E-state descriptors represent polarity and local hydrogen-bonding environments; MLogP and LipoaffinityIndex represent lipophilicity; and SCH-7, WTPT-3, VCH-6, VC-3, FMF, nBondsD, C2SP2, nsssCH, and maxHCsats represent molecular connectivity, framework organization, unsaturation, or local atom environments. The relatively concentrated values of ATSc1, BCUTc-1L, ETA_EtaP, and minHBd indicate that the highest-ranked numerical profiles occupied comparatively restricted regions for these properties, whereas the wider within-descriptor distributions of MLogP, WTPT-3, C2SP2, and VC-3 indicate that no single narrow numerical region was identified for those properties. These observations provide descriptor-level context only; they do not establish causal effects, receptor-binding mechanisms, or substituent-level structure–activity relationships. No model-specific feature-importance or SHAP analysis was performed for the heterogeneous final ADMET models. Consequently, cross-method selection frequency and interval concentration should not be interpreted as explanations of the internal prediction mechanism of each final model.

The main contribution of this work lies in integrating high-dimensional descriptor screening, endpoint-specific predictive modeling, Pareto solution search, and multi-criteria evaluation within a single analytical workflow. A shared descriptor set enabled all endpoint-specific models to operate in the same decision space. Pareto analysis retained trade-offs among bioactivity, pharmacokinetic properties, and safety, while weighting and composite ranking provided a structured basis for comparing alternative nondominated solutions. Returning the optimization results to the original descriptor scales further allowed the numerical distributions of key descriptors within the high-scoring subset to be described directly. The value of the framework therefore lies primarily in multi-endpoint statistical modeling, organization of computational solutions, and quantitative analysis of molecular descriptor ranges, rather than in the direct generation of experimentally validated compounds. It may provide a methodological reference for subsequent compound screening and structural investigation.

In practice, the framework can support staged computational triage after medicinal chemists or an external design method have proposed explicit candidate structures. Such structures may arise from an analogue series, structure-based design, a virtual-screening or commercial library, or an independently developed molecular-generation method. Once a harmonized, compatible implementation of the 24 descriptor calculations is available, candidates should first be checked against the descriptor-space applicability domain. The p*IC*_50_ and five ADMET predictions can then be reviewed separately; candidates that are dominated across objectives can be deprioritized, while representatives of potency-favoring, exposure-favoring, safety-favoring, and balanced Pareto profiles can be advanced to receptor-based evaluation, synthetic-feasibility review, and targeted assays. The workflow can therefore help determine which already-proposed structures to advance and which endpoint should guide further optimization, but it does not itself create a scaffold. The 155 feasible solutions and descriptor intervals remain within-study computational reference profiles, not drug candidates, experimentally validated design thresholds, or substitutes for biological testing.

Several limitations should be considered. First, the analysis was based on a single dataset. Although tenfold cross-validation and a maximum-dissimilarity internal holdout were used for model comparison and evaluation, no independently sourced dataset was available for external validation. The joint t-SNE plot and leverage diagnostic showed that 86.1% of the holdout was within the training-set warning leverage, but these checks operate only in the selected 24-descriptor space. Maximum dissimilarity was neither a molecular-scaffold split nor an external chemotype challenge, and compounds above the warning leverage indicate peripheral descriptor combinations for which predictions require particular caution. The present results therefore do not establish performance for structurally novel chemotypes, and prospective use should be restricted to explicitly defined compounds within a verified descriptor-space AD until scaffold-based and external evaluations are available. The internal holdout also contributed to final model selection; bootstrap intervals quantify compound-level sampling variation but do not remove selection uncertainty. Some data-preprocessing and feature-selection procedures were performed using the complete dataset, and the corresponding internal estimates require confirmation using independent data. Second, the compounds were represented primarily using conventional molecular descriptors, with limited incorporation of three-dimensional conformational information, spatial electronic distributions, or receptor–ligand interactions. In addition, several parameters of 2P-HAMOEA were prespecified, and its performance under different problem sizes, numbers of objectives, and constraint structures remains to be evaluated.

Third, the low-variance cutoff, isolation-forest contamination rate, Spearman redundancy threshold, and five-of-six cross-endpoint consensus rule were prespecified procedural choices and were not subjected to a complete end-to-end sensitivity analysis. The targeted top 10–top 30% analysis examined the final subset-size threshold and found relatively high median interval overlap together with descriptor-specific sensitivity. The reported intervals therefore remain conditional on the current preprocessing, feature-selection, optimization, weighting, and ranking settings.

Fourth, the molecular descriptors were provided as precomputed variables, and the available source documentation did not report the software package or version used to generate them. This limits exact descriptor recalculation from new structures. In addition, no harmonized model-specific feature-importance or SHAP analysis was performed across the heterogeneous final ADMET models. Future work should document descriptor-generation provenance and apply a prespecified, comparable explanation framework with independent validation before attributing mechanistic meaning to individual descriptors.

Finally, the 155 feasible Pareto solutions are computational combinations of the 24 key molecular descriptors within specified ranges. They have not been reconstructed as compounds with explicitly defined molecular structures or established synthetic feasibility and have not undergone further computational or experimental validation. Future studies could incorporate larger multisource compound datasets together with molecular graphs and three-dimensional conformational information. Molecular structure generation, molecular docking, molecular dynamics simulations, and in vitro assessments of bioactivity, pharmacokinetics, and toxicity could then be used to evaluate the prediction models, feasible solutions, and descriptor intervals, thereby improving the generalizability and practical utility of the framework.

## 4. Materials and Methods

The computational analyses in this study were performed using Python version 3.14.0 and R version 4.5.2.

### 4.1. Data Source and Study Variables

This study used the dataset provided for Problem D, “Optimization Modeling of Candidate Anti-Breast-Cancer Drugs,” in the 18th China Postgraduate Mathematical Contest in Modeling. The dataset contained 1974 organic compounds. Each compound was represented by a simplified molecular-input line-entry system (SMILES) string and was accompanied by 729 molecular descriptors, bioactivity data, and five ADMET-related properties. Each compound was treated as one unit of analysis. Molecular descriptors served as candidate inputs for feature selection, multi-endpoint property prediction, and multi-objective optimization in molecular descriptor space. SMILES strings were used solely to encode and identify molecular structures and were not entered directly into the prediction models or the multi-objective optimization procedure. The 729 molecular descriptors were supplied as precomputed variables in the source dataset; the authors did not recalculate them from the SMILES strings. The source documentation available for this study did not identify the descriptor-calculation software or version. We therefore retained the original descriptor names and supplied numerical values without inferring an undocumented software package.

The inhibitory activity of each compound against estrogen receptor alpha (ERα) was characterized using the half-maximal inhibitory concentration (*IC*_50_) and its logarithmically transformed value, p*IC*_50_, as provided in the dataset. Under the conventional molar definition, p*IC*_50_ = −log_10_(*IC*_50_ [mol/L]). Lower *IC*_50_ values indicate stronger ERα inhibitory activity, whereas higher p*IC*_50_ values indicate greater activity. In this study, p*IC*_50_ was used as the continuous bioactivity endpoint for regression modeling.

The dataset also included five binary ADMET-related endpoints: Caco-2 cell permeability, metabolism by cytochrome P450 3A4 (CYP3A4), human ether-à-go-go-related gene (hERG)-associated cardiotoxicity, human oral bioavailability (HOB), and micronucleus (MN) genotoxicity. These endpoints represented cell permeability, whether a compound could be metabolized by CYP3A4, cardiotoxicity risk, oral bioavailability, and genotoxicity risk, respectively. Each endpoint was coded as 0 or 1. Together, p*IC*_50_ and the five ADMET-related endpoints constituted the six prediction endpoints examined in this study and were subsequently incorporated into the multi-objective optimization. The original label definitions for the five classification endpoints are presented in Table 6.

### 4.2. Data Preprocessing, Selection of Key Molecular Descriptors, and Dataset Partitioning

#### 4.2.1. Data Preprocessing

Molecular descriptors with variances below 1 × 10^−5^ were first removed as near-constant variables, after which the remaining descriptors were examined for missing values. Pairwise Spearman rank correlation coefficients were then calculated, with an absolute correlation coefficient of 0.95 used as the threshold for redundancy reduction. Within descriptor pairs or groups above this threshold, variables that were less correlated with the remaining descriptors were preferentially retained. This reduced redundancy while preserving relatively distinct information. At the preprocessing stage, the 0.95 cutoff was used to remove near-duplicate descriptors, not to require mutual independence among all retained variables. Correlations below this cutoff were not treated as an additional automatic exclusion criterion because final retention was determined by the subsequent cross-method and cross-endpoint screening procedure. The pairwise Spearman analysis was also repeated after final feature selection to characterize redundancy within the retained 24-descriptor set.

At the sample level, outliers were detected using isolation forest (IF), with the contamination rate prespecified at 5%. The 5% rate was a procedural choice used to flag a limited proportion of markedly atypical multivariate observations and was not selected by optimizing downstream model performance. IF constructs multiple isolation trees by randomly selecting descriptors and split points and quantifies the degree of abnormality of each sample according to its average path length across the trees. Samples farther from the overall distribution tend to be isolated by shorter path lengths. After the detected outliers were removed, min–max normalization was applied to scale the retained molecular descriptors to the range of 0–1 for subsequent descriptor selection and prediction model development.

#### 4.2.2. Multi-Method and Multi-Endpoint Selection of Key Molecular Descriptors

Candidate molecular descriptors were evaluated separately for p*IC*_50_, Caco-2, CYP3A4, hERG, HOB, and MN using random forest (RF), autoencoder (AE), spectral clustering (SC), support vector machine–recursive feature elimination (SVM-RFE), and the maximal information coefficient (MIC). These methods assessed descriptor information from complementary perspectives, including predictive contribution, data reconstruction, clustering structure, recursive feature elimination, and variable association.

Within a tenfold cross-validation framework, the mean importance value or mean rank of each descriptor across folds was calculated, together with the frequency with which the descriptor appeared among the top 60 candidates. Mean importance and selection frequency were normalized separately and then combined using a weighted integration procedure, with greater weight assigned to mean importance than to selection frequency. For each screening method and endpoint, the 60 descriptors with the highest composite scores were retained as the candidate feature set.

A two-stage cross-method and cross-endpoint screening procedure was then used. In the first stage, candidate feature sets generated by the five screening methods were compared within each endpoint, and descriptors repeatedly selected by multiple methods were identified. In the second stage, the endpoint-specific screening results were compared across all six endpoints, and descriptors identified for at least five endpoints were retained. The five-of-six rule was prespecified as a stringent cross-endpoint consensus criterion while permitting one endpoint-specific exception; it was not optimized against downstream holdout performance. The resulting key molecular descriptors served as a common set of input variables for the p*IC*_50_ regression model, the five ADMET classification models, and the subsequent multi-objective optimization in molecular descriptor space. This common set was not derived from the bioactivity model and then imposed on the ADMET models; rather, it was the cross-endpoint consensus set, and every retained descriptor met the criterion of selection for at least five of the six endpoints.

#### 4.2.3. Training-Holdout Split

Following data preprocessing and key molecular descriptor selection, one fixed maximum-dissimilarity partition was constructed from the shared 24-descriptor matrix and reused for the p*IC*_50_ regression task and all five ADMET classification tasks. The assignment was outcome-independent. The sequential procedure began with one initial compound. At each subsequent step, the remaining compound with the largest minimum Euclidean distance from those already assigned to the internal holdout was added, until the set contained 375 compounds. The other 1500 compounds formed the training set, giving a 4:1 ratio. The realized training and internal-holdout compound IDs and the associated model-ready descriptor matrix have been retained and are available from the corresponding author upon reasonable request; the assignment is identical across the six endpoints. The design was intended to broaden coverage of the shared descriptor space and make the internal comparison more demanding than an ordinary random split. Because it was derived from one source dataset and descriptor distances rather than molecular scaffolds, it was not an external or scaffold-based validation of structurally novel chemotypes.

To visualize the fixed assignment in one common coordinate system, a joint t-distributed stochastic neighbor embedding (t-SNE) was generated for all 1875 compounds from the 24 standardized descriptors; training and internal-holdout labels were applied only after the embedding. The settings were two components, perplexity = 30, 1000 iterations, random_state = 42, PCA initialization, and automatic learning-rate selection. The t-SNE analysis served only as a descriptive visualization and did not contribute to model fitting, hyperparameter optimization, model selection, or predictive performance evaluation.

#### 4.2.4. Descriptor-Space Applicability Domain

A leverage-based diagnostic was used to examine the applicability domain (AD) in the shared 24-descriptor space. Using the training design matrix *X* with an intercept, leverage was calculated as *h_i_* = *x_i_*^T^(*X*^T^*X*)^−1^*x_i_*. The warning threshold was h* = 3(*p* + 1)/*n* = 3 × 25/1500 = 0.050, where *p* = 24 descriptors and *n* = 1500 training compounds. Internal-holdout compounds with *h_i_* ≤ 0.050 were considered within the training-set descriptor-space AD; compounds above this warning leverage were flagged as peripheral. The same descriptor matrix and fixed assignment were used for all six endpoints, so one AD analysis applied to all six. This *X*-space diagnostic evaluates multivariate descriptor coverage only and does not establish scaffold, mechanistic, receptor-binding, or external chemical-space applicability.

### 4.3. Development and Evaluation of Prediction Models for Bioactivity and ADMET-Related Properties

#### 4.3.1. Candidate Prediction Models

Using the selected key molecular descriptors as predictors, separate regression and classification models were developed for p*IC*_50_ and the five ADMET-related endpoints, respectively. Thus, p*IC*_50_ and the ADMET outcomes were not combined as a single dependent variable, and no endpoint outcome was used as a predictor of another endpoint.

For the continuous p*IC*_50_ endpoint, the candidate regression algorithms included multilayer perceptron (MLP), K-nearest neighbors (KNN), support vector regression (SVR), random forest (RF), extreme gradient boosting (XGBoost), Oblivious Tree, and Gaussian process regression (GPR).

Caco-2, CYP3A4, hERG, HOB, and MN were modeled separately as binary endpoints. The candidate classification algorithms for each endpoint included support vector machine (SVM), logistic regression (LR), Light Gradient Boosting Machine (LightGBM), gradient boosting (GB), RF, XGBoost, MLP, and backpropagation neural network (BPNN). Modeling the five endpoints independently allowed the predictive performance of the candidate algorithms to be evaluated for each classification task.

#### 4.3.2. Hyperparameter Optimization, Cost-Sensitive Learning, and Ensemble Modeling

All candidate models were evaluated by tenfold cross-validation within the training set. A tree-structured Parzen estimator (TPE) was used to search the hyperparameter space, and the cross-validation performance of the TPE-optimized models was compared with that of the corresponding baseline models. Hyperparameter optimization was conducted entirely within the training set; the internal holdout set was not used for hyperparameter selection.

To account for class imbalance in the ADMET classification tasks, cost-sensitive learning (CS) was applied to selected classifiers. Class-specific costs were incorporated during model fitting or classification decision-making so that samples from the two classes could contribute more appropriately to model development. Depending on the algorithm, four model configurations were compared where applicable: the baseline model, the TPE-optimized model, the cost-sensitive model, and the model combining TPE optimization with cost-sensitive learning.

Three ensemble strategies were also evaluated: simple averaging, Softmax-weighted averaging, and two-layer stacking. In the simple averaging approach, predictions or class probabilities from the base learners were combined using an arithmetic mean. For Softmax-weighted averaging, base-learner weights were derived from their composite performance across multiple metrics in tenfold cross-validation within the training set and then normalized using the Softmax function. In the stacking approach, predictions from the first-layer base learners were passed to a second-layer meta-learner. Linear regression served as the meta-learner for the regression task, whereas logistic regression was used for the classification tasks. For hERG classification, the five thresholded constituent-model predictions were averaged and the result thresholded at 0.5, which is equivalent to majority voting.

For each endpoint, the three and five highest-ranked individual models, based on their composite cross-validation performance in the training set, were used to construct separate ensemble configurations. This design allowed both the ensemble strategy and the number of base learners to be compared.

#### 4.3.3. Model Evaluation and Final Model Selection

The performance of the p*IC*_50_ regression models was assessed using mean absolute error (MAE), median absolute error (MedAE), mean squared error (MSE), and the coefficient of determination (*R*^2^). Lower MAE, MedAE, and MSE values and a higher *R*^2^ indicated better predictive performance.

The ADMET classification models were evaluated using accuracy, precision, recall, the area under the receiver operating characteristic curve (AUC), and the F_1_ score. Tenfold cross-validation results from the training set were summarized as the mean ± standard deviation.

To compare candidate models across multiple metrics, all metrics were oriented so that higher values indicated better performance and then normalized. The metrics were assigned equal importance, and the enclosed radar area was used to summarize overall performance. Individual models were ranked using tenfold cross-validation results from the training set; the three and five highest-ranked models were then used to construct simple-average, Softmax-weighted, and stacking ensembles. The shortlisted individual models and ensemble configurations were compared in the internal holdout set using the same endpoint-appropriate metrics and radar-area summary. The original model-selection results are reproduced in Supplementary Tables S5 and S6.

For p*IC*_50_ and each of the five ADMET-related endpoints, the final model was selected using the original multi-metric comparison described above. Because the internal holdout set contributed to the final comparison among shortlisted candidates, its reported metrics are internal comparative estimates rather than external-validation performance. The selected endpoint-specific prediction functions were subsequently used in the multi-objective optimization conducted in molecular descriptor space.

#### 4.3.4. Controlled Descriptor-Set Comparison and Uncertainty Estimation

To examine whether the 24-descriptor set itself explained the lower *R*^2^ relative to a previous report, a controlled sensitivity comparison was conducted using the 20 p*IC*_50_ descriptors reported by Dong et al. [23]. The two sets shared five exactly named descriptors: BCUTc-1L, LipoaffinityIndex, MLogP, maxsOH, and minsOH. The existing MD-FIS compound assignments, three first-layer model families, fixed hyperparameters, preprocessing settings, stacking architecture, and internal holdout were kept the same for both descriptor sets, leaving the descriptor set as the main factor that differed. Paired differences in internal-holdout metrics were evaluated by 5000 paired bootstrap resamples. This was a separate sensitivity analysis and did not replace the formally reported final-model results.

Sampling uncertainty for the final internal-holdout metrics was also summarized by nonparametric bootstrap intervals. For p*IC*_50_, 5000 resamples of the 375 holdout compounds were used. For each final ADMET model, 10,000 resamples of the corresponding 375 compound-level labels and prediction scores were used. The 2.5th and 97.5th percentiles defined the 95% confidence interval. The intervals describe sampling variation within the present internal holdout sets; they do not capture model-selection uncertainty or generalizability to an external chemical series.

### 4.4. Multi-Objective Optimization and Algorithm Validation

#### 4.4.1. Two-Phase Hybrid Adaptive Multi-Objective Evolutionary Algorithm

The final selected models for p*IC*_50_ and the five ADMET-related endpoints were incorporated into the multi-objective optimization procedure, with the selected key molecular descriptors serving as decision variables. A two-phase hybrid adaptive multi-objective evolutionary algorithm (2P-HAMOEA) combined multi-source initialization, cooperative global search, adaptive local refinement, and nondominance- and diversity-based filtering.

The initial population was generated using a combination of random sampling, Tent chaotic mapping, and Lévy flight. Each decision variable was bounded by the observed univariate range of the corresponding molecular descriptor, ensuring that the generated descriptor values remained within the boundaries observed in the original dataset.

The first phase combined NSGA-III reference-direction guidance with SOS-derived cooperative-search operators. The NSGA-III component primarily maintained the distribution of nondominated solutions in objective space, whereas the mutualism, commensalism, and parasitism operators introduced interaction-based and stochastic perturbations in decision space. Candidate solutions produced during the cooperative global phase were subjected to nondominance filtering before local refinement. Here, “parallel” describes cooperation among complementary search components rather than concurrent hardware computation.

The second phase involved adaptive local refinement. Elite solutions were first selected from the nondominated solutions obtained during global search. A local-search strategy pool comprising differential evolution (DE), particle swarm optimization (PSO), and simulated annealing (SA) was then applied. The probability of selecting each operator was updated dynamically according to its historical search success rate, and new candidate solutions were generated within the neighborhoods of the elite solutions. A new solution replaced the original solution only if it Pareto-dominated the original, meaning that it was no worse for any endpoint and improved at least one endpoint. Otherwise, the original solution was retained.

**Figure 7:**
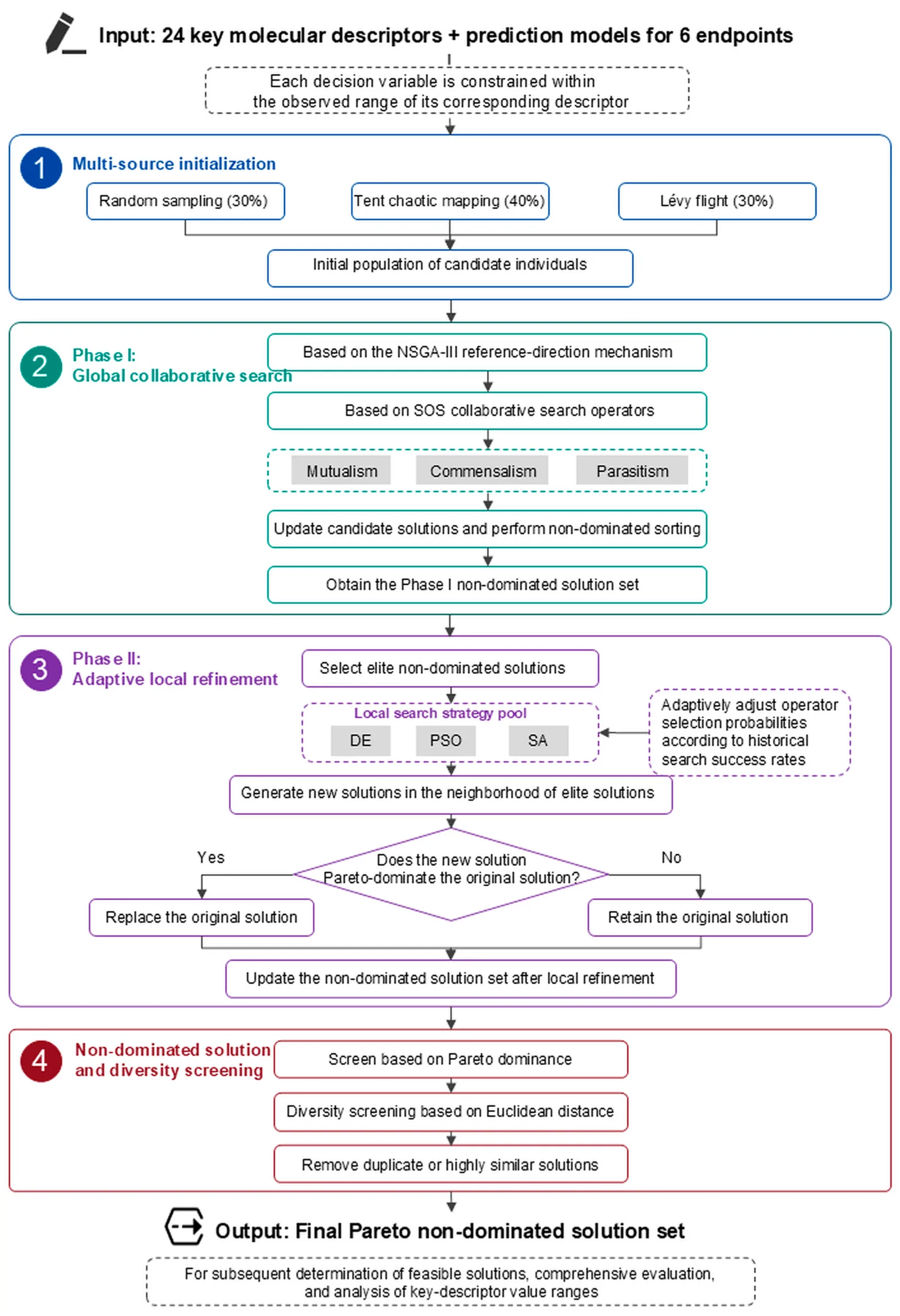
Workflow of the two-phase hybrid adaptive multi-objective evolutionary algorithm.

Following local refinement, a diversity filter based on the minimum Euclidean distance was applied to remove duplicate or highly similar solutions. This step reduced redundancy while preserving the distribution of the solution set in the decision space. The final Pareto-nondominated solution set was obtained after nondominance and diversity filtering. The overall 2P-HAMOEA workflow is shown in Figure 7.

#### 4.4.2. Optimization Objectives and Identification of Feasible Pareto Solutions

The key molecular descriptors served as decision variables, and the final endpoint-specific models generated predictions for p*IC*_50_, Caco-2, CYP3A4, hERG, HOB, and MN during multi-objective optimization. p*IC*_50_ was retained as a continuous prediction. Each ADMET classification model returned the predicted probability of class 1, and these class-1 probabilities were used as continuous optimization objectives with endpoint-specific directions. For hERG, the class-1 probabilities from the same five constituent learners used in the evaluation ensemble were averaged. The 0.5 cutoff was applied only when binary classes were needed to count target-state satisfaction in the feasibility criterion or to present results in tables.

Higher predicted p*IC*_50_ values were preferred. The class-1 probabilities for Caco-2, CYP3A4, and HOB were maximized, whereas the class-1 probabilities for hERG and MN were minimized. The corresponding binary target classes in the feasibility criterion were 1 for Caco-2, CYP3A4, and HOB and 0 for hERG and MN. These target states represented relatively favorable cell permeability, the ability to be metabolized by CYP3A4, relatively favorable oral bioavailability, and the absence of predicted cardiotoxicity and genotoxicity, respectively. CYP3A4 = 1 was treated as a target class solely under the optimization settings of this study and should not be regarded as universally favorable across all drug-development contexts.

The 75th percentile of p*IC*_50_ in the training set was used to define the high-bioactivity threshold. Accordingly, p*IC*_50_ ≥ 7.83 was specified as the high-bioactivity condition. Under the conventional definition p*IC*_50_ = −log_10_(*IC*_50_ [mol/L]), this value corresponds to an *IC*_50_ of approximately 14.8 nM. However, 7.83 was selected solely from the training-set distribution and was not treated as a validated pharmacological or clinical potency cutoff. Solutions were included in the feasible region if they satisfied this condition and achieved the prespecified target states for at least three of the five ADMET-related endpoints. Pareto-nondominance and solution-diversity filtering were then applied to obtain the feasible Pareto solutions used in the subsequent comprehensive evaluation and ranking. After all feasible solutions had been ranked using the robust weighted score, the highest-ranked subset was selected for descriptor-interval analysis, as described in Section 4.5.3.

The feasible Pareto solutions were generated jointly from combinations of molecular descriptor values and endpoint-specific prediction models. They represented computational optimization results in molecular descriptor space and did not correspond to real compounds for which explicit molecular structures had been reconstructed, chemical synthesis had been performed, or experimental validation had been completed.

#### 4.4.3. Benchmark-Function Evaluation and Algorithm Comparison

The ZDT1, ZDT2, and ZDT3 two-objective benchmark functions and the DTLZ1, DTLZ2, and DTLZ7 three-objective benchmark functions were used to evaluate 2P-HAMOEA across different Pareto-front structures. The comparator algorithms were the nondominated sorting genetic algorithm II (NSGA-II), strength Pareto evolutionary algorithm 2 (SPEA2), multi-objective evolutionary algorithm based on decomposition (MOEA/D), NSGA-III, and the constrained two-archive evolutionary algorithm (CTAEA).

In the benchmark-function experiments, the population size was set to 100 for the two-objective problems and 150 for the three-objective problems. All problems were run for a maximum of 250 generations. Each algorithm was independently run 30 times for each benchmark function, with a different random seed used in every run.

Algorithm performance was evaluated using hypervolume (HV), inverted generational distance (IGD), generational distance (GD), a distribution-dispersion metric defined in this study, and runtime. Higher HV values indicated broader coverage of the objective space, whereas lower IGD and GD values indicated smaller distances between the obtained solution set and the reference Pareto front.

Let the nondominated solution set obtained in a single run be

(1) $$F={\left\{f_{i}\right\}}_{i=1}^{N}$$

where $f_{i}$ denotes the objective vector of solution i, and N denotes the number of nondominated solutions. The centroid of the solution set was defined as

(2) $$\overset{‾}{f}=\frac{1}{N}\sum_{i=1}^{N}f_{i}$$

The Euclidean distance from solution i to the centroid was calculated as

(3) $$d_{i}={∥f_{i}-\overset{‾}{f}∥}_{2}$$

The distribution-dispersion metric was defined as the ratio of the standard deviation to the mean of these distances:

(4) $$\Delta_{p}=\frac{SD\left(d_{1},d_{2},\ldots ,d_{N}\right)}{Mean\left(d_{1},d_{2},\ldots ,d_{N}\right)}$$

A lower $\Delta_{p}$ value indicated less variation in the distances from the individual solutions to the centroid and, consequently, a more uniform distribution of the solution set.

For the application-specific multi-endpoint optimization task involving candidate compounds, 2P-HAMOEA was compared with the same five comparator algorithms. All algorithms were run in the same software and hardware environment according to a prespecified random-seed scheme. The comparator algorithms were implemented using the pymoo 0.6.1.6 framework with default or literature-recommended operator configurations.

The population size was set to 200 for all algorithms, and the main evolutionary stage was run for 300 generations. For 2P-HAMOEA, this stage was followed by 50 generations of local refinement, with the proportion of elite solutions set to 0.4. Random sampling, Tent chaotic mapping, and Lévy flight accounted for 30%, 40%, and 30% of the initial population, respectively.

Each algorithm was independently run 30 times, using a different random seed in each run. IGD, GD, HV, the number of nondominated solutions, and runtime were summarized as the mean ± standard deviation. For each algorithm, IGD and GD were calculated using a combined nondominated front constructed from the results of all other algorithms, excluding the algorithm under evaluation, as the reference front.

Overall differences among the six algorithms in IGD, GD, and HV were assessed using the Friedman test. When the overall difference was statistically significant, Wilcoxon signed-rank tests were performed to compare 2P-HAMOEA separately with each comparator algorithm. The number of nondominated solutions and runtime were compared descriptively only.

#### 4.4.4. Ablation Study

To assess the contributions of the individual components of 2P-HAMOEA under the current experimental settings, four ablation variants were constructed using the full algorithm (FULL) as the reference:

NO_MSI: multi-source initialization was removed and replaced with a single initialization method;

NO_CSO: the SOS-derived cooperative-search operators were removed while the NSGA-III reference-direction component was retained;

NO_LOCAL: the second-phase local-refinement procedure was removed;

FIXED_LOCAL: local refinement was retained, but the selection probabilities of DE, PSO, and SA were fixed.

Apart from the component removed or replaced in each variant, the objective functions, fitness evaluation, constraint handling, search-operator settings, and sources of randomness were kept unchanged. The population size was set to 200, the global-search stage was run for a maximum of 400 generations, and the local-refinement stage was run for a maximum of 60 generations. FULL and each ablation variant were independently run 30 times.

The variants were evaluated using IGD, GD, HV, spacing (SP), and runtime. Lower IGD, GD, and SP values and a higher HV indicated better performance according to the corresponding metric. Differences between FULL and each ablation variant in IGD, HV, and SP were evaluated using Wilcoxon signed-rank tests. GD and runtime were compared descriptively, without between-group statistical testing.

Because the ablation study and the algorithm comparison for the application-specific task used different numbers of evolutionary generations and different local-refinement settings, the absolute metric values from the two experiments were interpreted only within their respective experimental settings and were not compared directly across experiments.

### 4.5. Analysis of Relationships Among Endpoints and Evaluation of Feasible Pareto Solutions

#### 4.5.1. Analysis of Relationships Among Endpoints

Relationships between p*IC*_50_ and the five ADMET-related endpoints were first examined in the training set. Point-biserial correlation coefficients were used to quantify the linear associations between the continuous endpoint p*IC*_50_ and the binary endpoints Caco-2, CYP3A4, hERG, HOB, and MN. Associations among the five binary endpoints were assessed using Cramér’s V. Point-biserial correlation coefficients range from −1 to 1, with larger absolute values indicating stronger linear associations between a continuous and a binary variable. Cramér’s V ranges from 0 to 1, with higher values indicating stronger associations between categorical variables.

The 25th and 75th percentiles of p*IC*_50_ in the training set were used to define three bioactivity levels: low (p*IC*_50_ ≤ 5.77), intermediate (5.77 < p*IC*_50_ < 7.83), and high (p*IC*_50_ ≥ 7.83). These cutoffs were then applied to all 1875 retained samples. For visualization, p*IC*_50_ and the five ADMET-related endpoints were scaled using min–max normalization, and a parallel-coordinates plot was used to examine their joint distribution across the three bioactivity levels.

Mann–Whitney U tests were subsequently performed using all retained samples to compare the distributions of p*IC*_50_ between samples labeled 0 and 1 for each ADMET-related endpoint. The correlation analyses, between-group comparisons, and visualizations were descriptive and did not support causal inference.

#### 4.5.2. Comprehensive Evaluation of Feasible Pareto Solutions

All feasible Pareto solutions remaining after constraint handling, nondominance filtering, and diversity filtering were included in the comprehensive evaluation. The six endpoints were first transformed according to the optimization directions defined in Section 4.4.2 so that larger values consistently represented more desirable target states. Each endpoint was then scaled to the range of 0–1 using min–max normalization, thereby aligning both directionality and measurement scale.

Objective weights for the six endpoints were calculated independently using the Method Based on the Removal Effects of Criteria (MEREC) and the entropy weight method (EWM). MEREC assessed the contribution of each endpoint by measuring the change in the overall evaluation after that endpoint was removed. EWM assigned weights according to the dispersion and information content of each endpoint across the feasible Pareto solutions.

The two sets of weights were combined using an adaptive reliability-based fusion (ARF) strategy. For endpoint j, let $w_{j}^{M}$ and $w_{j}^{E}$ denote the weights obtained using MEREC and EWM, respectively, and let $r_{M}$ and $r_{E}$ denote the reliabilities of the two weighting methods. The fusion coefficient was calculated as

(5) $$\alpha =\frac{r_{M}}{r_{M}+r_{E}}$$

and the fused weight for endpoint j was calculated as

(6) $$w_{j}=\alpha w_{j}^{M}+\left(1-\alpha \right)w_{j}^{E}$$

The reliability of each weighting method was derived from the information entropy of its weight distribution. The coefficient α therefore represented the relative contribution of the MEREC weights to the fused weights. The resulting fused weights were used in the subsequent multi-criteria decision-making analysis.

Using the fused endpoint weights, the feasible Pareto solutions were evaluated using the Technique for Order Preference by Similarity to Ideal Solution (TOPSIS), the VIKOR compromise-ranking method, and the Weighted Aggregated Sum Product Assessment (WASPAS). TOPSIS ranked solutions according to their relative distances from the positive and negative ideal solutions. VIKOR balanced overall group utility against maximum individual regret, while WASPAS combined weighted-sum and weighted-product evaluations.

After the score directions from the three methods had been aligned and the scores standardized, they were integrated using a reliability-based weighted fusion (RWF) strategy. For feasible Pareto solution i, let $S_{i}^{T}$, $S_{i}^{V}$, and $S_{i}^{W}$ denote the standardized TOPSIS, VIKOR, and WASPAS scores, respectively. Let $\beta_{1}$, $\beta_{2}$, and $\beta_{3}$ denote the corresponding reliability weights, subject to

(7) $$\beta_{k}\geq 0,\;\sum_{k=1}^{3}\beta_{k}=1$$

The RWF composite score for solution i was calculated as

(8) $$RWF_{i}=\beta_{1}S_{i}^{T}+\beta_{2}S_{i}^{V}+\beta_{3}S_{i}^{W}$$

To characterize the extent to which the performance of an individual solution varied across endpoints, a performance uncertainty index (PUI) was calculated from the six directionally aligned and standardized endpoint values:

(9) $$PUI_{i}=SD\left(z_{i1},z_{i2},\ldots ,z_{i6}\right)$$

where $z_{ij}$ denotes the standardized value of endpoint j for solution i. A higher PUI indicated greater cross-endpoint dispersion within a solution, whereas a lower PUI indicated a more balanced endpoint profile. The PUI quantified dispersion across endpoints within an individual solution; it did not represent prediction error, a statistical confidence interval, or variability across repeated experiments.

The PUI values were normalized using min–max scaling and then reverse-transformed to obtain a stability weight for each solution:

(10) $$w_{i}^{PUI}=1-\frac{PUI_{i}-min\left(PUI\right)}{max\left(PUI\right)-min\left(PUI\right)}$$

The stability weight was applied as a multiplicative adjustment to the RWF composite score, yielding the robust weighted score (RWS):

(11) $$RWS_{i}=RWF_{i}\times w_{i}^{PUI}$$

All feasible Pareto solutions were ranked in descending order of RWS. The RWS incorporated a cross-endpoint dispersion adjustment into the multi-criteria decision-fusion score and was used only for relative comparisons within the current feasible solution set.

#### 4.5.3. Extraction of Value Intervals for Key Molecular Descriptors

After all feasible Pareto solutions had been ranked in descending order of RWS, the top 20% were selected as the high-scoring subset. In the present analysis, 155 feasible Pareto solutions were obtained; therefore, the high-scoring subset comprised the 31 highest-ranked solutions.

For each key molecular descriptor, the mean, standard deviation, and the 10th, 25th, 75th, and 90th percentiles were calculated within the high-scoring subset. Three statistical intervals were then defined:

Core interval: the 25th–75th percentile range (P25–P75), representing the middle 50% of the high-scoring subset;

Robust interval: the 10th–90th percentile range (P10–P90), covering approximately 80% of the high-scoring subset;

Extended interval: the mean ± standard deviation, reflecting the central tendency and dispersion of each molecular descriptor within the high-scoring subset.

All molecular descriptors were returned to their original scales through inverse transformation. The core, robust, and extended intervals were compared within each descriptor to characterize its distribution and degree of concentration among the 31 highest-ranked feasible Pareto solutions. Because the descriptors differed in their units and numerical scales, their absolute interval widths were not compared directly across descriptors.

Because the three interval types were based on different statistical measures, the extended interval was not expected to lie entirely within either percentile-based interval. All intervals describe the high-scoring subset obtained under the current data, prediction models, optimization constraints, weighting methods, and ranking rules. They summarize only the numerical characteristics of the key molecular descriptors within this subset and should not be interpreted as definitive structural thresholds validated through molecular structure reconstruction, chemical synthesis, biological experiments, or external data. Because these are marginal, descriptor-wise intervals, they do not preserve the joint dependency structure among descriptors and do not guarantee that an arbitrary combination is chemically realizable.

To test whether the descriptor intervals depended strongly on the predefined high-scoring-subset threshold, a targeted sensitivity analysis repeated the interval extraction using the top 10%, top 20%, and top 30% of the same 155 feasible Pareto solutions ranked in descending order of RWS. With upward rounding for non-integer subset sizes, these thresholds corresponded to 16, 31, and 47 solutions, respectively. The core, robust, and extended intervals were recalculated using the same definitions. Similarity between two intervals was summarized using an interval-overlap index, defined as the length of their intersection divided by the length of their union; values closer to 1 indicated greater agreement. This analysis evaluated the final subset-size threshold only and did not constitute a full-pipeline sensitivity analysis of the upstream preprocessing and descriptor-selection thresholds.

## 5. Conclusions

This study developed an integrated analytical workflow for candidate ERα antagonists that combined key molecular descriptor selection, multi-endpoint property prediction, multi-objective optimization in molecular descriptor space, and comprehensive evaluation of feasible Pareto solutions. The p*IC*_50_ outcome and each ADMET-related endpoint were modeled independently rather than as a combined response. Two-stage cross-method and cross-endpoint screening identified 24 key molecular descriptors that were jointly used to model p*IC*_50_ and the five ADMET-related endpoints. Different final models were selected across endpoints. The three-model stacking ensemble for p*IC*_50_ achieved an internal-holdout *R*^2^ of 0.650 (95% bootstrap CI, 0.549–0.728). A separate matched sensitivity comparison yielded very similar *R*^2^ values for the current 24 descriptors and a published alternative 20-descriptor set. The AUC values of the five selected ADMET classification models ranged from 0.878 to 0.979. These are internal estimates and do not establish performance on externally sourced or structurally novel chemotypes.

ERα inhibitory activity and the five ADMET-related properties did not exhibit uniform statistical relationships, supporting the use of multi-objective optimization to retain nondominated solutions with different endpoint profiles. Under the prespecified bioactivity and ADMET criteria, 2P-HAMOEA generated 155 feasible Pareto solutions. The algorithm showed favorable performance for selected solution-quality metrics, including GD, and the ablation study indicated that the SOS-derived cooperative-search operators were associated with improvements in selected metrics under the current experimental settings. However, the overall findings did not demonstrate that 2P-HAMOEA was comprehensively superior to conventional multi-objective optimization algorithms.

All 155 feasible Pareto solutions were evaluated and ranked using objective weighting, multi-criteria decision-making, reliability-based fusion, and a PUI-based cross-endpoint dispersion adjustment. The fused endpoint weights assigned the highest relative weight to p*IC*_50_, at 0.328, while the rankings generated by TOPSIS, VIKOR, and WASPAS showed high overall agreement. The top 20% of the feasible Pareto solutions according to RWS, comprising the 31 highest-ranked solutions, were subsequently used to derive core, robust, and extended intervals for the 24 key molecular descriptors. In practice, the workflow is designed to support early-stage comparison of explicitly defined candidate structures by first reviewing their six endpoint-specific predictions separately and then using Pareto and ranking results to examine trade-offs among those profiles. It does not generate molecular structures or establish substituent-level structure–activity relationships. The feasible Pareto solutions and descriptor intervals remain within-study computational references and require independent data, molecular structure generation, receptor-based assessment, evaluation of synthetic feasibility, and experimental validation.

## Figures and Tables

**Table 1:** Twenty-four key molecular descriptors identified through two-stage cross-method and cross-endpoint screening.

Molecular Descriptor	Structural or Physicochemical Information Encoded
ATSc1	Charge-weighted ATS autocorrelation
sumI	Sum of intrinsic-state values
BCUTc-1L	Lowest partial-charge-weighted BCUT value
SCH-7	Simple-chain index, order 7
WTPT-3	Heteroatom-originating weighted path lengths
VCH-6	Valence-chain index, order 6
VC-3	Valence-cluster index, order 3
TopoPSA	Topological polar surface area
nsssCH	Count of >CH-atom-type E-states
FMF	Molecular framework complexity
maxHBd	Maximum E-state of strong H-bond donors
minHBd	Minimum E-state of strong H-bond donors
minHBa	Minimum E-state of strong H-bond acceptors
SHBd	Sum of E-states of strong H-bond donors
ETA_BetaP_ns_d	Lone-electron resonance relative to molecular size
ETA_EtaP	Composite ETA index relative to molecular size
SsOH	Sum of -OH atom-type E-states
maxsOH	Maximum -OH atom-type E-state
minsOH	Minimum -OH atom-type E-state
nBondsD	Number of double bonds
maxHCsats	Maximum E-state for H attached to B/Si/P/Ge/As/Se/Sn/Pb
C2SP2	Doubly bonded carbon attached to two carbons
MLogP	Mannhold logP
LipoaffinityIndex	Lipoaffinity index

Note: Definitions follow the descriptor dictionary used with the study dataset. These descriptors encode whole-molecule or local atomic attributes and generally do not map one-to-one to a particular substituent. Their repeated selection indicates statistical consistency under the current data and screening procedure, rather than a causal structure–activity relationship.

**Table 2:** Final selected endpoint-specific prediction models and their performance in the internal holdout sets. (**A**) p*IC*_50_ regression model; (**B**) ADMET classification models.

(**A**)							
**Endpoint**		**Final Selected Model**		**MAE**	**MedAE**	**MSE**	***R* ^2^**
p*IC*_50_		Three-model stacking ensemble		0.573	0.421	0.627	0.650
(**B**)							
**Endpoint**	**Final Selected Model**		**Accuracy**	**Precision**	**Recall**	**AUC**	**F_1_**
Caco-2	LightGBM + TPE		0.867	0.851	0.805	0.933	0.828
CYP3A4	MLP + TPE + CS		0.904	0.921	0.959	0.936	0.939
hERG	Five-model simple averaging ensemble		0.829	0.805	0.936	0.878	0.866
HOB	GB + TPE		0.797	0.786	0.682	0.880	0.730
MN	LightGBM + CS		0.933	0.938	0.980	0.979	0.958

Note: TPE, tree-structured Parzen estimator; CS, cost-sensitive learning; MAE, mean absolute error; MedAE, median absolute error; MSE, mean squared error; *R*^2^, coefficient of determination. The first-layer base learners in the three-model stacking ensemble for p*IC*_50_ were Oblivious Tree + TPE, SVR + TPE, and XGBoost + TPE, with linear regression used as the second-layer meta-learner. The five-model simple averaging ensemble for hERG comprised GB + TPE, XGBoost + TPE, LightGBM + TPE, BPNN + TPE, and SVM. For hERG classification evaluation, binary predictions were averaged and thresholded at 0.5; the same learners’ class-1 probabilities were averaged for the continuous optimization objective. Final model selection followed the original multi-metric radar-area comparison reproduced in Supplementary Tables S5 and S6. Bootstrap 95% confidence intervals are provided in Supplementary Table S8.

**Table 3:** Core performance metrics of the six algorithms in the application-specific multi-endpoint optimization task.

Algorithm	IGD	GD	HV	Number of Nondominated Solutions	Runtime (s)
NSGA-II	0.540 ± 0.031	0.372 ± 0.029	166.999 ± 7.890	200.000 ± 0.000	71.813 ± 5.780
SPEA2	0.575 ± 0.058	0.339 ± 0.024	161.662 ± 4.801	200.000 ± 0.000	99.940 ± 0.597
MOEA/D	1.144 ± 0.133	0.087 ± 0.038	158.404 ± 11.950	49.867 ± 7.300	2595.651 ± 209.059
NSGA-III	0.479 ± 0.061	0.156 ± 0.009	183.599 ± 3.403	191.100 ± 13.586	81.549 ± 2.870
CTAEA	0.346 ± 0.027	0.319 ± 0.015	194.872 ± 2.136	462.000 ± 0.000	247.334 ± 20.467
2P-HAMOEA	0.753 ± 0.039	0.092 ± 0.012	163.821 ± 2.507	200.100 ± 0.403	255.573 ± 30.081

Note: Results are presented as the mean ± standard deviation from 30 independent runs. Lower IGD and GD values and a higher HV indicate better performance according to the corresponding metric. For each algorithm, IGD and GD were calculated using a combined nondominated front constructed from the results of the other algorithms as the reference front. Overall differences among the six algorithms in IGD, GD, and HV were first assessed using the Friedman test. When an overall difference was statistically significant, Wilcoxon signed-rank tests were performed to compare 2P-HAMOEA separately with each comparator algorithm. The number of nondominated solutions and runtime were compared descriptively only.

**Table 4:** Results of the 2P-HAMOEA ablation study.

Algorithm	IGD	GD	HV	SP	Runtime (s)
FULL	0.13 ± 0.01	0.05 ± 0.01	36.75 ± 9.00	0.09 ± 0.01	193.18 ± 11.53
NO_MSI	0.14 ± 0.02	0.05 ± 0.02	36.37 ± 7.62	0.09 ± 0.01	205.19 ± 3.21
NO_CSO	0.15 ± 0.01	0.06 ± 0.01	35.40 ± 4.71	0.09 ± 0.01	117.03 ± 2.88
NO_LOCAL	0.14 ± 0.01	0.05 ± 0.01	36.43 ± 9.02	0.09 ± 0.01	79.34 ± 3.73
FIXED_LOCAL	0.14 ± 0.01	0.05 ± 0.01	36.44 ± 9.02	0.09 ± 0.01	119.12 ± 2.57

Note: FULL, full 2P-HAMOEA; NO_MSI, 2P-HAMOEA without multi-source initialization; NO_CSO, 2P-HAMOEA without the SOS-derived cooperative-search operators; NO_LOCAL, 2P-HAMOEA without local refinement; FIXED_LOCAL, 2P-HAMOEA with fixed local-operator selection probabilities. Lower IGD, GD, and SP values and a higher HV indicate better performance according to the respective metric. Differences between FULL and each ablation variant in IGD, HV, and SP were evaluated using Wilcoxon signed-rank tests. GD and runtime were compared descriptively only. Because the ablation study and the algorithm comparison reported in Table 3 used different numbers of evolutionary generations and different local-refinement settings, their absolute metric values should not be compared directly. For consistency of presentation, all means and standard deviations in this table are rounded to two decimal places; statistical comparisons were performed using the unrounded values. The identical displayed GD values for FULL, NO_LOCAL, and FIXED_LOCAL reflect rounding rather than identical underlying estimates.

**Table 5:** MEREC, EWM, and fused weights of the six endpoints.

Endpoint	MEREC Weight	EWM Weight	Fused Weight
p*IC*_50_	0.265	0.419	0.328
Caco-2	0.155	0.161	0.158
CYP3A4	0.193	0.012	0.120
hERG	0.095	0.054	0.079
HOB	0.133	0.153	0.141
MN	0.157	0.201	0.175

Note: MEREC, Method Based on the Removal Effects of Criteria; EWM, entropy weight method. The fused weights were calculated using the adaptive reliability-based fusion strategy from the current set of 155 feasible Pareto solutions. The sums of the displayed three-decimal weights are 0.998, 1.000, and 1.001 for the MEREC, EWM, and fused-weight columns, respectively; each unrounded weight vector was normalized to sum to 1.000. These weights reflect relative contributions within the current comprehensive evaluation and should not be interpreted as measures of absolute pharmacological importance.

**Table 6:** Label definitions for the five ADMET-related endpoints.

Endpoint	Property Evaluated	Label	Definition
Caco-2	Cell permeability	0	Relatively poor cell permeability
		1	Relatively favorable cell permeability
CYP3A4	Whether the compound can be metabolized by CYP3A4	0	Cannot be metabolized by CYP3A4
		1	Can be metabolized by CYP3A4
hERG	Cardiotoxicity	0	Cardiotoxicity absent
		1	Cardiotoxicity present
HOB	Human oral bioavailability	0	Relatively poor oral bioavailability
		1	Relatively favorable oral bioavailability
MN	Genotoxicity	0	Genotoxicity absent
		1	Genotoxicity present

Note: The labels shown are the original class codes provided in the dataset. For Caco-2 and HOB, a label of 1 denotes a relatively favorable property, whereas for hERG and MN, a label of 1 indicates the presence of the corresponding toxicity risk. The CYP3A4 label indicates only whether a compound can be metabolized by this enzyme and should not be interpreted as direct evidence of superior safety or developability. The optimization directions and feasibility criteria for these endpoints are described in Section 4.4.2.

## Data Availability

The data analyzed in this study were obtained from Problem D, “Optimization Modeling of Candidate Anti-Breast-Cancer Drugs,” of the 18th China Postgraduate Mathematical Contest in Modeling. The complete original compound-level dataset (1974 compounds, SMILES strings, endpoint labels, and 729 precomputed descriptors), the processed 1875-compound analytical dataset with the retained 24 descriptors, the realized fixed training/internal-holdout assignments and associated model-ready descriptor matrix, and the analytical results supporting the findings of this study are available from the corresponding author upon request. The authors are willing to provide these materials for legitimate research and verification purposes. The original descriptor-generation software and version were not documented in the source data, as stated in the Limitations.

## Supplementary Materials

The following supporting information can be downloaded at https://www.mdpi.com/article/10.3390/ph19091453/s1: Figure S1: Precision-recall curves of the final selected models for five ADMET-related endpoints in the internal holdout sets; Figure S2: Confusion matrices of the final selected models for five ADMET-related endpoints in the internal holdout sets; Figure S3: Joint t-SNE visualization of the fixed training and internal-holdout assignment in the shared 24-descriptor space; Table S1: Feasible Pareto-optimal solutions: molecular descriptor values and predicted endpoint outputs; Table S2: Distributional intervals of 24 key molecular descriptors among high-scoring Pareto-optimal solutions; Table S3: Sensitivity of descriptor intervals to the high-scoring-subset threshold; Table S4: Matched comparison of p*IC*_50_ stacking performance using the current 24-descriptor set and the Dong et al. 20-descriptor set; Table S5: Original training-set and internal-holdout results for candidate p*IC*_50_ regression models and ensemble alternatives: (**A**) training-set tenfold cross-validation and radar-area ranking; (**B**) internal-holdout comparison of shortlisted models and ensemble configurations; Table S6: Internal-holdout performance and radar-area summaries of shortlisted ADMET candidate models; Table S7: Tenfold cross-validation performance of selected ADMET individual models and hERG ensemble constituents; Table S8: Bootstrap 95% confidence intervals for final internal-holdout metrics; Table S9: Leverage-based descriptor-space applicability-domain summary.
