# Supplementary material for: Multi-Endpoint Prediction of Bioactivity and ADMET Properties Coupled with Multi-Objective Optimization in Molecular Descriptor Space for Candidate ERα Antagonists
Source: Pharmaceuticals (Basel). 2026 Sep 14;19(9):1453. doi: 10.3390/ph19091453 (PMC13610615; doi:10.3390/ph19091453)
Supplement: Supplementary file 1 [file pharmaceuticals-19-01453-s001.zip › pharmaceuticals-4487595-supplementary.pdf]

Supplementary Materials

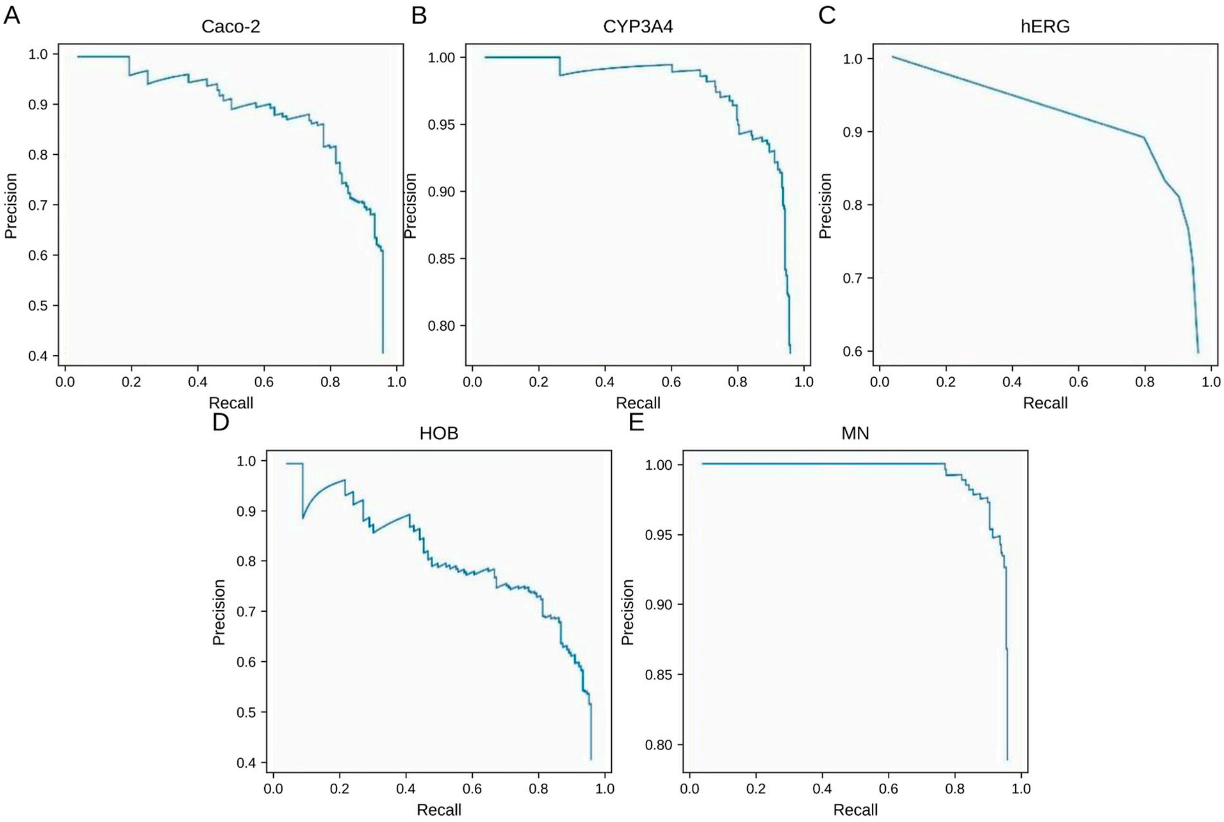

Figure S1. Precision-recall curves of the final selected models for five ADMET-related endpoints in the internal holdout sets. Panels A–E show the precision-recall curves of the final selected models for Caco-2, CYP3A4, hERG, HOB, and MN, respectively. The corresponding models were LightGBM + TPE, MLP + TPE + CS, a five-model simple-averaging ensemble, GB + TPE, and LightGBM + CS. For hERG classification evaluation, the five thresholded constituent-model predictions were averaged, producing a vote fraction that was thresholded at 0.5 for the final class; the same five class-1 probabilities were averaged separately to provide the continuous optimization objective. Class 1 was treated as the positive class for metric calculation. Its interpretation was Caco-2 = relatively favorable permeability, CYP3A4 = metabolized by CYP3A4, hERG = cardiotoxicity present, HOB = relatively favorable oral bioavailability, and MN = genotoxicity present. Thus, class 1 did not uniformly denote a favorable target state. Each endpoint-specific internal holdout set contained 375 samples. Abbreviations: PR, precision-recall; TPE, tree-structured Parzen estimator; CS, cost-sensitive learning; GB, gradient boosting; MLP, multilayer perceptron; hERG, human ether-a-go-go-related gene; HOB, human oral bioavailability; MN, micronucleus.

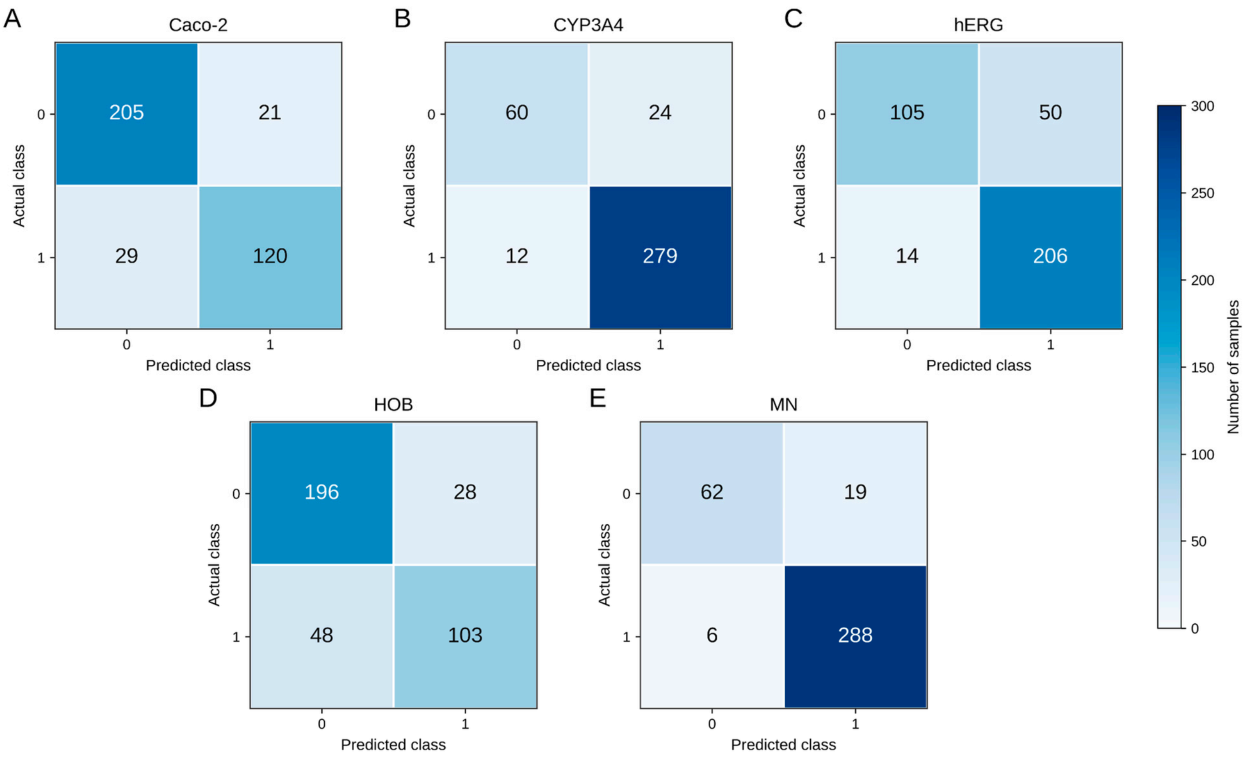

Figure S2. Confusion matrices of the final selected models for five ADMET-related endpoints in the internal holdout sets. Panels A–E show the confusion matrices of the final selected models for Caco-2, CYP3A4, hERG, HOB, and MN, respectively. The corresponding models were LightGBM + TPE, MLP + TPE + CS, a five-model simple-averaging ensemble, GB + TPE, and LightGBM + CS. For hERG classification evaluation, the five thresholded constituent-model predictions were averaged and the resulting vote fraction was thresholded at 0.5. Rows represent actual classes, columns represent predicted classes, and cell values indicate the corresponding numbers of samples. Class 1 was treated as the positive class for metric calculation. Its interpretation was Caco-2 = relatively favorable permeability, CYP3A4 = metabolized by CYP3A4, hERG = cardiotoxicity present, HOB = relatively favorable oral bioavailability, and MN = genotoxicity present. Thus, class 1 did not uniformly denote a favorable target state. A common count-based color scale was used across panels, and each endpoint-specific internal holdout set contained 375 samples.

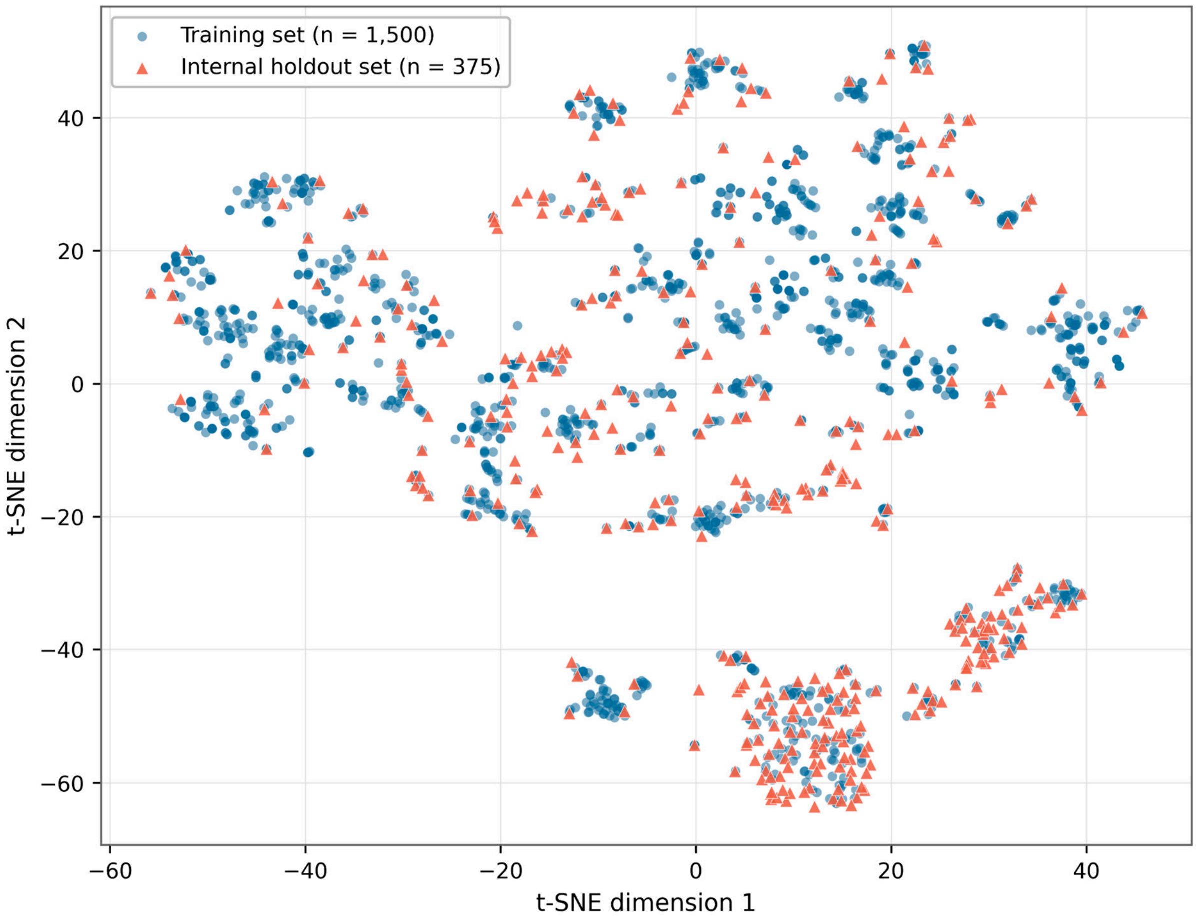

Figure S3. Joint t-SNE visualization of the fixed training and internal-holdout assignment in the shared 24-descriptor space. A single two-dimensional embedding was fitted jointly to all 1,875 standardized rows; the training/internal-holdout labels were applied only after embedding. Blue circles denote the training set (n = 1,500), and orange triangles denote the internal holdout (n = 375). Settings: n_components = 2, perplexity = 30, max_iter = 1,000, random_state = 42, PCA initialization, and automatic learning-rate selection. The same fixed compound assignment was used for pIC_{50} and all five ADMET endpoints. The plot is a descriptive visualization of the selected descriptor space and is not evidence of scaffold novelty, predictive performance, or external generalizability.

Table S1. Feasible Pareto-optimal solutions: molecular descriptor values and predicted endpoint outputs. Because the complete table contains 31 columns, the descriptor set is displayed in two horizontal blocks; rows correspond across blocks by Solution ID.

Solution ID	Molecular descriptors 1–12												Predicted endpoint outputs (6)					
	ATSc1	Lipoaffinity Index	sumI	BCUTc-1L	SCH-7	WTPT-3	SHBd	VCH-6	ETA_BetaP_ns_d	maxHBd	SsOH	TopoPSA	pIC_{50}	Caco-2	CYP3A4	hERG	HOB	MN
1	0.265	2.594	86.633	-0.386	0.754	9.307	1.027	0.096	0.104	0.631	26.792	42.077	8.050	0	1	0	1	0
2	0.451	10.863	62.128	-0.374	0.896	6.656	1.030	0.042	0.117	0.426	25.583	41.770	8.120	1	1	0	1	0
3	0.426	9.281	91.773	-0.367	0.991	11.389	1.034	0.046	0.103	0.406	24.026	42.369	8.421	1	1	0	1	1
4	0.271	1.773	80.849	-0.363	0.982	5.866	1.186	0.067	0.102	0.508	28.223	42.107	7.890	0	1	0	1	0
5	0.313	9.297	59.850	-0.363	0.989	9.310	1.032	0.027	0.104	0.631	27.553	33.174	8.236	1	1	0	0	0
6	0.311	8.714	79.467	-0.348	0.969	9.111	0.978	0.044	0.103	0.388	28.028	44.725	7.981	1	1	0	1	0
7	0.287	9.272	87.424	-0.388	0.897	5.716	1.059	0.043	0.111	0.417	29.480	105.322	8.024	1	1	0	1	0
8	0.305	9.149	86.231	-0.378	0.939	10.637	0.565	0.045	0.103	0.406	24.131	80.069	8.091	1	1	0	0	0
9	0.430	9.274	81.006	-0.363	0.988	9.321	1.029	0.045	0.109	0.336	26.345	40.128	8.480	1	1	0	0	0
10	0.313	9.185	82.143	-0.374	0.936	6.524	0.917	0.019	0.104	0.358	24.329	40.568	8.115	0	1	0	1	0
11	0.347	10.354	85.580	-0.373	0.982	12.011	0.773	0.045	0.104	0.385	28.059	43.380	7.877	0	1	0	1	0
12	0.440	9.154	88.212	-0.374	0.978	4.024	1.061	0.043	0.109	0.407	27.925	41.018	8.256	1	1	0	1	0
13	0.313	9.304	59.532	-0.374	0.937	6.508	1.061	0.113	0.103	0.359	24.380	33.235	8.240	0	1	0	0	0
14	0.270	1.976	59.010	-0.374	0.999	6.621	1.030	0.043	0.104	0.417	24.636	41.019	7.965	1	1	0	1	0
15	0.319	9.198	88.329	-0.364	0.972	9.299	0.988	0.026	0.103	0.354	27.795	33.148	8.288	0	1	0	1	0
16	0.303	9.156	85.946	-0.377	1.076	10.637	0.973	0.045	0.103	0.627	27.967	80.460	8.016	1	1	0	0	1
17	0.426	9.266	80.849	-0.375	0.953	5.804	1.071	0.067	0.102	0.405	27.988	42.107	8.162	0	1	0	1	0
18	0.430	9.076	85.859	-0.369	1.010	10.844	1.051	0.043	0.104	0.406	27.979	35.900	8.382	0	1	0	1	0
19	0.265	9.355	83.612	-0.387	0.984	10.162	1.059	0.043	0.103	0.354	24.492	33.152	7.896	1	1	0	1	0
20	0.430	9.947	84.344	-0.380	0.987	9.591	0.934	0.016	0.109	0.405	27.116	37.348	8.247	1	1	0	1	0
21	0.241	6.551	83.964	-0.362	0.941	7.745	1.061	0.068	0.103	0.641	27.505	41.198	7.904	1	1	0	0	0
22	0.233	9.421	66.862	-0.398	1.005	11.675	1.054	0.043	0.103	0.545	23.804	39.107	8.249	1	1	0	0	0
23	0.427	9.439	59.851	-0.367	1.002	9.313	1.030	0.015	0.109	0.334	24.481	40.989	8.376	1	1	0	1	0
24	0.424	10.352	81.282	-0.350	0.976	5.929	1.140	0.045	0.103	0.408	24.356	40.630	8.016	1	1	0	1	0
25	0.308	8.708	85.931	-0.386	0.979	10.632	1.158	0.045	0.103	0.627	27.924	40.649	8.275	1	1	0	1	0
26	0.273	9.654	58.314	-0.374	0.937	9.283	1.141	0.046	0.107	0.420	28.566	41.592	8.267	1	1	0	1	0
27	0.265	2.594	86.633	-0.386	0.952	9.307	1.027	0.096	0.104	0.631	24.021	42.077	8.071	1	1	0	0	0
28	0.269	1.737	80.567	-0.380	0.980	9.185	0.970	0.045	0.104	0.630	24.830	41.366	7.961	1	1	0	1	0
29	0.429	9.796	85.857	-0.363	0.972	9.201	0.978	0.049	0.103	0.359	28.028	41.446	8.428	1	1	0	1	0

Solution ID	Molecular descriptors 1–12												Predicted endpoint outputs (6)					
	ATSc1	Lipoaffinity Index	sumI	BCUTc-1L	SCH-7	WTPT-3	SHBd	VCH-6	ETA_BetaP_ns_d	maxHBd	SsOH	TopoPSA	pIC_{50}	Caco-2	CYP3A4	hERG	HOB	MN
30	0.441	8.735	80.100	-0.363	1.110	9.351	1.139	0.044	0.102	0.408	30.805	43.616	8.012	1	1	0	1	1
31	0.317	9.654	58.314	-0.374	0.937	9.283	0.939	0.019	0.107	0.420	28.566	41.592	8.214	1	1	0	1	0
32	0.311	8.714	85.857	-0.386	0.971	9.132	0.978	0.049	0.103	0.357	28.028	41.127	8.145	1	1	0	1	0
33	0.444	9.077	59.732	-0.367	1.043	6.693	0.970	0.039	0.109	0.485	29.527	42.277	8.318	1	1	0	1	1
34	0.319	9.200	103.322	-0.363	0.971	9.299	0.988	0.046	0.103	0.354	27.864	33.148	8.233	0	1	0	1	0
35	0.439	9.775	80.100	-0.348	1.044	8.246	1.138	0.044	0.107	0.408	26.811	43.721	7.976	1	1	0	1	1
36	0.426	9.838	88.212	-0.368	0.980	4.024	1.059	0.043	0.092	0.400	27.925	41.374	8.135	1	1	0	1	0
37	0.368	9.296	104.193	-0.369	0.986	15.254	1.182	0.026	0.105	0.414	30.521	38.997	7.919	1	1	0	1	0
38	0.447	9.002	54.280	-0.387	0.985	12.716	1.073	0.043	0.109	0.410	27.899	34.974	7.997	1	1	0	1	0
39	0.439	9.077	83.471	-0.379	1.044	6.723	1.028	0.027	0.104	0.485	29.527	42.277	8.367	1	1	0	1	1
40	0.269	1.737	80.567	-0.372	0.980	9.234	0.969	0.045	0.113	0.630	24.820	41.366	7.967	1	1	0	1	0
41	0.444	8.735	80.100	-0.363	0.969	9.167	1.138	0.044	0.102	0.408	27.935	43.616	8.306	1	1	0	1	0
42	0.311	8.714	82.006	-0.386	0.969	9.111	0.978	0.044	0.102	0.357	28.028	41.030	8.120	1	1	0	1	0
43	0.265	6.519	86.838	-0.366	1.001	9.314	1.080	0.027	0.103	0.341	23.985	40.136	8.004	1	1	0	1	0
44	0.274	1.737	85.979	-0.380	0.979	9.190	1.059	0.045	0.104	0.630	24.843	41.366	8.000	1	1	0	1	0
45	0.297	9.235	79.564	-0.349	0.854	10.654	1.001	0.036	0.103	0.388	26.132	64.284	8.004	1	1	0	1	0
46	0.288	9.632	58.314	-0.374	0.979	11.553	1.098	0.046	0.103	0.319	27.923	41.592	8.340	1	1	0	1	0
47	0.265	8.891	90.868	-0.386	0.952	9.307	1.138	0.096	0.103	0.631	24.375	42.092	8.143	1	1	0	0	0
48	0.427	9.439	64.396	-0.388	0.952	5.906	1.190	0.015	0.104	0.404	24.611	42.094	8.061	1	1	0	1	0
49	0.430	9.295	91.549	-0.363	0.986	9.206	1.038	0.035	0.104	0.411	24.097	106.305	7.987	1	1	0	1	1
50	0.306	9.269	85.961	-0.379	0.932	5.937	1.143	0.046	0.103	0.594	27.933	46.164	8.430	1	1	0	0	0
51	0.379	9.227	85.417	-0.363	0.974	9.621	1.146	0.032	0.102	0.332	28.556	42.570	8.056	1	1	0	1	1
52	0.308	1.646	88.552	-0.379	0.896	5.627	1.166	0.044	0.109	0.410	29.510	28.581	7.944	1	1	0	1	0
53	0.364	9.203	88.212	-0.374	0.849	4.050	1.061	0.043	0.109	0.407	27.795	33.149	8.458	1	1	0	1	0
54	0.427	9.439	64.396	-0.387	1.002	11.674	1.059	0.015	0.104	0.307	24.611	40.996	8.102	1	1	0	1	1
55	0.310	9.003	88.212	-0.380	0.836	11.414	1.060	0.043	0.108	0.404	24.924	41.170	8.169	0	1	0	1	1
56	0.427	9.074	101.686	-0.378	1.003	3.802	1.061	0.044	0.110	0.409	28.282	36.480	8.018	1	1	0	1	0
57	0.449	9.238	84.053	-0.387	1.036	11.915	1.059	0.046	0.103	0.494	27.974	41.716	8.241	1	1	0	1	1
58	0.266	1.699	60.482	-0.363	0.974	6.745	1.056	0.043	0.103	0.615	28.536	44.894	8.088	1	1	0	1	1
59	0.439	10.158	80.100	-0.348	1.044	8.246	1.141	0.044	0.107	0.408	21.453	43.721	7.913	1	1	0	1	0
60	0.265	1.852	86.885	-0.379	0.749	9.314	0.983	0.095	0.103	0.341	26.784	40.136	8.083	0	1	0	1	0

Solution ID	Molecular descriptors 1–12												Predicted endpoint outputs (6)					
	ATSc1	Lipoaffinity Index	sumI	BCUTc-1L	SCH-7	WTPT-3	SHBd	VCH-6	ETA_BetaP_ns_d	maxHBd	SsOH	TopoPSA	pIC_{50}	Caco-2	CYP3A4	hERG	HOB	MN
61	0.314	9.637	57.962	-0.374	0.937	11.506	1.146	0.046	0.103	0.319	27.801	42.546	8.159	1	1	0	1	1
62	0.451	2.316	89.560	-0.369	0.978	6.582	1.147	0.045	0.104	0.414	29.117	41.833	7.855	1	1	0	1	0
63	0.463	9.080	81.283	-0.380	0.991	4.030	1.061	0.043	0.109	0.408	24.357	29.606	8.203	1	1	0	1	0
64	0.364	8.903	91.564	-0.397	0.849	4.050	1.061	0.043	0.109	0.407	27.795	33.149	8.197	1	1	0	1	0
65	0.266	1.671	61.499	-0.363	1.038	6.779	1.062	0.043	0.103	0.615	28.536	45.177	8.054	1	1	0	1	1
66	0.283	1.941	87.449	-0.398	0.988	10.058	1.057	0.043	0.109	0.417	29.903	39.227	7.960	1	1	0	1	0
67	0.454	9.544	61.841	-0.369	1.006	9.667	0.989	0.045	0.109	0.547	25.516	39.370	8.447	1	1	0	0	0
68	0.426	9.268	90.209	-0.378	0.982	6.649	1.059	0.020	0.103	0.408	27.988	26.758	8.159	1	1	0	1	0
69	0.425	9.118	83.473	-0.379	1.043	6.723	1.028	0.047	0.104	0.485	28.068	42.022	8.399	1	1	0	1	0
70	0.299	9.397	54.280	-0.378	0.975	5.580	1.074	0.046	0.103	0.408	27.897	41.565	8.174	1	1	0	1	0
71	0.287	9.272	88.552	-0.379	0.896	5.716	1.131	0.043	0.104	0.405	29.475	105.322	8.079	1	1	0	1	0
72	0.470	9.815	61.525	-0.376	1.036	4.930	1.146	0.017	0.103	0.335	27.360	52.917	7.848	1	1	0	1	0
73	0.296	9.227	85.551	-0.379	0.974	5.567	0.978	0.036	0.103	0.412	27.966	39.270	7.837	1	1	0	1	0
74	0.315	9.304	59.532	-0.374	0.932	8.972	1.062	0.019	0.103	0.360	24.511	33.235	8.135	1	1	0	1	0
75	0.276	10.569	75.392	-0.378	1.039	9.325	0.623	0.025	0.103	0.593	30.363	63.994	8.002	1	1	0	1	1
76	0.299	9.397	86.734	-0.378	0.986	5.580	1.069	0.046	0.103	0.408	27.903	41.565	8.107	1	1	0	1	0
77	0.267	9.149	86.231	-0.378	0.939	9.342	1.097	0.045	0.103	0.406	24.205	80.069	7.939	1	1	0	0	0
78	0.429	9.439	64.107	-0.386	0.973	9.246	0.979	0.015	0.104	0.307	28.167	40.996	7.836	1	1	0	1	0
79	0.437	9.173	91.824	-0.374	1.005	9.085	1.002	0.039	0.105	0.423	23.809	107.623	7.962	1	1	0	1	1
80	0.313	9.297	59.850	-0.363	1.047	6.814	1.032	0.027	0.104	0.631	27.553	33.174	8.144	1	1	0	0	0
81	0.434	6.707	86.832	-0.363	1.040	10.618	1.039	0.017	0.103	0.341	27.735	45.235	7.834	0	1	0	1	1
82	0.426	7.632	91.773	-0.367	0.989	9.284	1.036	0.051	0.093	0.406	24.026	40.916	8.053	1	1	0	1	0
83	0.364	9.591	91.564	-0.397	0.849	4.050	1.061	0.025	0.109	0.421	25.644	33.240	8.068	1	1	0	1	0
84	0.276	10.569	75.392	-0.388	1.037	9.323	0.623	0.025	0.103	0.593	30.363	63.791	7.884	1	1	0	1	0
85	0.362	9.132	88.552	-0.379	0.849	5.656	1.063	0.044	0.109	0.407	27.768	33.039	8.416	1	1	0	1	0
86	0.299	9.395	87.260	-0.368	1.002	5.580	1.070	0.015	0.103	0.409	27.903	41.565	8.201	1	1	0	1	0
87	0.468	9.747	57.705	-0.374	0.934	14.332	1.157	0.018	0.109	0.394	27.769	41.525	8.010	1	1	0	0	0
88	0.448	9.158	87.770	-0.363	0.985	9.742	1.071	0.044	0.104	0.356	24.689	39.158	7.995	1	1	0	1	0
89	0.435	2.601	86.531	-0.387	0.744	7.020	1.013	0.044	0.103	0.409	24.310	42.236	7.879	0	1	0	1	0
90	0.380	10.789	85.417	-0.374	0.974	13.811	1.148	0.032	0.102	0.332	28.556	42.559	8.149	1	1	0	1	0
91	0.310	9.004	88.206	-0.380	0.973	9.275	1.061	0.043	0.108	0.404	24.924	41.170	8.266	1	1	0	1	0

Solution ID	Molecular descriptors 1–12												Predicted endpoint outputs (6)					
	ATSc1	Lipoaffinity Index	sumI	BCUTc-1L	SCH-7	WTPT-3	SHBd	VCH-6	ETA_BetaP_ns_d	maxHBd	SsOH	TopoPSA	pIC_{50}	Caco-2	CYP3A4	hERG	HOB	MN
92	0.452	9.157	58.809	-0.387	1.036	12.486	1.059	0.046	0.103	0.493	27.974	41.079	7.851	1	1	0	1	1
93	0.311	9.431	103.082	-0.388	0.980	10.049	1.156	0.046	0.103	0.370	27.865	42.420	7.955	1	1	0	1	0
94	0.425	9.615	82.758	-0.374	1.043	6.723	1.029	0.047	0.104	0.485	28.075	41.601	8.364	1	1	0	0	0
95	0.311	9.072	85.887	-0.348	1.009	9.122	1.051	0.044	0.103	0.406	28.028	44.725	8.085	1	1	0	1	0
96	0.313	8.722	82.007	-0.368	0.974	14.264	0.963	0.043	0.103	0.357	28.042	105.148	8.065	0	1	0	1	1
97	0.439	9.077	83.471	-0.363	1.044	6.726	1.028	0.021	0.104	0.485	29.510	41.428	8.278	0	1	0	1	0
98	0.298	9.405	86.734	-0.378	0.986	5.549	1.069	0.046	0.103	0.408	27.903	41.565	8.167	1	1	0	0	0
99	0.318	9.280	81.399	-0.344	0.984	3.714	1.140	0.019	0.107	0.405	28.032	31.730	8.074	1	1	0	1	0
100	0.288	9.385	87.424	-0.388	0.949	5.716	1.061	0.015	0.111	0.405	30.582	105.322	7.888	1	1	0	1	0
101	0.427	9.028	98.066	-0.367	1.002	9.313	1.030	0.016	0.109	0.334	24.497	41.061	8.163	1	1	0	0	0
102	0.444	9.077	59.732	-0.367	1.043	9.310	0.970	0.039	0.109	0.373	29.527	42.277	8.199	1	1	0	1	0
103	0.274	9.622	59.081	-0.380	0.937	9.296	1.066	0.046	0.103	0.421	25.648	46.033	8.385	1	1	0	1	0
104	0.443	9.266	83.204	-0.379	1.042	6.726	1.029	0.027	0.104	0.486	29.475	41.401	8.160	1	1	0	1	0
105	0.439	9.238	83.825	-0.387	1.036	11.915	1.059	0.046	0.104	0.492	27.974	41.828	8.248	1	1	0	1	0
106	0.428	9.496	64.396	-0.363	1.003	11.674	1.059	0.015	0.103	0.312	24.562	40.692	8.346	1	1	0	1	1
107	0.272	9.473	58.580	-0.348	0.999	6.627	1.138	0.044	0.104	0.408	26.811	43.721	8.073	1	1	0	1	0
108	0.451	10.338	85.456	-0.348	1.046	8.209	1.130	0.045	0.103	0.385	28.059	43.797	7.845	0	1	0	1	0
109	0.269	1.737	80.565	-0.380	0.985	9.185	0.970	0.045	0.104	0.408	24.830	41.366	7.932	1	1	0	1	0
110	0.470	11.210	61.525	-0.376	1.036	4.930	1.146	0.017	0.103	0.335	28.026	52.917	7.965	1	1	0	1	0
111	0.427	9.796	86.146	-0.365	1.002	11.629	1.059	0.049	0.103	0.359	24.473	41.446	8.607	0	1	0	1	0
112	0.269	1.976	58.813	-0.363	0.999	6.482	1.038	0.043	0.104	0.417	24.636	41.060	8.005	1	1	0	1	0
113	0.449	10.974	62.242	-0.374	0.889	9.314	0.980	0.095	0.103	0.340	25.573	40.136	8.355	0	1	0	0	0
114	0.444	9.077	64.396	-0.367	1.043	6.693	0.970	0.039	0.104	0.306	29.552	40.887	8.353	1	1	0	1	0
115	0.436	9.278	59.541	-0.373	0.991	11.419	0.885	0.015	0.103	0.515	26.458	42.398	8.029	1	1	0	1	0
116	0.283	1.737	87.434	-0.408	0.988	13.572	1.057	0.043	0.109	0.417	24.888	41.380	7.836	1	1	0	1	1
117	0.427	9.441	59.326	-0.367	0.986	9.313	1.030	0.046	0.109	0.334	24.481	40.989	8.340	1	1	0	1	0
118	0.326	9.109	86.306	-0.365	0.987	10.672	1.029	0.046	0.105	0.627	24.464	33.468	8.090	1	1	0	0	0
119	0.287	9.272	88.552	-0.379	0.897	4.118	1.131	0.043	0.104	0.336	22.755	105.322	8.132	0	1	0	1	1
120	0.444	8.735	80.660	-0.373	0.969	9.138	1.138	0.044	0.102	0.353	27.935	43.616	8.180	1	1	0	1	1
121	0.584	8.544	86.431	-0.363	0.853	11.775	1.014	0.046	0.103	0.408	27.981	33.139	8.130	0	1	0	1	1
122	0.288	9.135	59.029	-0.379	0.979	11.553	1.097	0.046	0.103	0.319	27.916	42.013	8.405	1	1	0	1	0

Solution ID	Molecular descriptors 1–12												Predicted endpoint outputs (6)					
	ATSc1	Lipoaffinity Index	sumI	BCUTc-1L	SCH-7	WTPT-3	SHBd	VCH-6	ETA_BetaP_ns_d	maxHBd	SsOH	TopoPSA	pIC_{50}	Caco-2	CYP3A4	hERG	HOB	MN
123	0.430	8.717	79.439	-0.369	0.970	10.833	0.978	0.043	0.104	0.388	27.979	35.900	8.369	1	1	0	1	0
124	0.318	9.253	80.719	-0.344	0.987	5.960	0.928	0.019	0.107	0.405	27.912	31.730	7.985	1	1	0	1	0
125	0.424	9.281	85.603	-0.350	0.767	5.538	1.152	0.047	0.103	0.408	24.281	33.139	8.257	1	1	0	1	0
126	0.431	9.274	81.006	-0.363	0.988	9.321	1.029	0.045	0.104	0.336	26.345	40.128	8.027	1	1	0	1	0
127	0.286	8.875	82.448	-0.379	0.849	11.294	1.166	0.043	0.109	0.419	27.518	33.167	8.015	1	1	0	1	0
128	0.306	9.266	85.594	-0.379	0.932	5.937	1.143	0.076	0.103	0.594	27.933	42.482	8.424	1	1	0	0	0
129	0.292	9.164	88.212	-0.375	0.972	4.050	1.061	0.041	0.109	0.406	27.795	33.149	8.463	1	1	0	1	0
130	0.449	9.077	59.959	-0.374	0.933	6.693	0.966	0.044	0.109	0.395	24.509	33.105	8.132	1	1	0	1	0
131	0.266	1.676	61.499	-0.387	1.038	6.779	1.062	0.043	0.104	0.610	28.585	45.481	7.873	1	1	0	1	1
132	0.288	8.325	85.966	-0.365	1.093	5.696	0.983	0.043	0.103	0.341	23.926	39.271	7.958	0	1	0	1	0
133	0.274	1.737	86.255	-0.373	0.979	9.190	1.059	0.045	0.104	0.630	24.832	41.391	8.002	1	1	0	1	0
134	0.430	9.295	91.549	-0.386	0.986	9.206	1.038	0.045	0.104	0.411	24.054	41.330	8.232	1	1	0	1	0
135	0.447	9.002	54.282	-0.387	0.980	12.716	1.073	0.043	0.109	0.632	27.899	34.974	7.907	1	1	0	1	0
136	0.310	9.268	90.209	-0.378	0.982	6.649	1.003	0.020	0.109	0.408	27.988	26.672	7.908	1	1	0	1	0
137	0.274	1.737	82.631	-0.368	0.980	12.549	1.059	0.045	0.107	0.632	29.420	41.586	8.007	1	1	0	1	1
138	0.282	8.805	85.946	-0.371	0.926	5.862	0.971	0.014	0.103	0.462	24.451	31.849	8.123	0	1	0	0	0
139	0.212	8.430	69.126	-0.390	0.945	11.825	1.128	0.054	0.107	0.604	24.195	40.468	7.863	1	1	0	1	0
140	0.289	10.871	59.321	-0.373	1.205	12.092	1.189	0.046	0.107	0.631	30.237	34.757	7.972	1	1	0	1	1
141	0.255	9.450	85.404	-0.386	0.929	8.295	0.952	0.054	0.115	0.391	26.012	27.902	7.909	1	1	0	0	0
142	0.291	9.678	56.859	-0.388	0.917	11.283	1.016	0.110	0.103	0.360	25.883	40.611	7.854	1	1	0	0	0
143	0.294	9.119	82.972	-0.378	0.963	6.855	1.199	0.036	0.086	0.606	26.122	36.212	8.207	1	1	0	0	0
144	0.211	8.596	88.157	-0.370	0.785	11.116	0.686	0.034	0.101	0.379	26.039	43.218	7.893	1	1	0	1	1
145	0.392	9.241	85.528	-0.390	1.017	13.406	1.108	0.046	0.099	0.361	27.475	42.561	7.923	1	1	0	1	1
146	0.358	9.160	87.439	-0.359	1.033	16.893	0.974	0.014	0.099	0.376	28.100	44.716	8.318	0	1	0	1	1
147	0.364	10.337	91.909	-0.397	0.876	3.488	1.089	0.046	0.107	0.377	22.587	31.479	8.022	1	1	0	1	0
148	0.441	9.063	85.029	-0.383	0.798	7.020	1.065	0.027	0.105	0.390	27.782	12.454	8.147	1	1	0	1	0
149	0.282	2.502	83.105	-0.369	1.241	7.893	1.280	0.049	0.107	0.458	27.413	42.518	7.925	1	1	0	1	1
150	0.340	9.807	77.259	-0.376	1.070	5.968	1.006	0.058	0.103	0.480	28.655	38.008	8.131	1	1	0	0	0
151	0.250	8.962	86.435	-0.393	0.934	7.331	1.069	0.014	0.105	0.602	32.405	109.162	7.887	1	1	0	0	1
152	0.421	9.591	94.971	-0.361	1.036	9.824	1.103	0.014	0.105	0.586	25.549	12.454	7.850	1	1	0	0	0
153	0.430	11.001	93.843	-0.350	0.967	11.920	1.063	0.041	0.102	0.229	22.733	43.567	7.926	1	1	0	1	1

Solution ID	Molecular descriptors 1–12												Predicted endpoint outputs (6)					
	ATSc1	Lipoaffinity Index	sumI	BCUTc-1L	SCH-7	WTPT-3	SHBd	VCH-6	ETA_BetaP_ns_d	maxHBd	SsOH	TopoPSA	pIC_{50}	Caco-2	CYP3A4	hERG	HOB	MN
154	0.420	8.315	63.522	-0.374	1.033	7.822	1.028	0.038	0.088	0.397	29.805	41.674	7.885	1	1	0	1	0
155	0.410	9.501	56.115	-0.368	0.942	6.521	1.002	0.053	0.109	0.322	22.962	43.948	7.849	1	1	1	0	0

Table S1. Cont. Molecular descriptors 13–24 and repeated predicted endpoint outputs.

Solution ID	Molecular descriptors 13–24												Predicted endpoint outputs (6)					
	maxsOH	C2SP2	minsOH	FMF	minHBd	maxHCsats	nBondsD	minHBa	VC-3	ETA_EtaP	MLogP	nsssCH	pIC_{50}	Caco-2	CYP3A4	hERG	HOB	MN
1	9.371	12.715	10.537	0.436	0.707	0.552	10.680	-1.401	0.724	0.826	3.422	0.522	8.050	0	1	0	1	0
2	9.319	12.697	9.573	0.452	0.576	0.655	10.383	0.349	1.213	0.937	3.570	0.220	8.120	1	1	0	1	0
3	10.083	13.812	10.456	0.438	0.521	0.445	10.558	-0.122	0.723	0.784	5.199	1.064	8.421	1	1	0	1	1
4	8.249	14.167	10.036	0.438	0.581	0.703	10.626	0.454	1.111	0.835	4.679	0.345	7.890	0	1	0	1	0
5	10.002	11.304	9.647	0.435	0.570	0.669	9.126	-0.054	1.111	0.773	5.001	0.360	8.236	1	1	0	0	0
6	9.298	11.604	9.744	0.460	0.639	0.700	10.974	0.134	0.671	0.708	4.900	0.017	7.981	1	1	0	1	0
7	9.355	9.301	9.115	0.467	0.586	0.711	10.730	0.118	0.611	0.847	5.245	0.021	8.024	1	1	0	1	0
8	10.022	11.330	10.038	0.382	0.577	0.694	9.981	0.109	0.763	0.847	3.408	0.036	8.091	1	1	0	0	0
9	10.027	9.464	9.831	0.465	0.586	0.702	9.927	-0.054	0.692	0.836	4.968	0.010	8.480	1	1	0	0	0
10	9.445	11.716	9.714	0.429	0.579	0.810	10.658	-0.845	0.948	0.819	5.050	0.189	8.115	0	1	0	1	0
11	10.014	9.249	9.750	0.299	0.458	0.654	10.675	-0.128	0.952	0.848	5.500	0.044	7.877	0	1	0	1	0
12	7.774	10.721	10.037	0.438	0.574	0.676	9.040	-0.071	0.931	0.847	4.884	0.069	8.256	1	1	0	1	0
13	9.990	11.296	10.195	0.447	0.570	0.757	10.388	-0.080	0.947	0.774	5.001	0.158	8.240	0	1	0	0	0
14	8.279	14.479	10.047	0.448	0.595	0.669	10.546	0.040	0.604	0.760	4.674	0.120	7.965	1	1	0	1	0
15	8.906	11.817	9.547	0.569	0.577	0.668	10.674	-0.563	0.847	0.823	4.898	0.357	8.288	0	1	0	1	0
16	9.963	11.323	10.446	0.425	0.577	0.700	9.981	-0.560	0.763	0.835	3.407	0.044	8.016	1	1	0	0	1
17	8.249	14.794	10.036	0.438	0.581	0.646	10.888	0.454	1.114	0.845	3.690	0.024	8.162	0	1	0	1	0
18	8.381	13.809	10.022	0.380	0.600	0.676	10.678	-0.570	0.740	0.839	4.962	1.418	8.382	0	1	0	1	0
19	9.112	8.844	9.758	0.383	0.570	0.647	10.457	0.138	1.153	0.770	5.352	0.021	7.896	1	1	0	1	0
20	8.294	9.416	10.027	0.425	0.594	0.754	10.044	0.093	0.649	0.853	5.046	0.019	8.247	1	1	0	1	0
21	9.207	9.627	10.304	0.471	0.530	0.644	10.497	-0.040	0.949	0.783	3.397	0.226	7.904	1	1	0	0	0
22	10.017	9.356	10.048	0.448	0.601	0.670	10.389	0.120	0.611	0.776	3.771	0.116	8.249	1	1	0	0	0
23	8.291	9.397	10.041	0.414	0.600	0.703	10.045	0.092	0.601	0.786	4.924	0.038	8.376	1	1	0	1	0
24	9.328	9.216	10.027	0.438	0.577	0.646	9.162	-0.102	0.800	0.776	5.483	0.008	8.016	1	1	0	1	0
25	7.995	10.706	10.036	0.438	0.574	0.655	9.994	-0.121	0.988	0.848	3.360	0.209	8.275	1	1	0	1	0
26	9.332	10.664	10.078	0.586	0.566	0.759	10.555	-0.214	0.947	0.784	4.968	0.035	8.267	1	1	0	1	0
27	10.010	10.137	10.053	0.434	0.570	0.552	9.086	-1.401	0.723	0.826	3.422	0.531	8.071	1	1	0	0	0
28	9.344	10.786	10.039	0.438	0.638	0.702	9.613	0.112	1.110	0.846	3.486	0.008	7.961	1	1	0	1	0
29	9.282	11.631	9.576	0.439	0.579	0.697	10.677	-0.559	0.847	0.780	4.896	1.412	8.428	1	1	0	1	0
30	9.415	13.024	10.297	0.459	0.609	0.590	10.373	-1.029	1.107	0.838	5.197	0.032	8.012	1	1	0	1	1
31	9.332	10.753	10.055	0.433	0.566	0.754	10.555	-0.215	0.947	0.784	4.958	0.035	8.214	1	1	0	1	0

Solution ID	Molecular descriptors 13–24												Predicted endpoint outputs (6)					
	maxsOH	C2SP2	minsOH	FMF	minHBd	maxHCsats	nBondsD	minHBa	VC-3	ETA_EtaP	MLogP	nsssCH	pIC_{50}	Caco-2	CYP3A4	hERG	HOB	MN
32	9.298	11.600	9.576	0.441	0.639	0.697	10.657	0.125	0.839	0.713	4.896	1.412	8.145	1	1	0	1	0
33	9.989	9.647	9.713	0.566	0.600	0.664	9.613	0.118	0.606	0.785	4.943	0.403	8.318	1	1	0	1	1
34	9.263	11.817	10.292	0.434	0.577	0.668	10.673	0.045	0.848	0.826	4.898	0.347	8.233	0	1	0	1	0
35	9.335	9.632	10.297	0.595	0.577	0.664	9.640	-0.124	0.983	0.838	5.197	0.019	7.976	1	1	0	1	1
36	7.774	10.721	10.037	0.443	0.573	0.457	9.036	-1.095	0.931	0.848	4.884	0.069	8.135	1	1	0	1	0
37	9.310	9.352	9.722	0.416	0.600	0.788	9.863	0.140	0.631	0.785	5.129	0.057	7.919	1	1	0	1	0
38	9.335	13.392	10.047	0.384	0.571	0.834	9.898	0.133	0.676	0.843	3.452	0.030	7.997	1	1	0	1	0
39	9.989	9.674	9.735	0.560	0.585	0.664	10.678	0.118	0.650	0.849	4.707	0.403	8.367	1	1	0	1	1
40	9.344	10.786	10.039	0.438	0.569	0.702	9.571	0.115	1.109	0.846	4.938	0.008	7.967	1	1	0	1	0
41	9.404	13.024	10.297	0.459	0.577	0.590	10.390	0.149	1.107	0.838	5.197	0.032	8.306	1	1	0	1	0
42	9.298	11.600	9.737	0.460	0.639	0.654	10.974	0.140	0.839	0.708	4.896	0.017	8.120	1	1	0	1	0
43	9.365	8.888	10.540	0.469	0.598	0.715	10.672	0.120	0.653	0.781	3.338	0.418	8.004	1	1	0	1	0
44	9.344	10.718	10.039	0.438	0.638	0.702	9.985	0.112	1.110	0.848	3.486	1.139	8.000	1	1	0	1	0
45	8.365	11.873	10.029	0.363	0.541	0.700	10.588	-0.429	0.667	0.759	4.899	0.028	8.004	1	1	0	1	0
46	9.359	10.646	10.078	0.449	0.574	0.657	10.555	-0.210	0.855	0.784	4.980	0.239	8.340	1	1	0	1	0
47	10.010	10.137	10.056	0.438	0.570	0.443	10.803	-1.011	0.722	0.826	3.336	0.148	8.143	1	1	0	0	0
48	8.249	9.398	10.040	0.439	0.582	0.703	10.045	0.350	0.960	0.835	3.446	0.019	8.061	1	1	0	1	0
49	9.324	11.874	10.076	0.417	0.566	0.652	10.050	0.079	0.625	0.784	3.446	0.052	7.987	1	1	0	1	1
50	9.348	8.814	9.080	0.448	0.589	0.651	10.684	0.112	0.635	0.845	4.924	0.520	8.430	1	1	0	0	0
51	8.358	8.822	10.063	0.431	0.578	0.702	12.129	0.100	0.763	0.849	4.900	0.010	8.056	1	1	0	1	1
52	9.386	9.114	9.102	0.467	0.577	0.648	10.747	0.120	0.647	0.847	3.450	0.032	7.944	1	1	0	1	0
53	7.855	12.774	10.037	0.564	0.587	0.664	9.040	0.335	0.931	0.847	4.897	0.068	8.458	1	1	0	1	0
54	8.320	9.398	10.040	0.454	0.594	0.703	10.045	0.350	0.960	0.835	3.446	0.019	8.102	1	1	0	1	1
55	7.848	12.774	10.243	0.563	0.587	0.664	9.040	0.132	1.059	0.841	3.352	0.026	8.169	0	1	0	1	1
56	8.305	9.537	10.043	0.385	0.586	0.650	10.548	-0.412	0.947	0.847	5.291	0.067	8.018	1	1	0	1	0
57	9.356	9.499	10.058	0.584	0.578	0.702	10.391	0.220	0.633	0.829	3.413	0.024	8.241	1	1	0	1	1
58	8.646	15.127	10.058	0.569	0.531	0.702	9.760	-0.122	0.671	0.828	4.846	0.163	8.088	1	1	0	1	1
59	9.335	9.632	10.559	0.425	0.577	0.664	9.643	-0.124	0.983	0.846	5.197	0.020	7.913	1	1	0	1	0
60	9.373	9.492	10.540	0.471	0.598	0.650	10.672	0.120	0.720	0.847	4.933	0.041	8.083	0	1	0	1	0
61	9.330	10.665	10.078	0.448	0.566	0.703	12.122	-0.210	0.855	0.784	4.900	0.035	8.159	1	1	0	1	1
62	9.320	11.276	9.113	0.453	0.578	0.663	10.545	0.613	0.642	0.827	5.054	0.861	7.855	1	1	0	1	0
63	7.865	12.774	10.060	0.432	0.577	0.664	9.040	-0.121	0.936	0.847	5.186	0.065	8.203	1	1	0	1	0

Solution ID	Molecular descriptors 13–24												Predicted endpoint outputs (6)					
	maxsOH	C2SP2	minsOH	FMF	minHBd	maxHCsats	nBondsD	minHBa	VC-3	ETA_EtaP	MLogP	nsssCH	pIC_{50}	Caco-2	CYP3A4	hERG	HOB	MN
64	7.811	13.019	10.037	0.434	0.587	0.668	9.307	0.112	0.931	0.847	4.897	0.068	8.197	1	1	0	1	0
65	9.916	11.547	10.058	0.584	0.531	0.702	9.748	0.219	0.670	0.808	5.109	0.163	8.054	1	1	0	1	1
66	9.236	10.679	10.036	0.446	0.601	0.653	9.995	0.111	0.621	0.846	3.701	0.043	7.960	1	1	0	1	0
67	9.318	11.541	9.573	0.453	0.581	0.655	10.402	-0.201	1.217	1.125	5.058	0.381	8.447	1	1	0	0	0
68	8.249	14.543	10.017	0.433	0.580	0.703	10.443	0.112	0.805	0.783	4.838	0.343	8.159	1	1	0	1	0
69	10.014	9.674	9.736	0.560	0.585	0.664	10.678	0.118	0.650	0.849	4.929	0.403	8.399	1	1	0	1	0
70	10.022	11.191	9.769	0.387	0.578	0.704	10.328	-0.228	0.664	0.847	3.499	0.010	8.174	1	1	0	1	0
71	9.359	11.224	9.115	0.467	0.586	0.648	10.730	0.118	0.648	0.774	5.282	0.021	8.079	1	1	0	1	0
72	8.280	11.448	11.507	0.398	0.583	0.645	9.228	0.217	0.709	0.774	5.108	0.399	7.848	1	1	0	1	0
73	8.361	8.817	10.063	0.309	0.578	0.683	10.677	0.100	0.763	0.759	5.107	0.011	7.837	1	1	0	1	0
74	9.990	8.822	9.648	0.430	0.570	0.645	9.105	-0.079	0.942	0.774	5.001	0.156	8.135	1	1	0	1	0
75	9.312	10.343	9.754	0.449	0.577	0.699	10.130	-0.079	0.672	0.778	3.497	0.391	8.002	1	1	0	1	1
76	10.013	9.624	9.707	0.387	0.530	0.704	10.328	-0.228	0.664	0.844	3.362	0.012	8.107	1	1	0	1	0
77	10.036	11.288	9.522	0.321	0.577	0.715	9.980	0.120	0.763	0.843	3.408	0.036	7.939	1	1	0	0	0
78	8.650	9.389	9.573	0.454	0.594	0.703	10.045	0.350	0.959	0.779	3.007	0.019	7.836	1	1	0	1	0
79	9.404	11.910	10.047	0.417	0.565	0.661	10.085	0.055	0.619	0.792	3.325	0.000	7.962	1	1	0	1	1
80	10.002	11.304	9.750	0.435	0.570	0.791	10.551	-0.054	1.111	0.774	5.001	0.362	8.144	1	1	0	0	0
81	9.322	13.638	10.425	0.469	0.586	0.667	10.672	0.116	0.970	0.835	3.451	0.420	7.834	0	1	0	1	1
82	10.086	13.829	9.986	0.438	0.521	0.445	10.558	-0.122	0.724	0.784	5.199	1.081	8.053	1	1	0	1	0
83	7.811	13.019	10.037	0.389	0.587	0.668	9.313	-0.208	0.947	0.837	4.897	0.067	8.068	1	1	0	1	0
84	9.312	10.357	9.720	0.434	0.578	0.699	10.317	-0.079	0.672	0.706	5.105	0.147	7.884	1	1	0	1	0
85	7.855	12.774	10.040	0.466	0.587	0.648	10.768	0.335	0.647	0.847	4.880	0.068	8.416	1	1	0	1	0
86	8.340	9.395	9.707	0.387	0.600	0.704	10.328	-0.228	0.601	0.843	3.362	0.013	8.201	1	1	0	1	0
87	10.060	10.664	9.737	0.384	0.566	0.703	12.151	0.156	1.205	0.835	4.658	0.215	8.010	1	1	0	0	0
88	9.331	8.776	10.014	0.325	0.587	0.828	10.677	-0.746	1.132	0.847	4.837	0.521	7.995	1	1	0	1	0
89	8.428	10.677	10.062	0.473	0.708	0.676	10.631	0.144	0.972	0.827	4.929	0.522	7.879	0	1	0	1	0
90	9.323	9.000	9.583	0.431	0.570	0.702	9.869	0.097	0.768	0.849	4.934	0.373	8.149	1	1	0	1	0
91	8.448	12.774	10.243	0.563	0.587	0.664	9.042	0.118	0.846	0.824	4.886	0.026	8.266	1	1	0	1	0
92	10.219	9.499	10.058	0.584	0.578	0.666	10.647	0.220	0.633	0.747	3.413	0.024	7.851	1	1	0	1	1
93	9.276	10.726	10.283	0.434	0.581	0.655	10.497	0.037	1.615	0.845	5.093	0.012	7.955	1	1	0	1	0
94	9.984	10.634	9.736	0.447	0.585	0.664	10.678	0.118	0.650	0.849	4.929	0.403	8.364	1	1	0	0	0
95	8.382	11.627	9.742	0.382	0.639	0.700	10.974	0.134	0.671	0.844	4.900	0.017	8.085	1	1	0	1	0

Solution ID	Molecular descriptors 13–24												Predicted endpoint outputs (6)					
	maxsOH	C2SP2	minsOH	FMF	minHBd	maxHCsats	nBondsD	minHBa	VC-3	ETA_EtaP	MLogP	nsssCH	pIC_{50}	Caco-2	CYP3A4	hERG	HOB	MN
96	10.008	9.475	10.019	0.579	0.600	0.654	10.577	0.105	0.942	0.711	4.931	0.378	8.065	0	1	0	1	1
97	9.989	11.671	9.577	0.563	0.577	0.698	10.580	-0.384	1.116	0.778	4.707	0.403	8.278	0	1	0	1	0
98	10.013	9.620	9.663	0.433	0.530	0.703	10.340	-0.123	0.664	0.844	3.397	0.012	8.167	1	1	0	0	0
99	9.316	10.727	9.663	0.432	0.577	0.703	11.691	-0.123	0.981	0.836	4.672	1.141	8.074	1	1	0	1	0
100	9.355	11.343	9.115	0.467	0.586	0.711	10.734	0.121	0.611	0.847	5.245	0.021	7.888	1	1	0	1	0
101	9.227	11.254	10.041	0.438	0.601	0.700	10.016	0.059	1.095	0.848	4.958	0.038	8.163	1	1	0	0	0
102	8.242	9.506	9.998	0.431	0.600	0.664	9.613	0.118	0.603	0.836	4.891	0.327	8.199	1	1	0	1	0
103	10.088	13.850	9.065	0.586	0.585	0.746	10.555	-0.210	0.974	0.838	4.925	0.035	8.385	1	1	0	1	0
104	9.375	9.674	9.123	0.563	0.577	0.664	10.580	-0.384	0.648	0.771	4.707	0.403	8.160	1	1	0	1	0
105	9.356	9.657	10.059	0.443	0.570	0.702	10.391	0.222	0.633	0.848	3.413	0.024	8.248	1	1	0	1	0
106	8.320	9.398	10.058	0.581	0.594	0.703	10.045	0.350	0.960	0.808	5.212	0.164	8.346	1	1	0	1	1
107	9.335	9.632	10.047	0.595	0.577	0.664	9.640	-0.124	0.987	0.838	5.197	0.019	8.073	1	1	0	1	0
108	10.014	9.636	9.741	0.425	0.579	0.664	10.685	-0.123	0.960	0.848	5.500	0.054	7.845	0	1	0	1	0
109	9.344	10.786	10.047	0.438	0.638	0.834	9.892	0.133	0.677	0.846	3.486	0.008	7.932	1	1	0	1	0
110	9.338	11.449	11.421	0.398	0.562	0.660	9.228	0.217	0.709	0.785	5.108	0.399	7.965	1	1	0	1	0
111	9.282	11.641	10.044	0.439	0.579	0.697	10.677	-0.559	0.849	0.836	4.896	1.412	8.607	0	1	0	1	0
112	8.279	14.479	10.048	0.505	0.595	0.669	10.398	0.038	0.625	0.760	4.716	0.120	8.005	1	1	0	1	0
113	9.373	9.492	9.576	0.471	0.576	0.654	10.672	0.120	1.209	0.847	4.933	0.221	8.355	0	1	0	0	0
114	8.908	9.647	9.713	0.569	0.600	0.665	10.029	0.115	0.606	0.836	4.943	0.403	8.353	1	1	0	1	0
115	8.355	11.251	10.041	0.442	0.542	0.690	10.580	0.102	1.246	0.784	5.290	0.004	8.029	1	1	0	1	0
116	9.236	10.786	10.036	0.446	0.601	0.653	9.995	0.111	0.621	0.846	3.701	0.043	7.836	1	1	0	1	1
117	9.964	9.627	10.041	0.414	0.530	0.703	10.045	0.092	0.665	0.787	4.924	0.038	8.340	1	1	0	1	0
118	10.262	11.196	10.670	0.424	0.571	0.693	10.741	0.129	0.644	0.833	5.021	0.016	8.090	1	1	0	0	0
119	9.307	11.224	10.123	0.467	0.586	0.648	10.730	0.118	0.864	0.774	5.281	0.021	8.132	0	1	0	1	1
120	9.404	9.418	9.829	0.459	0.577	0.588	10.390	0.149	0.987	0.840	3.774	0.032	8.180	1	1	0	1	1
121	9.372	13.611	10.038	0.582	0.576	0.645	9.133	-0.077	0.983	0.776	5.203	0.018	8.130	0	1	0	1	1
122	9.389	9.686	10.078	0.562	0.574	0.657	10.555	-0.210	0.855	0.784	4.980	0.239	8.405	1	1	0	1	0
123	9.298	13.787	10.025	0.458	0.600	0.676	10.678	-0.570	0.740	0.703	4.962	1.418	8.369	1	1	0	1	0
124	9.314	10.758	9.708	0.387	0.577	0.704	11.680	-0.229	0.981	0.836	5.156	1.154	7.985	1	1	0	1	0
125	9.336	13.637	10.033	0.438	0.577	0.645	9.162	-0.103	0.800	0.776	5.205	0.008	8.257	1	1	0	1	0
126	10.027	9.477	9.837	0.316	0.586	0.790	10.575	-0.054	0.692	0.836	4.900	0.010	8.027	1	1	0	1	0
127	9.400	12.655	9.705	0.345	0.586	0.663	10.647	0.139	0.724	0.749	4.892	0.027	8.015	1	1	0	1	0

Solution ID	Molecular descriptors 13–24												Predicted endpoint outputs (6)					
	maxsOH	C2SP2	minsOH	FMF	minHBd	maxHCsats	nBondsD	minHBa	VC-3	ETA_EtaP	MLogP	nsssCH	pIC_{50}	Caco-2	CYP3A4	hERG	HOB	MN
128	9.348	8.814	9.080	0.448	0.589	0.651	10.684	0.112	0.607	0.845	5.078	0.520	8.424	1	1	0	0	0
129	7.855	13.344	10.038	0.564	0.587	0.691	9.040	0.335	0.981	0.847	4.897	0.918	8.463	1	1	0	1	0
130	8.409	9.495	10.201	0.587	0.579	0.664	9.613	0.117	0.771	0.829	3.699	0.097	8.132	1	1	0	1	0
131	9.916	11.499	10.040	0.457	0.531	0.702	9.748	0.219	0.670	0.841	3.343	0.018	7.873	1	1	0	1	1
132	8.362	8.848	10.049	0.467	0.578	0.716	10.662	0.100	0.950	0.832	4.870	0.184	7.958	0	1	0	1	0
133	8.424	10.686	10.062	0.438	0.638	0.674	9.985	0.112	1.110	0.848	3.528	1.662	8.002	1	1	0	1	0
134	9.324	10.717	10.076	0.417	0.566	0.652	10.050	0.079	0.625	0.784	3.446	0.208	8.232	1	1	0	1	0
135	9.335	13.392	10.038	0.399	0.571	0.702	9.619	0.112	1.109	0.843	3.452	0.030	7.907	1	1	0	1	0
136	8.249	14.543	10.303	0.433	0.530	0.703	10.681	0.122	0.805	0.783	4.796	0.343	7.908	1	1	0	1	0
137	9.360	10.718	10.028	0.443	0.645	0.702	9.969	-0.825	0.950	0.848	3.486	1.160	8.007	1	1	0	1	1
138	9.480	11.966	9.766	0.441	0.576	0.843	10.771	-1.217	0.935	0.871	5.131	0.000	8.123	0	1	0	0	0
139	7.821	10.992	10.946	0.424	0.575	0.755	10.192	0.000	0.445	0.857	3.666	0.000	7.863	1	1	0	1	0
140	9.266	11.346	10.655	0.586	0.584	0.676	10.078	-0.527	0.968	0.778	5.156	0.237	7.972	1	1	0	1	1
141	10.279	10.785	10.210	0.454	0.640	0.602	10.477	0.531	0.732	0.687	4.930	1.395	7.909	1	1	0	0	0
142	9.471	10.709	10.225	0.387	0.551	0.867	10.443	-2.455	0.838	0.658	5.061	0.439	7.854	1	1	0	0	0
143	8.920	8.427	9.142	0.453	0.744	0.643	9.956	0.169	0.663	0.835	4.889	0.449	8.207	1	1	0	0	0
144	7.829	12.556	9.347	0.551	0.619	0.559	9.376	0.009	0.994	0.830	3.186	0.027	7.893	1	1	0	1	1
145	9.974	8.440	9.431	0.589	0.579	0.747	10.348	0.470	0.660	0.820	3.352	0.000	7.923	1	1	0	1	1
146	9.995	13.044	9.838	0.459	0.562	0.661	9.594	-1.719	0.943	1.114	3.446	0.859	8.318	0	1	0	1	1
147	8.339	12.968	9.189	0.438	0.550	0.652	10.150	0.347	0.928	0.869	4.768	0.022	8.022	1	1	0	1	0
148	6.675	13.150	10.670	0.472	0.587	0.784	11.784	-0.491	0.493	0.864	4.732	0.463	8.147	1	1	0	1	0
149	9.912	9.527	9.081	0.463	0.590	0.561	11.048	0.177	0.583	0.842	3.488	0.397	7.925	1	1	0	1	1
150	10.118	9.423	8.799	0.449	0.539	0.633	10.546	-0.085	0.671	0.890	4.895	0.224	8.131	1	1	0	0	0
151	8.294	12.148	8.803	0.444	0.571	0.745	10.049	-1.611	0.603	0.785	4.078	0.000	7.887	1	1	0	0	1
152	9.206	12.859	7.547	0.428	0.615	0.717	9.723	-0.587	1.112	0.834	5.000	0.139	7.850	1	1	0	0	0
153	8.688	9.172	10.183	0.571	0.595	0.686	9.755	-1.207	0.886	0.762	5.493	0.130	7.926	1	1	0	1	1
154	9.388	9.812	9.577	0.652	0.626	0.637	10.023	-0.546	0.547	0.869	5.025	0.000	7.885	1	1	0	1	0
155	10.860	7.633	10.402	0.403	0.488	0.780	10.753	2.422	0.679	0.827	5.125	2.144	7.849	1	1	1	0	0

Notes: Rows in the two horizontal blocks correspond by Solution ID; the six endpoint outputs are repeated in the second block to facilitate interpretation. Solution ID is used only for identification and does not indicate the integrated priority ranking. Molecular descriptor values were back-transformed to their original scales. pIC_{50} is a continuous predicted value; Caco-2, CYP3A4, hERG, HOB, and MN are binary predicted classes. Coding: Caco-2, 1 = relatively good cellular permeability and 0 = relatively poor cellular permeability; CYP3A4, 1 = metabolized by CYP3A4 and 0 = not metabolized by CYP3A4; hERG, 1 = cardiac toxicity present and 0 = absent; HOB, 1 = relatively good human oral bioavailability and 0 = relatively poor human oral bioavailability; MN, 1 =

genotoxicity present and 0 = absent. Thus, class 1 does not uniformly denote a favorable endpoint. The listed solutions are computational outputs and do not imply molecular-structure reconstruction, chemical synthesis, or experimental validation. Abbreviations: CYP3A4, cytochrome P450 3A4; hERG, human ether-a-go-go-related gene; HOB, human oral bioavailability; MN, micronucleus; 2P-HAMOEA, two-phase hybrid adaptive multi-objective evolutionary algorithm. During optimization, the class-1 probabilities were used as continuous objectives with endpoint-specific directions; a 0.5 cutoff was used to obtain the binary classes displayed in this table and to count target-state satisfaction in the feasibility criterion. For hERG, the optimization output was the arithmetic mean of the five constituent class-1 probabilities; this continuous probability mean is distinct from the average of thresholded binary predictions used for the classification-evaluation confusion matrix.

Table S2. Distributional intervals of 24 key molecular descriptors among high-scoring Pareto-optimal solutions.

Descriptor	Descriptive statistics		Core interval (P25–P75)		Robust interval (P10–P90)		Extended interval (mean ± SD)	
	Mean	SD	Lower	Upper	Lower	Upper	Reported lower	Reported upper
ATSc1	0.381	0.067	0.309	0.43	0.288	0.444	0.315	0.448
LipoaffinityIndex	9.246	0.268	9.099	9.417	9.004	9.632	8.978	9.514
sumI	77.503	12.614	62.124	87.733	59.029	88.328	64.889	90.118
BCUTc-1L	-0.373	0.008	-0.379	-0.367	-0.38	-0.363	-0.381	-0.364
SCH-7	0.972	0.06	0.97	1.002	0.932	1.042	0.912	1.031
WTPT-3	8.409	2.532	6.315	9.977	4.05	11.553	5.877	10.94
SHBd	1.053	0.056	1.029	1.067	0.978	1.14	0.997	1.109
VCH-6	0.04	0.01	0.04	0.046	0.019	0.046	0.029	0.05
ETA_BetaP_ns_d	0.105	0.003	0.103	0.109	0.103	0.109	0.103	0.108
maxHBd	0.405	0.073	0.357	0.415	0.319	0.486	0.332	0.478
SsOH	27.08	1.773	25.286	27.979	24.357	28.566	25.307	28.854
TopoPSA	39.69	4.11	38.014	41.71	33.148	42.369	35.58	43.8
maxsOH	8.947	0.756	8.305	9.367	7.855	10.014	8.191	9.702
C2SP2	11.224	1.72	9.652	12.774	9.398	13.787	9.505	12.944
minsOH	9.912	0.332	9.784	10.06	9.547	10.078	9.58	10.244
FMF	0.482	0.069	0.435	0.563	0.413	0.569	0.414	0.551
minHBd	0.58	0.018	0.574	0.588	0.566	0.6	0.561	0.598
maxHCsats	0.671	0.054	0.657	0.702	0.648	0.704	0.617	0.725
nBondsD	10.128	0.603	9.96	10.569	9.04	10.678	9.525	10.731
minHBa	-0.063	0.275	-0.212	0.118	-0.559	0.335	-0.338	0.212
VC-3	0.792	0.15	0.648	0.934	0.606	0.974	0.642	0.943
ETA_EtaP	0.815	0.035	0.784	0.846	0.78	0.847	0.779	0.85
MLogP	4.767	0.549	4.889	4.968	3.446	5.197	4.218	5.315
nsssCH	0.331	0.441	0.035	0.403	0.024	1.064	-0.11	0.772

Notes: The high-scoring subset comprised the top 20% of feasible Pareto-optimal solutions (n = 31), ranked by the robust weighted score (RWS). Core and robust intervals correspond to the 25th-75th percentiles (P25–P75) and 10th-90th percentiles (P10–P90), respectively. Extended intervals are the reported endpoints of the mean ± standard deviation (SD); these endpoints were retained as reported and were not recalculated from the rounded mean and SD values shown in the table. The intervals summarize the distribution of computationally optimized solutions and should not be interpreted as experimentally validated recommended ranges or deterministic structural thresholds. Abbreviation: RWS, robust weighted score.

Table S3. Sensitivity of descriptor intervals to the high-scoring-subset threshold.

Panel A. Core intervals (P25–P75)

Descriptor	Top 10% (n=16)	Top 20% (n=31)	Top 30% (n=47)
ATSc1	0.2910 to 0.4295	0.3091 to 0.4303	0.3108 to 0.4301
LipoaffinityIndex	9.1282 to 9.4860	9.0991 to 9.4167	9.0774 to 9.4221
sumI	59.2647 to 86.5012	62.1237 to 87.7329	64.3962 to 87.7329
BCUTc-1L	-0.3788 to -0.3671	-0.3792 to -0.3670	-0.3794 to -0.3671
SCH-7	0.9620 to 0.9863	0.9696 to 1.0024	0.9698 to 1.0025
WTPT-3	6.4336 to 9.6487	6.3151 to 9.9766	6.2929 to 9.3129
SHBd	1.0290 to 1.0734	1.0289 to 1.0675	1.0284 to 1.0640
VCH-6	0.0429 to 0.0458	0.0402 to 0.0458	0.0331 to 0.0458
ETA_BetaP_ns_d	0.1034 to 0.1086	0.1030 to 0.1086	0.1030 to 0.1086
maxHBd	0.3342 to 0.4104	0.3569 to 0.4152	0.3594 to 0.4096
SsOH	27.4118 to 27.9913	25.2862 to 27.9789	25.9963 to 28.0284
TopoPSA	39.0709 to 41.5925	38.0140 to 41.7102	36.6240 to 41.5968
maxsOH	7.9599 to 9.5327	8.3054 to 9.3673	8.2924 to 9.3964
C2SP2	9.6676 to 12.7743	9.6522 to 12.7743	9.6522 to 12.7741
minsOH	9.8075 to 10.0409	9.7836 to 10.0597	9.7356 to 10.0494
FMF	0.4386 to 0.5642	0.4353 to 0.5630	0.4327 to 0.5599
minHBd	0.5742 to 0.5866	0.5741 to 0.5880	0.5743 to 0.5940
maxHCsats	0.6622 to 0.7024	0.6573 to 0.7021	0.6606 to 0.7025
nBondsD	9.9770 to 10.5857	9.9602 to 10.5689	9.9602 to 10.6757
minHBa	-0.2102 to 0.1155	-0.2122 to 0.1181	-0.2122 to 0.1181
VC-3	0.6610 to 0.9354	0.6478 to 0.9339	0.6496 to 0.9314
ETA_EtaP	0.7844 to 0.8475	0.7844 to 0.8459	0.7844 to 0.8475
MLogP	4.8967 to 4.9636	4.8887 to 4.9678	4.8844 to 4.9623
nsssCH	0.0379 to 0.4031	0.0349 to 0.4031	0.0348 to 0.4031

Panel B. Robust intervals (P10–P90)

Descriptor	Top 10% (n=16)	Top 20% (n=31)	Top 30% (n=47)
ATSc1	0.2810 to 0.4349	0.2878 to 0.4438	0.2954 to 0.4433
LipoaffinityIndex	8.8971 to 9.6429	9.0038 to 9.6315	8.8361 to 9.6543
sumI	58.6714 to 88.2115	59.0289 to 88.3286	59.0601 to 89.2148
BCUTc-1L	-0.3796 to -0.3652	-0.3800 to -0.3631	-0.3861 to -0.3633

Descriptor	Top 10% (n=16)	Top 20% (n=31)	Top 30% (n=47)
SCH-7	0.8929 to 1.0225	0.9324 to 1.0417	0.8989 to 1.0426
WTPT-3	4.0502 to 11.1927	4.0502 to 11.5528	4.0502 to 11.4545
SHBd	0.9784 to 1.1194	0.9784 to 1.1405	0.9751 to 1.1143
VCH-6	0.0402 to 0.0465	0.0191 to 0.0464	0.0178 to 0.0466
ETA_BetaP_ns_d	0.1030 to 0.1088	0.1030 to 0.1088	0.1030 to 0.1088
maxHBd	0.3190 to 0.4530	0.3190 to 0.4862	0.3342 to 0.4855
SsOH	25.0644 to 28.3170	24.3572 to 28.5663	24.4776 to 28.9300
TopoPSA	33.1493 to 42.0172	33.1480 to 42.3687	33.1445 to 42.3138
maxsOH	7.8551 to 10.0209	7.8551 to 10.0143	7.8551 to 10.0136
C2SP2	9.5452 to 13.5656	9.3982 to 13.7871	9.4092 to 13.6972
minsOH	9.6448 to 10.0777	9.5470 to 10.0777	9.5763 to 10.0777
FMF	0.4257 to 0.5778	0.4135 to 0.5693	0.4135 to 0.5671
minHBd	0.5700 to 0.6001	0.5664 to 0.6001	0.5664 to 0.6003
maxHCsats	0.6560 to 0.7245	0.6477 to 0.7044	0.6467 to 0.7209
nBondsD	9.0405 to 10.6783	9.0405 to 10.6781	9.0414 to 10.6808
minHBa	-0.3865 to 0.3351	-0.5593 to 0.3351	-0.5593 to 0.1780
VC-3	0.6267 to 0.9778	0.6061 to 0.9745	0.6061 to 0.9661
ETA_EtaP	0.7822 to 0.8475	0.7800 to 0.8475	0.7751 to 0.8488
MLogP	4.8824 to 4.9741	3.4458 to 5.1969	3.4775 to 5.1021
nsssCH	0.0348 to 1.1651	0.0237 to 1.0645	0.0152 to 1.2036

Panel C. Extended intervals (mean ± SD)

Descriptor	Top 10% (n=16)	Top 20% (n=31)	Top 30% (n=47)
ATSc1	0.2992 to 0.4384	0.3147 to 0.4480	0.3169 to 0.4454
LipoaffinityIndex	8.9520 to 9.5811	8.9775 to 9.5135	8.9446 to 9.5587
sumI	60.7468 to 87.6538	64.8888 to 90.1175	66.3069 to 91.4133
BCUTc-1L	-0.3798 to -0.3666	-0.3809 to -0.3640	-0.3830 to -0.3641
SCH-7	0.9124 to 1.0206	0.9117 to 1.0314	0.9108 to 1.0335
WTPT-3	5.5696 to 10.8670	5.8769 to 10.9402	5.6732 to 10.5203
SHBd	0.9994 to 1.1068	0.9970 to 1.1090	0.9933 to 1.0982
VCH-6	0.0351 to 0.0503	0.0293 to 0.0499	0.0272 to 0.0494
ETA_BetaP_ns_d	0.1033 to 0.1087	0.1027 to 0.1079	0.1017 to 0.1081
maxHBd	0.3125 to 0.4721	0.3322 to 0.4785	0.3399 to 0.4697

Descriptor	Top 10% (n=16)	Top 20% (n=31)	Top 30% (n=47)
SsOH	25.9745 to 28.7995	25.3068 to 28.8535	25.5020 to 28.9059
TopoPSA	35.8920 to 43.4324	35.5803 to 43.7999	33.2130 to 44.8034
maxsOH	8.0730 to 9.8378	8.1909 to 9.7024	8.1207 to 9.8207
C2SP2	9.5115 to 12.7877	9.5047 to 12.9440	9.5498 to 12.8381
minsOH	9.6306 to 10.1756	9.5799 to 10.2436	9.5944 to 10.2123
FMF	0.4307 to 0.5658	0.4138 to 0.5510	0.4069 to 0.5354
minHBd	0.5637 to 0.5974	0.5615 to 0.5977	0.5610 to 0.6064
maxHCsats	0.6529 to 0.7175	0.6171 to 0.7255	0.6147 to 0.7329
nBondsD	9.5214 to 10.7517	9.5252 to 10.7314	9.5324 to 10.8350
minHBa	-0.3303 to 0.2307	-0.3378 to 0.2117	-0.3709 to 0.2262
VC-3	0.6612 to 0.9529	0.6418 to 0.9429	0.6314 to 0.9364
ETA_EtaP	0.7711 to 0.8544	0.7792 to 0.8502	0.7754 to 0.8566
MLogP	4.4382 to 5.2265	4.2176 to 5.3155	4.2202 to 5.2769
nsssCH	-0.1252 to 0.8255	-0.1100 to 0.7725	-0.1114 to 0.7716

Notes: The same 155 feasible Pareto solutions were ranked in descending order of the PUI-adjusted robust weighted score (RWS). Upward rounding gave subset sizes of 16, 31, and 47 for the Top 10%, Top 20%, and Top 30% analyses, respectively. All descriptor values are reported on their original scales after inverse transformation. Pairwise interval similarity was quantified as intersection length divided by union length using unrounded endpoints. For Top 10% versus Top 30%, the median overlap indices across the 24 descriptors were 0.839 for the core intervals, 0.889 for the robust intervals, and 0.843 for the extended intervals. The robust interval for MLogP and the core and robust intervals for VCH-6 were particularly threshold sensitive; therefore, the intervals should not be interpreted as threshold-invariant or experimentally validated structural limits.

Table S4. Matched comparison of pIC_{50} stacking performance using the current 24-descriptor set and the Dong et al. 20-descriptor set.

Descriptor set	n	MAE	MedAE	MSE	R^2	R^2 95% CI
Current 24 descriptors	24	0.575	0.419	0.633	0.6467	0.5428–0.7248
Dong et al. descriptors	20	0.585	0.448	0.633	0.6465	0.5453–0.7247

Notes: The same fixed MD-FIS compound assignments, three first-layer model families and fixed hyperparameters, preprocessing settings, stacking architecture, and 375-compound internal holdout were used for both descriptor sets. The sets shared BCUTc-1L, LipoaffinityIndex, MLogP, maxsOH, and minsOH. The paired R^2 difference (current 24 minus Dong 20) was 0.00015 (95% paired-bootstrap CI, −0.0478 to 0.0466; 5,000 resamples). This separate matched sensitivity comparison did not detect a material difference between the descriptor sets and did not demonstrate superiority of either set. It does not replace the formally reported final-model estimate in the main manuscript.

Table S5. Original training-set and internal-holdout results for candidate pIC_{50} regression models and ensemble alternatives: (A) training-set tenfold cross-validation and radar-area ranking; (B) internal-holdout comparison of shortlisted models and ensemble configurations.

A. Training-set tenfold cross-validation and radar-area ranking

Model	MAE	MedAE	MSE	R^2	Radar area	Rank
MLP	0.546 ± 0.041	0.423 ± 0.040	0.535 ± 0.091	0.720 ± 0.044	1.928	9
MLP + TPE	0.579 ± 0.047	0.460 ± 0.047	0.571 ± 0.100	0.700 ± 0.054	0.106	13
KNN	0.576 ± 0.047	0.443 ± 0.054	0.586 ± 0.097	0.693 ± 0.046	0.099	14
KNN + TPE	0.548 ± 0.047	0.399 ± 0.039	0.554 ± 0.102	0.710 ± 0.050	1.735	10
SVR	0.573 ± 0.035	0.427 ± 0.046	0.579 ± 0.068	0.696 ± 0.036	0.263	12
SVR + TPE	0.531 ± 0.043	0.404 ± 0.039	0.499 ± 0.082	0.738 ± 0.044	4.390	3
XGBoost	0.560 ± 0.053	0.407 ± 0.050	0.588 ± 0.120	0.692 ± 0.058	0.491	11
XGBoost + TPE	0.529 ± 0.058	0.383 ± 0.054	0.521 ± 0.116	0.727 ± 0.057	4.113	4
RF	0.535 ± 0.048	0.385 ± 0.036	0.530 ± 0.102	0.723 ± 0.049	3.433	7
RF + TPE	0.545 ± 0.047	0.401 ± 0.039	0.534 ± 0.099	0.720 ± 0.049	2.463	8
Oblivious Tree	0.518 ± 0.050	0.385 ± 0.041	0.492 ± 0.104	0.742 ± 0.051	6.102	2
Oblivious Tree + TPE	0.515 ± 0.056	0.369 ± 0.040	0.496 ± 0.112	0.741 ± 0.055	6.659	1
GPR	0.533 ± 0.032	0.414 ± 0.059	0.501 ± 0.066	0.737 ± 0.041	3.913	5
GPR + TPE	0.533 ± 0.032	0.414 ± 0.059	0.501 ± 0.066	0.737 ± 0.041	3.913	6

B. Internal-holdout results for shortlisted models and ensemble configurations

Model	MAE	MedAE	MSE	R^2	Train radar	Holdout radar	Status
Oblivious Tree + TPE	0.608	0.505	0.663	0.630	6.659	2.921	Shortlisted
SVR + TPE	0.626	0.439	0.736	0.589	4.390	2.086	Shortlisted
XGBoost + TPE	0.617	0.442	0.703	0.608	4.113	3.233	Shortlisted
GPR	0.658	0.515	0.751	0.580	3.913	0.265	Shortlisted
RF	0.663	0.476	0.790	0.559	3.433	0.104	Shortlisted
Simple average (top 3)	0.585	0.464	0.631	0.648	-	6.294	Ensemble
Simple average (top 5)	0.603	0.485	0.651	0.636	-	4.098	Ensemble
Softmax-weighted (top 3)	0.584	0.457	0.637	0.644	-	6.328	Ensemble
Softmax-weighted (top 5)	0.587	0.459	0.640	0.643	-	6.037	Ensemble
Stacking (top 3)	0.573	0.421	0.627	0.650	8.081	8.820	Selected
Stacking (top 5)	0.577	0.434	0.629	0.649	7.967	7.971	Ensemble

Notes: These values reproduce the original model-selection results used in this study. The three-model Stacking ensemble comprised Oblivious Tree + TPE, SVR + TPE, and XGBoost + TPE. It had the largest radar area among the training-set ensemble configurations (8.081) and the internal-holdout candidates (8.820), and it achieved the best values of all four holdout metrics. TPE, tree-structured Parzen estimator; GPR, Gaussian process regression.

Table S6. Internal-holdout performance and radar-area summaries of shortlisted ADMET candidate models.

Endpoint	Candidate model	Accuracy	Precision	Recall	AUC	F_1	Status	Radar area train / holdout
Caco-2	GB + TPE	0.853	0.836	0.785	0.932	0.810	Shortlisted	9.358 / 2.843
Caco-2	LightGBM + TPE	0.867	0.851	0.805	0.933	0.828	Selected	9.019 / 7.548
Caco-2	XGBoost + TPE	0.845	0.827	0.772	0.929	0.799	Shortlisted	8.283 / 0.920
Caco-2	BPNN + TPE	0.840	0.850	0.725	0.925	0.783	Shortlisted	7.316 / 0.005
Caco-2	SVM + TPE	0.859	0.829	0.812	0.913	0.820	Shortlisted	5.837 / 1.814
CYP3A4	GB + TPE	0.891	0.917	0.945	0.950	0.931	Shortlisted	7.891 / 0.006
CYP3A4	LightGBM + TPE + CS	0.891	0.908	0.955	0.955	0.931	Shortlisted	6.832 / 0.196
CYP3A4	XGBoost + CS	0.901	0.921	0.955	0.944	0.938	Shortlisted	5.954 / 5.351
CYP3A4	BPNN	0.899	0.915	0.959	0.933	0.936	Shortlisted	4.667 / 3.441
CYP3A4	MLP + TPE + CS	0.904	0.921	0.959	0.936	0.939	Selected	3.811 / 6.750
hERG	GB + TPE	0.813	0.788	0.932	0.893	0.854	Shortlisted	8.900 / 3.386
hERG	XGBoost + TPE	0.811	0.799	0.905	0.891	0.849	Shortlisted	8.499 / 2.021
hERG	LightGBM + TPE	0.813	0.810	0.891	0.893	0.848	Shortlisted	7.613 / 2.152
hERG	BPNN + TPE	0.816	0.803	0.909	0.896	0.853	Shortlisted	7.565 / 3.347
hERG	SVM	0.795	0.754	0.964	0.890	0.846	Shortlisted	5.381 / 0.007
hERG	Five-model simple averaging ensemble	0.829	0.805	0.936	0.878	0.866	Selected	8.955 / 6.716
HOB	LightGBM + TPE + CS	0.797	0.791	0.675	0.871	0.729	Shortlisted	8.137 / 5.157
HOB	XGBoost + TPE + CS	0.800	0.738	0.781	0.870	0.759	Shortlisted	8.068 / 5.030
HOB	GB + TPE	0.797	0.786	0.682	0.880	0.730	Selected	7.792 / 5.540
HOB	BPNN	0.776	0.772	0.629	0.851	0.693	Shortlisted	4.991 / 1.421
HOB	SVM + TPE	0.755	0.736	0.609	0.820	0.667	Shortlisted	4.970 / 0.001
MN	GB + TPE + CS	0.923	0.932	0.973	0.976	0.952	Shortlisted	8.927 / 5.693
MN	LightGBM + CS	0.933	0.938	0.980	0.979	0.958	Selected	8.715 / 8.366
MN	XGBoost	0.925	0.926	0.983	0.975	0.954	Shortlisted	7.976 / 6.466
MN	SVM + TPE	0.888	0.901	0.963	0.898	0.931	Shortlisted	7.113 / 0.002
MN	BPNN + TPE + CS	0.917	0.949	0.946	0.960	0.947	Shortlisted	7.071 / 3.120

Notes: The five individual rows for each endpoint were the highest-ranked candidates retained after equal-importance, normalized multi-metric training-set screening. The radar-area column reproduces the training/internal-holdout summaries from the original model-selection analysis. For HOB, GB + TPE had the largest internal-holdout radar area (5.540) and highest AUC (0.880), whereas XGBoost + TPE + CS had higher recall and F_1. The selected hERG model was the simple average of the five binary constituent predictions; its score was the vote fraction. These values are internal comparative estimates rather than external-validation performance. TPE, tree-structured Parzen estimator; CS, cost-sensitive learning; BPNN, backpropagation neural network.

Table S7. Tenfold cross-validation performance of selected ADMET individual models and hERG ensemble constituents.

Endpoint	Model	Accuracy	Precision	Recall	AUC	F_1
Caco-2	LightGBM + TPE	0.919 ± 0.022	0.899 ± 0.043	0.884 ± 0.040	0.973 ± 0.011	0.891 ± 0.029
CYP3A4	MLP + TPE + CS	0.934 ± 0.017	0.975 ± 0.019	0.932 ± 0.017	0.982 ± 0.013	0.953 ± 0.012
hERG	GB + TPE (constituent)	0.923 ± 0.027	0.925 ± 0.029	0.939 ± 0.029	0.973 ± 0.012	0.932 ± 0.024
hERG	XGBoost + TPE (constituent)	0.923 ± 0.023	0.929 ± 0.028	0.934 ± 0.029	0.975 ± 0.012	0.931 ± 0.020
hERG	LightGBM + TPE (constituent)	0.919 ± 0.015	0.925 ± 0.020	0.932 ± 0.021	0.975 ± 0.008	0.928 ± 0.014
hERG	BPNN + TPE (constituent)	0.921 ± 0.021	0.931 ± 0.033	0.930 ± 0.021	0.971 ± 0.014	0.930 ± 0.018
hERG	SVM (constituent)	0.909 ± 0.026	0.908 ± 0.029	0.932 ± 0.028	0.967 ± 0.016	0.919 ± 0.023
HOB	GB + TPE	0.889 ± 0.017	0.771 ± 0.031	0.699 ± 0.069	0.935 ± 0.017	0.732 ± 0.048
MN	LightGBM + CS	0.959 ± 0.014	0.970 ± 0.012	0.978 ± 0.018	0.987 ± 0.008	0.974 ± 0.009

Notes: Values are validation-fold mean ± standard deviation from tenfold cross-validation within the 1,500-compound training set. For hERG, the archived workflow retained fold-wise summaries for the five constituent learners but not a fold-wise summary for their final simple-average ensemble; the ensemble uncertainty is therefore reported for the internal holdout in Table S8 rather than reconstructed from unavailable fold assignments.

Table S8. Bootstrap 95% confidence intervals for final internal-holdout metrics.

Endpoint	Metric	Estimate	95% CI	Method
pIC_{50}	MAE	0.573	0.519–0.631	5,000-resample nonparametric bootstrap
pIC_{50}	MedAE	0.421	0.355–0.487	5,000-resample nonparametric bootstrap
pIC_{50}	MSE	0.627	0.492–0.788	5,000-resample nonparametric bootstrap
pIC_{50}	R^2	0.650	0.549–0.728	5,000-resample nonparametric bootstrap
Caco-2	Accuracy	0.867	0.832–0.901	10,000-resample nonparametric bootstrap
Caco-2	Precision	0.851	0.788–0.907	10,000-resample nonparametric bootstrap
Caco-2	Recall	0.805	0.740–0.868	10,000-resample nonparametric bootstrap
Caco-2	AUC	0.933	0.908–0.956	10,000-resample nonparametric bootstrap
Caco-2	F_1	0.828	0.777–0.873	10,000-resample nonparametric bootstrap
CYP3A4	Accuracy	0.904	0.872–0.933	10,000-resample nonparametric bootstrap
CYP3A4	Precision	0.921	0.889–0.949	10,000-resample nonparametric bootstrap
CYP3A4	Recall	0.959	0.935–0.980	10,000-resample nonparametric bootstrap
CYP3A4	AUC	0.936	0.905–0.962	10,000-resample nonparametric bootstrap
CYP3A4	F_1	0.939	0.919–0.958	10,000-resample nonparametric bootstrap
hERG	Accuracy	0.829	0.792–0.867	10,000-resample nonparametric bootstrap
hERG	Precision	0.805	0.755–0.851	10,000-resample nonparametric bootstrap
hERG	Recall	0.936	0.903–0.967	10,000-resample nonparametric bootstrap
hERG	AUC	0.878	0.842–0.912	10,000-resample nonparametric bootstrap
hERG	F_1	0.866	0.832–0.896	10,000-resample nonparametric bootstrap
HOB	Accuracy	0.797	0.755–0.837	10,000-resample nonparametric bootstrap
HOB	Precision	0.786	0.713–0.854	10,000-resample nonparametric bootstrap
HOB	Recall	0.682	0.607–0.756	10,000-resample nonparametric bootstrap
HOB	AUC	0.880	0.846–0.912	10,000-resample nonparametric bootstrap
HOB	F_1	0.730	0.669–0.786	10,000-resample nonparametric bootstrap
MN	Accuracy	0.933	0.907–0.957	10,000-resample nonparametric bootstrap
MN	Precision	0.938	0.910–0.964	10,000-resample nonparametric bootstrap
MN	Recall	0.980	0.962–0.993	10,000-resample nonparametric bootstrap
MN	AUC	0.979	0.965–0.989	10,000-resample nonparametric bootstrap
MN	F_1	0.958	0.941–0.974	10,000-resample nonparametric bootstrap

Notes: All intervals were obtained by resampling compounds with replacement within the corresponding 375-compound internal holdout. For hERG, classification-evaluation metrics used the vote fraction from the five thresholded constituent predictions. The intervals represent sampling uncertainty conditional on the fitted and selected models; they do not account for model-selection uncertainty or external chemical-space transport.

Table S9. Leverage-based descriptor-space applicability-domain summary.

Subset	N	Warning leverage h^*	Within AD, n (%)	Above h^*, n (%)	Median leverage	Maximum leverage
Training set	1,500	0.050	1,465 (97.7)	35 (2.3)	0.0135	0.1149
Internal holdout	375	0.050	323 (86.1)	52 (13.9)	0.0300	0.1555

Notes: Leverage was calculated in the shared 24-descriptor space as $h_i = x_i^T(X^TX)^{-1}x_i$, using the 1,500-compound training design matrix with an intercept. The warning threshold was $h^* = 3(p + 1)/n$ = 0.050. Holdout compounds at or below h^* were considered within the training-set descriptor-space AD; values above h^* were flagged as peripheral. The same fixed descriptor matrix and compound assignment were used for all six endpoints. All reported predictive metrics used the full 375-compound holdout. This diagnostic does not establish scaffold-based or external chemical-space applicability. Abbreviation: AD, applicability domain.
